# Interleukin-1 Antagonist Anakinra in Amyotrophic Lateral Sclerosis—A Pilot Study

**DOI:** 10.1371/journal.pone.0139684

**Published:** 2015-10-07

**Authors:** André Maier, Nikolaus Deigendesch, Kathrin Müller, Jochen H. Weishaupt, Alexander Krannich, Robert Röhle, Felix Meissner, Kaaweh Molawi, Christoph Münch, Teresa Holm, Robert Meyer, Thomas Meyer, Arturo Zychlinsky

**Affiliations:** 1 Department of Neurology, Charité-University Hospital, Campus Virchow-Klinikum, Berlin, Germany; 2 Max-Planck Institute for Infection Biology, Berlin, Germany; 3 Neurology Department, Ulm University, Ulm, Germany; 4 Department of Biostatistics, Coordination Center for Clinical Trials, Charité-University Hospital, Berlin, Germany; University of Toronto, CANADA

## Abstract

Preclinical studies show that blocking Interleukin–1 (IL–1) retards the progression of Amyotrophic Lateral Sclerosis (ALS). We assessed the safety of Anakinra (ANA), an IL–1 receptor antagonist, in ALS patients. In a single arm pilot study we treated 17 ALS patients with ANA (100 mg) daily for one year. We selected patients with dominant or exclusive lower motor neuron degeneration (LMND) presentation, as peripheral nerves may be more accessible to the drug. Our primary endpoint was safety and tolerability. Secondary endpoints included measuring disease progression with the revised ALS functional rating scale (ALSFRSr). We also quantified serum inflammatory markers. For comparison, we generated a historical cohort of 47 patients that fit the criteria for enrolment, disease characteristics and rate of progression of the study group. Only mild adverse events occurred in ALS patients treated with ANA. Notably, we observed lower levels of cytokines and the inflammatory marker fibrinogen during the first 24 weeks of treatment. Despite of this, we could not detect a significant reduction in disease progression during the same period in patients treated with ANA compared to controls as measured by the ALSFRSr. In the second part of the treatment period we observed an increase in serum inflammatory markers. Sixteen out of the 17 patients (94%) developed antibodies against ANA. This study showed that blocking IL–1 is safe in patients with ALS. Further trials should test whether targeting IL–1 more efficiently can help treating this devastating disease.

**Trial Registration:**

ClinicalTrials.gov NCT01277315

## Introduction

Amyotrophic lateral sclerosis (ALS) is a progressive degenerative disorder which affects primarily upper motor neurons in the cortex and lower motor neurons in the brain stem and spinal cord. The disorder leads to complete paralysis and respiratory failure between 3 to 5 years after symptom onset [1]. Currently there is no effective drug available for ALS; only one approved drug, riluzole, shows moderate effects on survival but not on muscle strength [2, 3].

Neuroinflammation, consisting of activated microglia and astrocytes as well as infiltrating T cells, is a common feature in the pathology of ALS [4–7]. Pro- and anti-inflammatory cytokines and chemokines are increased in serum samples of ALS patients as well as in an ALS mouse model [6, 8]. Interleukin–1 (IL–1) is a cytokine that plays a central role in regulating inflammation. IL-1ß, a protein in the IL–1 family, is synthesized as an inactive proform that is proteolytically activated by caspase–1 in response to various “danger” signals by cytosolic protein complexes called inflammasomes [9]. Activated caspase–1 is present in cerebral spinal fluid and spinal cord sections of ALS patients and in a mouse model of ALS [10]. Interestingly, caspase–1 or IL-1ß deficiencies, as well as blocking the IL-1-receptor prolonged the survival but did not affect the onset of the disease in a mouse model [11, 12]. These data suggest that caspase–1 activation contributes to ALS pathogenesis.

These preclinical studies prompted us to assess the safety and tolerability of Anakinra (ANA), a recombinant human interleukin-1-receptor antagonist, in ALS patients. ANA has been approved to treat rheumatoid arthritis [13]. We decided to treat ALS patients with dominant or exclusive lower motor neuron degeneration (LMND) with the rational that inflammation at peripheral nerve fibres might be more accessible to ANA [14–16] although it was shown to reach effective concentrations in the CNS [17, 18, 19, 20].

## Methods

### Study design and participants

Starting in February 2011, we screened patients with LMND and ALS patients diagnosed according to the revised El Escorial criteria [21] for LMND variants of ALS. All patients underwent electrophysiology during the diagnosis to rule out inflammatory disorders and to confirm a degenerative disease. The study was designed as a mono-center, open-label, single-arm pilot study and was performed at the ALS outpatient clinic of the Charité–University Hospital in Berlin, Germany. Inclusion criteria were a stable dose of 100 mg riluzole per day for at least 90 days before initiation, a forced vital capacity greater than 50%, disease duration between 6 and 48 months and the ability to provide informed consent. Women of childbearing age were included if they were not breastfeeding, had a negative pregnancy test and agreed to use birth control throughout the trial. Patients with current or recurrent infections, significant cardiac conduction abnormality, hepatic, renal or haematological parameters outside the reference range or medication with TNF inhibitors were excluded (S1 Table).

### Standard protocol approvals, registrations, and patient consents

The ethics review board of the State of Berlin approved this study. The data protection officer of the Charité consented to the online self-assessment of adverse events and disease progression using the Internet platform www.ALShome.de. All patients provided written informed consent and a data safety and monitoring board supervised the study. This trial’s identifier at ClinicalTrials.gov is NCT01277315.

### Genetic analysis

Patients were genotyped for the two most frequently mutated ALS genes (*C9orf72* and *SOD1*), which are found to carry mutations in approximately 80% of familial ALS cases in Germany. Genotyping was performed by Sanger sequencing of all exons (*SOD1*) or the sequential combination of fragment length analysis, triple PCR and Southern blotting (*C9orf72*) as described recently [22].

### Endpoints

The primary endpoints of the study were safety and tolerability of ANA in combination with riluzole in patients with ALS. Safety was measured by determining adverse and serious adverse events throughout the one-year treatment period. Tolerability was determined by compliance (number of ANA dosages provided/number of ANA dosages planned and IL-1RA serum levels over the 52-weeks treatment period). Secondary endpoints were the number of patients undergoing tracheotomy, invasive assisted ventilation or percutaneous endoscopic gastrostomy, the number of patients completing the study and the clinical effectiveness as measured by manual muscle test, revised ALS functional rating scale (ALSFRSr) [23] and forced expiratory vital capacity. Serum cytokines and inflammatory markers were included as exploratory endpoints.

### Procedures

Patients were trained and instructed to inject the daily dose of 100 mg ANA throughout the study. Patients attended the outpatient clinic at 4, 8, 12, 24, 36 and 52 weeks to undergo a complete neurological examination. Haematological parameters were determined at the laboratories of Labor Berlin, cytokines and chemokines, including IL-1RA, were measured in frozen serum samples by ELISA or bead-based cytoplex assays by Labor Berlin and the MPIIB.

### Data analysis

We characterized the populations using absolute and relative frequencies in categorical variables. We used mean and standard deviation or median and quartiles for continuous or ordinal variables. Depending on the scale and distribution, we performed Chi-square tests, t-test or Mann-Whitney-U test to test for independent variables. We used the paired t-test or Wilcoxon signed-rank test to assess dependent continuous variables or dependent values with ordinal scale, such as continuous or ordinal variables at two time points in one group. Due to the exploratory nature of the study, the calculated p-values were considered exploratory and non-confirmatory. P-values below 0.05 were regarded statistically significant. Due to some variability of patient’s visit times, ALSFRSr values obtained in between the defined visit time points of 4, 8, 12, 24, 36 and 56 weeks were averaged using the midpoint between two time points as cutoff. Missing ALSFRSr values were multiply imputed (m = 20) using chained equations. Results were computed in each imputed dataset and were pooled afterwards. We used the commercially available software SAS 9.3 and the free software R 3.1.1 including the package mice to perform all analyses.

### Historical control group

Patients at the Charité ALS outpatient department have excess to an online assessment platform to improve care [24]. The data base includes 2500 patients and we selected those that fulfilled the inclusion and exclusion criteria of the study group as described at the beginning of the Methods section. Of the 2500 patients, 866 individuals fulfilled the criteria for enrolment in our study and were not included in other interventional trials. We only included patients that entered self-assessments for at least one year and with a similar regularity than the study group. To allow a fair comparison to the small number of patients in our cohort, we randomly selected 47 patients to form the HCG [25]. There were more women in the HCG than in the study cohort (39% vs 6%) (S2 Table).

## Results

**Fig 1:**
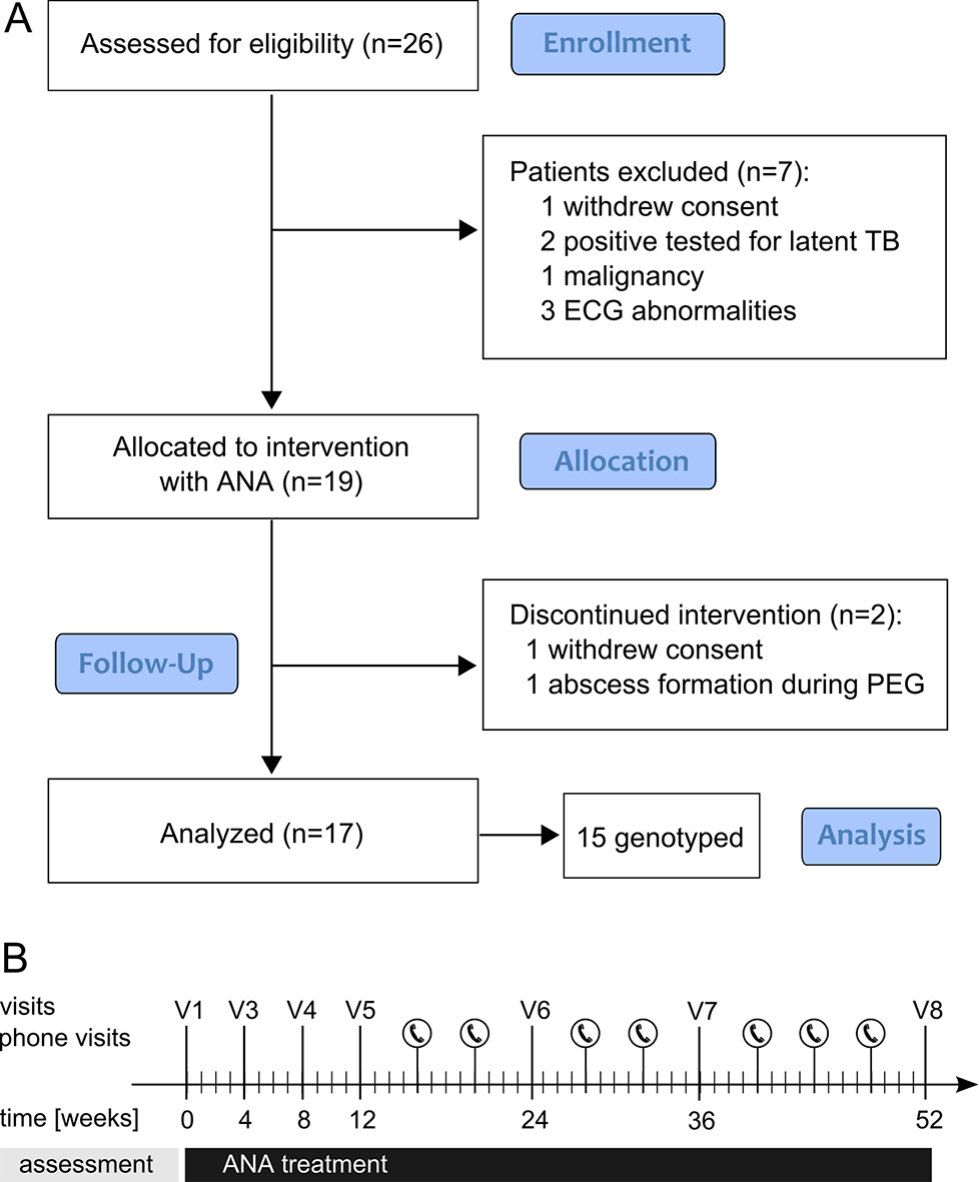
Study design.

We assessed 26 and enrolled 19 patients (17 men/2 women) that fulfilled our inclusion criteria (Fig 1). Patients were 45 to 72 years old (mean age 57.9 y (SD 7.6) at baseline) and diagnosed with exclusive LMND or laboratory-supported probable, or definite ALS according to the revised El Escorial criteria (Table 1) [21] with a dominant LMND. The patients were enrolled at an early stage with a mean ALSFRSr of 40.7 (SD 3.9) and a moderate disease progression rate at baseline (mean delta ALSFRSr = 0.35, S2 Table). The disease duration presented a mean of 22.4 months (SD 8.6). None of the patients had a clinically apparent hypoventilation syndrome at baseline.

**Table 1:** Baseline characteristics of all the patients treated with ANA. Abbreviations: ALSFRSr = amyotrophic lateral sclerosis functional rating scale–revised. LMND = lower motor neuron disease. VC = respiratory viral capacity. UE = upper extremity. LE = lower extremity. n.d. = not determined.

Patient	Gender	Age at baseline (years)	Disease duration at baseline (months)	Family history of ALS	*C9orf72* repeats	Exclusive LMND	Disease onset	ALSFRSr at baseline	VC at baseline	VC final
1	Male	59	36	No	1700	No	LE	38	103	98
2	Male	59	25	No	-	Yes	UE	42	91	88
3	Male	51	21	No	-	Yes	UE	44	83	70
4	Male	52	11	No	-	No	LE	38	58	42
5	Male	51	26	No	-	Yes	LE	42	72	49
6	Male	72	15	No	-	Yes	LE	46	101	94
7	Male	69	26	No	-	No	UE	43	89	84
8	Male	49	24	No	-	Yes	LE	38	104	86
9	Male	60	18	No	2400	No	UE	43	82	24
10	Male	59	15	No	2000	Yes	UE	45	76	52
11	Male	45	36	No	-	No	LE	37	79	66
12	Male	53	18	No	n.d.	Yes	LE	38	83	71
13	Male	59	10	No	n.d.	Yes	LE	38	86	67
14	Male	66	16	No	-	No	UE	47	85	68
15	Female	60	39	No	2300	Yes	LE	42	92	111
16	Male	63	27	No	-	No	UE	32	73	47
17	Male	49	18	No	-	Yes	UE	39	66	51
Mean (SD)		57.4 (7.5)	22.4 (8.6)					40.7 (3.9)	83.7 (12.6)	68.7 (22.9)

Two patients dropped out of the trial prematurely: one patient withdrew consent after 4 weeks without serious adverse events (SAE). A second patient suffered from peritoneal abscess formation after ALS-related percutaneous endoscopic gastrostomy (PEG). ANA treatment was stopped although this SAE was unlikely to be caused by this drug.

Seventeen patients completed the 52 weeks treatment with full compliance (Table 1). This was shown by the expected drug demand by the patients and, more reliably, by the consistently elevated ANA serum levels (S1 Fig).

The patients documented adverse effects weekly online (in total 635 reports) with the verbal rating scale (VRS) for headache and a Likert scale (0–3) for erythema, swelling, pain and bleeding at the site of injection. As reported [26], reactions at the site of injection were the most common adverse effects (Table 2) with a rapid decrease in intensity and frequency during the course of the trial. Mild headaches were frequently reported (47% of all patients reported headache at least once). Seven patients had respiratory tract infections, presumably of viral origin, which cleared without antibiotic therapy. There were three reports of diarrhoea and one case of gastritis that resolved spontaneously. Importantly, we did not observe any new safety concerns using Anakinra in ALS patients.

**Table 2:** Safety profile.

	Patients with event	Total number of events (% of reports)	Degree of severity		
Serious adverse events (SAE)			mild	moderate	severe
Abscess formation during PEG	1 (6%)*	1 (0.2%)	0	0	1
**Adverse events (AE)**					
Respiratory tract infection	7 (41%)	11 (1.7%)	2	9	0
Bleeding	12 (70%)	155 (24.4%)	155	0	0
Swelling	12 (70%)	118 (18.6%)	116	2	0
Redness	12 (70%)	81 (12.7%)	71	7	3
Pain	9 (53%)	117 (18.4%)	102	15	0
Headache	8 (47%)	79 (12.4%)	78	1	0
Pain (back/body/shoulder)	3 (18%)	3 (0.5%)	3	0	0
Diarrhoea	2 (12%)	3 (0.5%)	1	2	0
Gastritis	1 (6%)	1 (0.2%)	1	0	0

* Intention-to-treat population; included 18 patients. The patient with abscess formation dropped out from the study. Data are n (%). PEG = percutaneous endoscopic gastrostomy.

Most of the preclinical data rely on ALS caused by point mutations in the *SOD1* gene. However, the majority of ALS cases are sporadic and of unknown etiology with no obvious genetic association. This heterogeneity might hamper the development of successful therapies. Therefore, we wanted to determine whether there was a correlation between the response to ANA and a specific genetic phenotype in our cohort. Fifteen of the patients provided written consent to genotype mutations in *SOD1* and hexanucleotide expansions in *C9orf72*. We did not identify any missense mutations in *SOD1* but, despite the lack of a family history of ALS [27], found four patients with a Southern blot confirmed hexanucleotide repeat expansions of 1700 or more repeats in *C9orf72*. This is an unexpectedly high representation of 24% in the genotyped subpopulation. The patients with *C9orf72* expansions did not appear clinically distinct from the rest of the cohort. We did not observe any difference in disease progression or response to ANA based on the patient´s genotype. None of the patients in the cohort had a clinically apparent type of dementia.

Our study was designed as an exploratory single-arm trial with the primary outcomes safety and tolerability; however, to determine whether ANA treatment had an effect on disease progression, we generated a historical control group (HCG). The 47 individuals of the standard of care HCG showed similar ALSFRSr, delta ALSFRSr, age and disease duration at baseline compared to the study cohort (S2 Table) except that more women were in the HCG than in the study cohort (39% vs. 6%). In this cohort we see no difference between men and women regarding disease progression, duration or age at onset.

**Fig 2:**
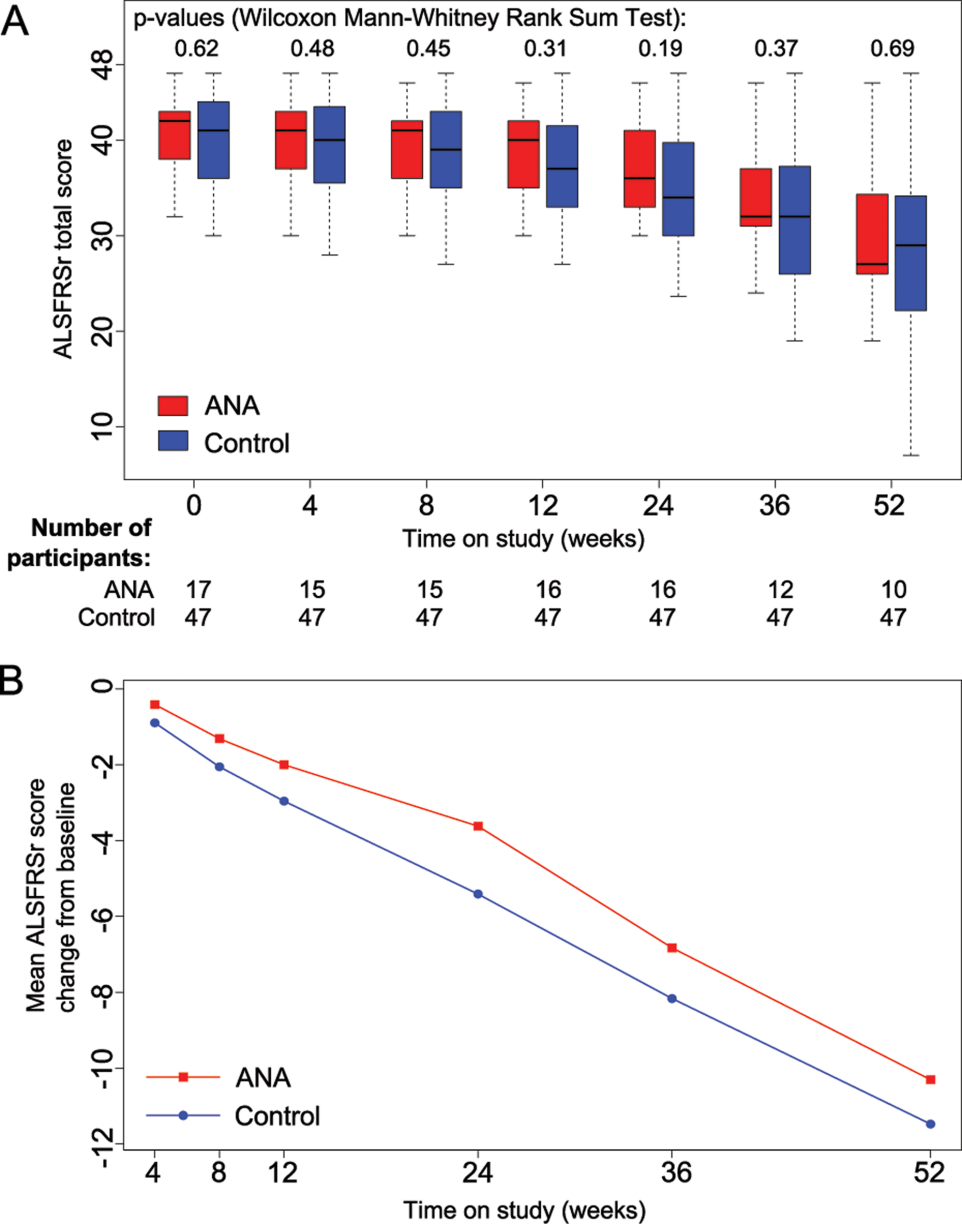
ALS disease progression. Absolute values (A) and changes from baseline (B) in ALSFRSr total score during the 12 months study period comparing the group of patients treated with ANA and the historical control.

**Fig 3:**
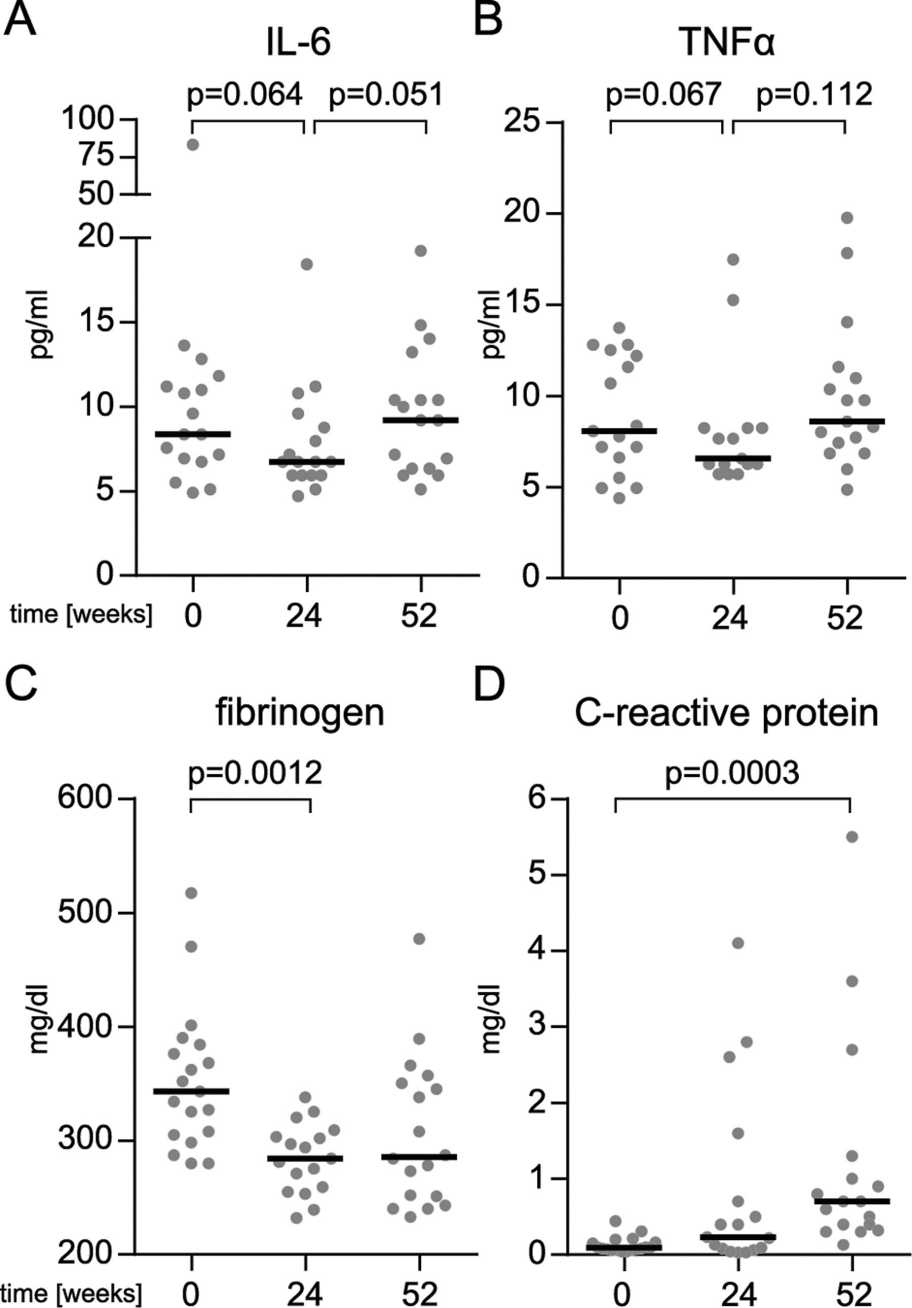
Inflammatory serum parameters. Serum concentrations of the inflammatory cytokines IL–6 (A) and TNF (B) as well as the and hepatic acute phase proteins fibrinogen (C) and C-reactive protein (D) in all the patients included in the study.

The ALSFRSr dropped by 10.76 points (SD 7.1) in the patients treated with ANA over the 52-weeks treatment period (Fig 2). The study cohort showed no overall difference in disease progression compared to the historical control (first 24 weeks: p = 0.19; 52 weeks p = 0.69; ANCOVA analysis for group p = 0.164) (Fig 2). Inflammation is reported to correlate with disease progression [28]; indeed, we observed a relative reduction in the cytokines IL–6 and TNF 24 weeks after study onset (Fig 3), without reaching statistical significance, probably due to the small group size. In addition we measured several serum markers of systemic inflammation [28]. Notably, serum fibrinogen concentrations decreased from 342.3 (SD 52.6) to 306.2 mg/dl (SD 89.8) after 4 weeks of ANA treatment. This reduction persisted throughout the trial (Fig 3; p < 0.01).

In contrast, several cytokines increased between weeks 24 and 52, suggesting secondary ineffectiveness of the treatment in the latter half of the study period (Fig 3C). C-reactive protein (CRP) levels were low at baseline and increased significantly towards the end of the study period (Fig 3D) [28]. The average expiratory vital capacity decreased from 83.7% (SD 12.6) at baseline to 68.7% (SD 22.9) at the end of the trial.

Remarkably, 16 out of 17 patients (94%) generated IgG antibodies against ANA as measured by ELISA (S2 Fig). We confirmed by SDS gel electrophoresis and mass spectrometry that commercially available ANA, which we used both for the patients and for the ELISA, was pure (S2B Fig). Most patients developed anti-ANA antibodies after only four weeks of treatment and the titers remained high throughout the study (S2C Fig).

## Discussion

Inflammation has been described both in patients and animal models of ALS, but its role in ALS pathogenesis remains incompletely understood [6]. Based on preclinical studies, we proposed that the activation of caspase–1 and the production of IL–1 contributed to disease progression [11, 12].

Here we report that ANA in combination with riluzole in ALS patients with LMND is safe and well tolerated. This is consistent with previous reports from patients with rheumatoid arthritis [29]. In ALS patients this observation is of special interest, since they are prone to respiratory tract infections due to hypoventilation and aspiration [1, 30, 31]. We observed no new safety concerns for the use of ANA in this patient cohort. Our data show that ANA is well tolerated and can be considered safe in ALS patients.

This pilot study was not designed to detect significant differences in efficacy nor was it sufficiently powered. However, we observed serum fibrinogen, which is elevated in ALS patients [28], to be reduced upon ANA treatment. However, reduction of inflammatory cytokines like IL–6 and TNF did not reach statistical significance after six months of treatment.

Since most of the patients generated anti-ANA antibodies, it is possible that they blocked ANA and abrogated clinical effectiveness. Indeed, neutrophil numbers (S3 Fig) and inflammatory cytokine levels (especially IL–6 and TNF) increased between weeks 24 and 52, suggesting that ANA was no longer effective. The incidence of anti-ANA antibodies here is higher than reported previously [32] [33]. We do not know whether the incidence of antibodies against ANA is underreported in the literature. It was been reported that only a subfraction of anti-ANA antibodies have blocking effects in cell culture, but we assume that any specific antibody titer effects either pharmacokinetics or pharmakodynamics *in vivo*, especially related to permeation of the blood-brain-barrier.

We propose that targeting IL–1 induced inflammation leads to a reduction in IL–1 mediated inflammation. IL–1 is produced mainly by monocytes and macrophages as well as microglia. It drives the production of IL–6 and exacerbates inflammation. The local production of IL–1 may affect peripheral nerves. We recruited LMND patients based on the hypothesis that they particularly benefit from bioavailable ANA at the peripheral part of lower motor neurons. Nevertheless, ANA might achieve effective concentrations in the CNS by passive transport across the blood-brain-barrier under steady state and disease conditions [14, 15].

Previous attempts to diminish inflammation with cyclooxygenase–2 (COX) inhibitors [34, 35], minocycline [36, 37], sodium chlorite [38], interferon-beta [39] and thalidomide [40, 41] did not show beneficial effects on ALS progression. It is important to note that this trial included only patients with LMND and also that we started the treatment relatively early after disease onset (median ALSFRSr of 41.6; SD 3.1) [42]. This also means that larger and more heterogeneous patient groups with ALS need to be tested before recommending anti-IL1 for broader groups of patients. The pilot study was subject to the inherent limitations of (1) a small number of patients precluding robust statistical analysis of the data, (2) analysis of inflammatory parameters in serum but not in CSF and (3) lacking a double-blinded placebo control group and using a historical control group instead.

In summary, our results suggest that IL–1 may serve as a pharmacological target for ALS-related inflammation; however, it remains an open question whether this leads to slower disease progression. Given the proven safety and tolerability of ANA in ALS, follow-up trials sufficiently powered to determine efficacy are justified. The generation of anti-ANA antibodies is a challenging problem; alternatively higher ANA dosage or a different route of application (e.g. intravenous or intrathecal) (14–16) could be useful. Peripheral inflammatory mediators, like cytokines, might provide the basis for futility design studies to assess anti-inflammatory drug candidates in ALS.

## Supporting Information

S1 Fig Interleukin-1RA serum concentration in all the patients that participated in the study. Plotted are individual values, median. High values are capped at 1,3x10^5^ pg/ml.
(TIF)

S2 Fig Patients treated with ANA develop anti-ANA antibodies. (A) Coomassie-blue stain of SDS-PAGE of commercially available ANA demonstrating the purity of the material. The identity of the protein was confirmed by mass spectrometry. (B) Anti-ANA titrations for each patient (measured colorimetrically by ELISA) before and after treatment with ANA. On the right shows the patient number sera that are also tested in “C” (C) Commercially available ANA was resolved by SDS-PAGE, transferred to a membrane and blotted with patients’ sera. None of the sera recognized ANA before treatment and three of the patients (11, 14 and 5) developed antibodies specific to ANA. (D) ELISA titrations for each of the patients during the study. The titers of anti-ANA antibodies increased during the treatment (except for patient 4).
(TIF)

S3 Fig Blood cell counts of all study patients treated with ANA. Counts for neutrophils (A), erythrocytes (B), thrombocytes (C) and eosinophils (D) for all the patients included in the study treated with ANA. Plotted are individual counts, median.
(TIF)

S1 Table Inclusion and exclusion criteria for eligibility in this study. * According to the revised El Escorial Criteria.
(DOC)

S2 Table Baseline characteristics of the patients included in the historical control and the treated cohort. Data are mean (SD) or n (%). ALSFRSr = Amyotrophic lateral sclerosis functional rating scale–revised. d/eLMND = dominant/exclusive lower motor neuron degeneration.
(DOC)

S1 File Original study protocol (German). (PDF)

S2 File Translated study protocol (English). (PDF)

S3 File TREND checklist. (PDF)
