# Supplementary figures and images for: Interleukin-1 Antagonist Anakinra in Amyotrophic Lateral Sclerosis—A Pilot Study
Source: PLoS One. 2015 Oct 7;10(10):e0139684. doi: 10.1371/journal.pone.0139684 (PMC4596620; doi:10.1371/journal.pone.0139684)

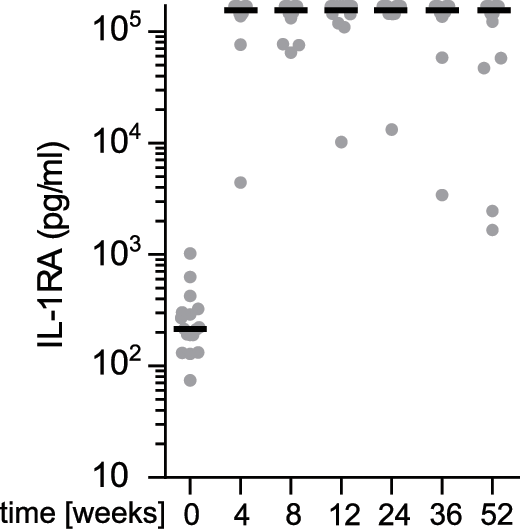

Supplement: S1 Fig — Plotted are individual values, median. High values are capped at 1,3x10^5^ pg/ml. (TIF) [file pone.0139684.s001.tif]

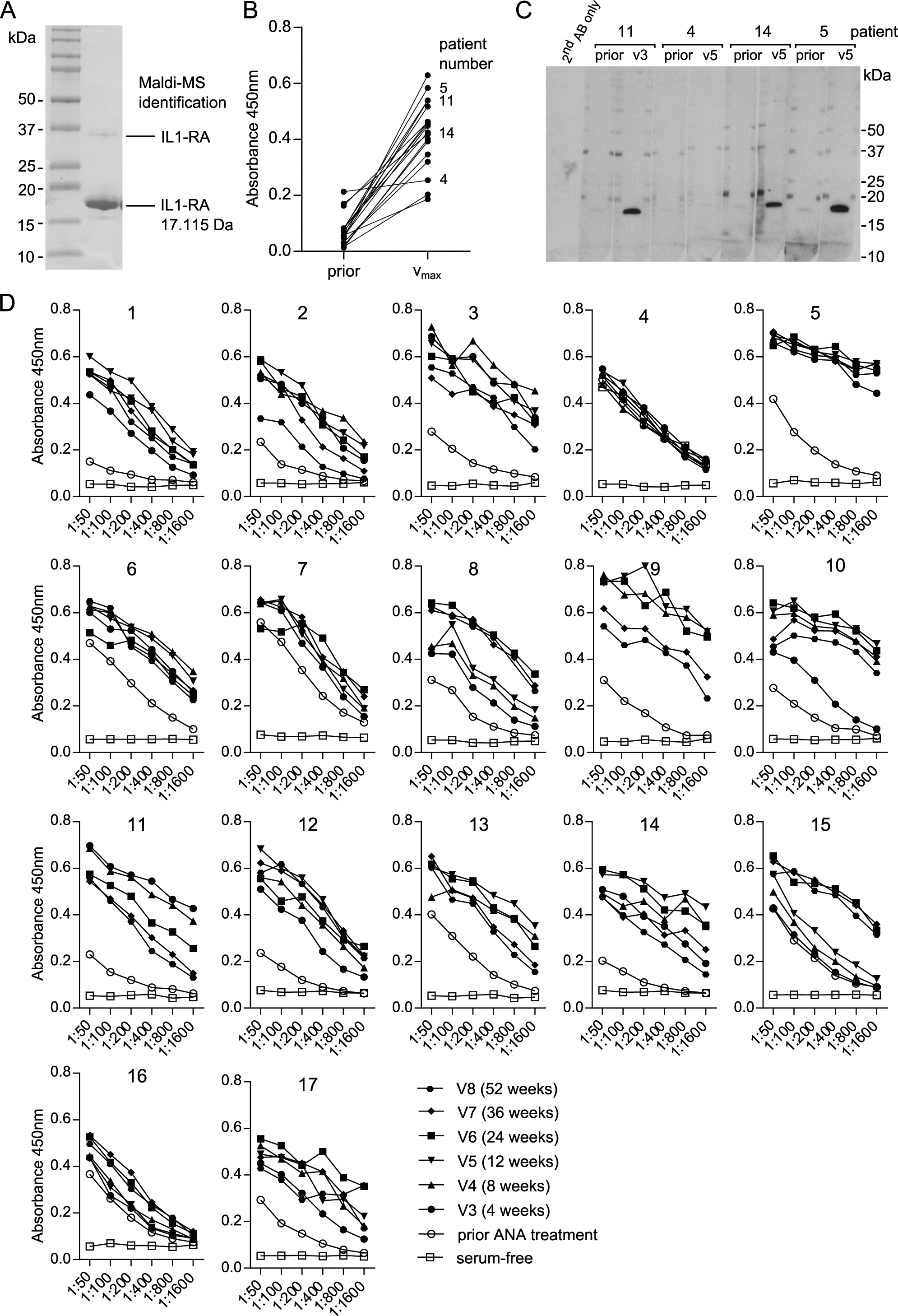

Supplement: S2 Fig — (A) Coomassie-blue stain of SDS-PAGE of commercially available ANA demonstrating the purity of the material. The identity of the protein was confirmed by mass spectrometry. (B) Anti-ANA titrations for each patient (measured colorimetrically by ELISA) before and after treatment with ANA. On the right shows the patient number sera that are also tested in “C” (C) Commercially available ANA was resolved by SDS-PAGE, transferred to a membrane and blotted with patients’ sera. None of the sera recognized ANA before treatment and three of the patients (11, 14 and 5) developed antibodies specific to ANA. (D) ELISA titrations for each of the patients during the study. The titers of anti-ANA antibodies increased during the treatment (except for patient 4). (TIF) [file pone.0139684.s002.tif]

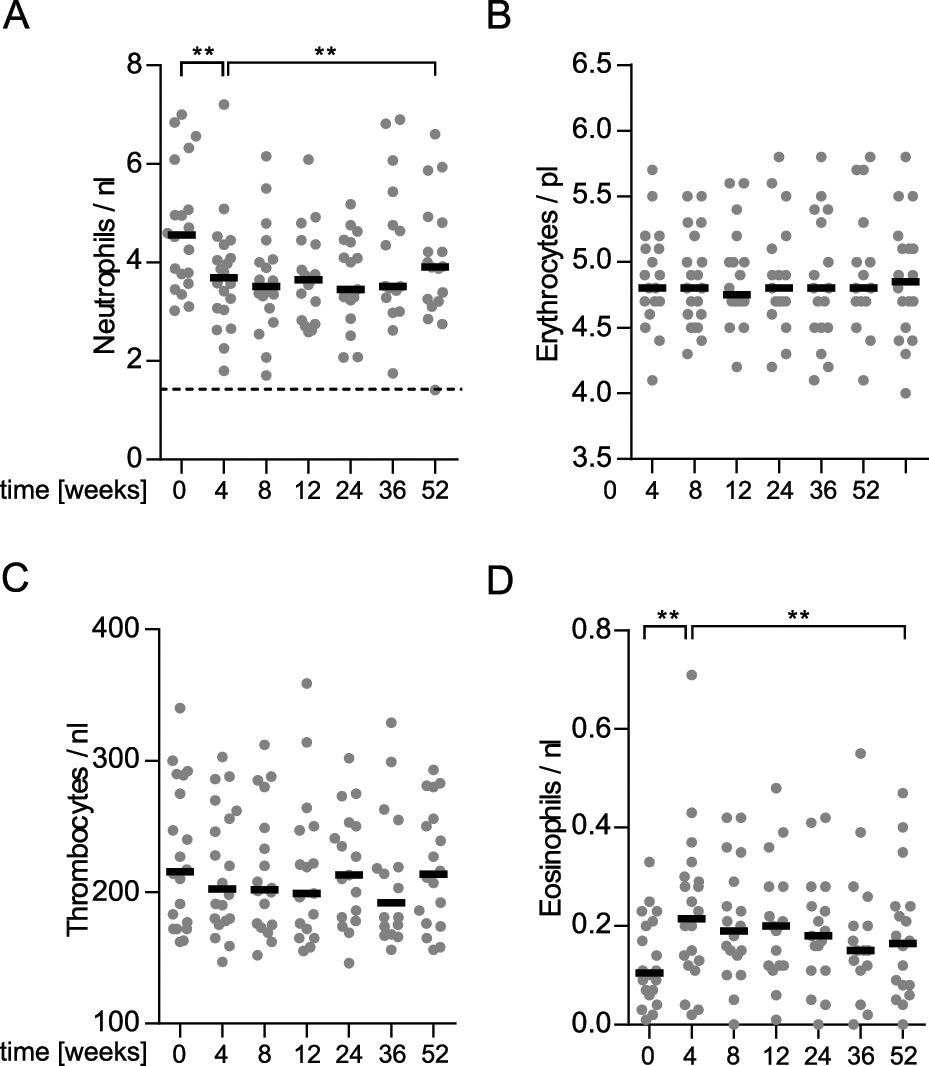

Supplement: S3 Fig — Counts for neutrophils (A), erythrocytes (B), thrombocytes (C) and eosinophils (D) for all the patients included in the study treated with ANA. Plotted are individual counts, median. (TIF) [file pone.0139684.s003.tif]
