# Supplementary material for: Interleukin-1 Antagonist Anakinra in Amyotrophic Lateral Sclerosis—A Pilot Study
Source: PLoS One. 2015 Oct 7;10(10):e0139684. doi: 10.1371/journal.pone.0139684 (PMC4596620; doi:10.1371/journal.pone.0139684)
Supplement: S1 Table — * According to the revised El Escorial Criteria. (DOC) [file pone.0139684.s004.doc]

| **Inclusion criteria** | **Exclusion criteria** |
| --- | --- |
| Clinical diagnosis of laboratory-supported probable, probable or definite ALS* with dominant affection of the second motor neuron or progressive muscular atrophy (PMA) | No known hyper-sensitivity to any substance used in the trial (riluzol, anakinra, additives) |
| Age between 18 and 80 years | Abnormal myocardial conductance disturbance |
| Sporadic or familial ALS | Pregnancy or breast-feeding |
| Symptom onset between 4 years and 6 months before study medication | Hypoventilation with vital capacity <50% or assisted ventilation or tracheotomy |
| Treatment with Riluzole 100 mg/ day > 1 month | Serum and hematological parameters outside reference range |
| Informed consent | Malignancies |
| Access to internet | Renal insufficiency (renal clearance <30 ml/min) |
| No participation in other trials | Recurrent infections |
|  | Monoclonal gammopathy of unknown significance |
|  | Chronic infections including HIV, hepatitis b and c |
|  | Severe psychiatric disorders |
|  | Dementia or other caveat against informed consent |
|  | Epilepsy |
|  | Expected incompliance |
|  | Medication with TNF-antagonists |

**S1 Table: Inclusion and exclusion criteria for eligibility in this study.**

* According to the revised El Escorial Criteria.
