# Supplementary material for: Interleukin-1 Antagonist Anakinra in Amyotrophic Lateral Sclerosis—A Pilot Study
Source: PLoS One. 2015 Oct 7;10(10):e0139684. doi: 10.1371/journal.pone.0139684 (PMC4596620; doi:10.1371/journal.pone.0139684)
Supplement: S2 Table — Data are mean (SD) or n (%). ALSFRSr = Amyotrophic lateral sclerosis functional rating scale–revised. d/eLMND = dominant/exclusive lower motor neuron degeneration. (DOC) [file pone.0139684.s005.doc]

|  | **ANA treatment** | **Historical control** |
| --- | --- | --- |
| **Number of patients** | 17 | 47 |
| **Gender ratio male/female (%)** | 16/1 (94%/6%) | 29/18 (62%/39%) |
| **ALSFRSr at baseline** | 40.7 (3.9) | 40.1 (4.9) |
| **Mean delta ALSFRSr at baseline** | 0.35 (0.24) | 0.41 (0.25) |
| **Disease duration at baseline, months** | 22.4 (8.6) | 24.1 (16.5) |
| **dLMND/eLMND (%)** | 10/7 (59%/41%) | 31/16 (66%/34%) |
| **Age at disease onset, years** | 55.9 (7.8) | 58.5 (9.3) |
| **Age at baseline, years** | 57.4 (7.5) | 60.5 (9.2) |
| **Concomitant riluzole medication** | 100% | 100% |

**S2 Table: Baseline characteristics of the patients included in the historical control and the treated cohort.** Data are mean (SD) or n (%). ALSFRSr = Amyotrophic lateral sclerosis functional rating scale – revised.

d/eLMND = dominant/exclusive lower motor neuron degeneration.
