# Supplementary material for: Interleukin-1 Antagonist Anakinra in Amyotrophic Lateral Sclerosis—A Pilot Study
Source: PLoS One. 2015 Oct 7;10(10):e0139684. doi: 10.1371/journal.pone.0139684 (PMC4596620; doi:10.1371/journal.pone.0139684)
Supplement: S1 File — (PDF) [file pone.0139684.s006.pdf]

Studientitel

Offene Verträglichkeitsstudie zur Evaluierung einer subkutanen Injektionslösung von 100 mg Anakinra in Kombination mit Riluzol bei Patienten mit Amyotropher Lateralsklerose (ALS).

Kurztitel der Studie: Anakinra-Verträglichkeitsstudie bei der ALS
Studiencode: ANA-ALS01

Kurzbezeichnung / Acronym: Anakinra bei der ALS
EudraCT Nummer: 2010-019218-26

<u>Prüfplan</u>

Version 1.4
Datum: 03.03.2011

Sponsor der klinischen Prüfung:

Charité Universitätsmedizin Berlin
Charitéplatz 1
10117 Berlin

Vertreter des Sponsors und Hauptprüfer:

Prof. Dr. Thomas Meyer, Charité – Universitätsmedizin Berlin, Campus Virchow-Klinikum, Neurologische Klinik und Poliklinik, Augustenburger Platz 1, 13353 Berlin

---

Die nachfolgenden Personen stimmen den Inhalten dieses Protokolls durch ihre Unterschrift zu und bestätigen, dass ihnen die ICH-GCP-Guidelines, die Anforderungen des AMG und der GCP-Verordnung bekannt sind und dass die klinische Prüfung nach diesen Vorschriften durchgeführt wird.

**Vertreter des Sponsors**	Prof. Dr. Thomas Meyer	Berlin, 03.03.2011<br>Ort, Datum
**Hauptprüfer**	Prof. Dr. Thomas Meyer	Berlin, 03.03.2011<br>Ort, Datum

\- Vertraulich -

Die Informationen in diesem Protokoll sind streng vertraulich zu behandeln. Sie dienen nur zur Information des Prüfarztes, seiner Mitarbeiter, der Ethik-Kommission, der Bundesoberbehörde, des KKS und zur Patientenaufklärung.

1. Verantwortlichkeiten

Sponsor:	Charité – Universitätsmedizin Berlin<br>Charitéplatz 1, 10117 Berlin
Vertreter des Sponsors:	Prof. Dr. Thomas Meyer<br>Charité – Universitätsmedizin Berlin<br>Campus Virchow-Klinikum<br>Neurologische Klinik und Poliklinik<br>Augustenburger Platz 1, 13353 Berlin<br>Tel.: 030.450-660032; 030.450-560132 (Sekretariat)<br>Fax: 030.450-560907<br>E-Mail: thomas.meyer@charite.de
Anzahl der Zentren:	1
Hauptprüfer im Studienzentrum:	Prof. Dr. Thomas Meyer
Betreuende Prüfärzte der Charité:	Prof. Dr. Christoph Münch, Facharzt für Neurologie<br>Dr. Nadja Borisow, Assistenzärztin<br>Teresa Holm, Assistenzärztin<br>Dr. Peter Linke, Assistenzarzt<br>André Maier, Assistenzarzt
Weitere beteiligte Institutionen:	Max Planck Institut für Infektionsbiologie<br>Prof. Dr. Arturo Zychlinsky<br>Charitéplatz 1, 10117 Berlin<br><br>Charité - Universitätsmedizin Berlin<br>Zentralapotheke<br>Dr. Susan Bischoff<br>Augustenburger Platz 1, 13353 Berlin
Monitoring, Biometrie, Datenmanagement:	Koordinierungszentrum für Klinische Studien,<br>Augustenburger Platz 1, 13353 Berlin
Pharmakovigilanz:	Koordinierungszentrum für Klinische Studien,<br>Augustenburger Platz 1, 13353 Berlin
Referenzinstitutionen mit Nennung der Verantwortlichen	Prof. Dr. Eckart Köttgen<br>Institut für Laboratoriumsmedizin und Pathobiochemie<br>Campus Virchow-Klinikum<br>Augustenburger Platz 1<br>13353 Berlin<br><br>Prof. Dr. Hans-Dieter Volk<br>Institut für medizinische Immunologie<br>Campus Charité Mitte<br>Charitéplatz 1<br>10117 Berlin

2. Inhaltsverzeichnis

1. Verantwortlichkeiten 02
2. Inhaltsverzeichnis 03
3. Synopsis 05
4. Ablaufschema 21

5.1 Einleitung 25
5.2. Fragestellung und Begründung 31
5.3. Risiko-Nutzen-Abwägung 32

6. Ziele der klinischen Prüfung 33
6.1. Hauptzielkriterium 33
6.2. Nebenzielkriterien 33
6.3. Studiendesign 33
6.4. Zeitplan 34

7. Patienten 34
7.1. Einschlusskriterien 34
7.2. Ausschlusskriterien 34

8. Prüfmaterialien 35
8.1. Studienmedikation: 35
8.1.1. Prüfmedikation: 35
8.1.1.1. Beschreibung der Prüfmedikation 35
8.1.1.2. Verzeichnis der Nebenwirkungen und Wechselwirkungen 36
8.1.2. Placebo und Vergleichsmedikation 37
8.2. Behandlungsschema 37
8.2.1. Anweisung für die Applikation 37
8.2.2. Anweisung für Dosisanpassung: 38
8.2.2.1. Notfallmaßnahmen....38
8.3. Begleitmedikation....38
8.3.1. Besondere Kennzeichnung....38
8.4. Lagerung, Aus- und Rückgabe sowie Dokumentation der Studienmedikation 38
8.5. Compliance 39
8.6. Anforderungen an Prüfzentren und Prüfärzte 39
8.7. Aufnahme und Registrierung 39
8.7.1. Rekrutierungs-Logbuch 39
8.7.2. Registrierung 39
8.8. Studienablauf 40
8.8.1. Klinische Untersuchungen und Statuserhebungen 40
8.8.1.1. Eingangs- und Einschlussuntersuchung 40
8.8.1.2. Verlaufsuntersuchungen 42
8.8.1.3. Abschlussuntersuchung 43
8.8.1.4. Laboruntersuchung 43
8.9. Dauer der Studienteilnahme für den einzelnen Patienten 43
8.10. Vorzeitiger Studienabbruch eines einzelnen Patienten 43
8.11. Vorzeitiger Abbruch der gesamten klinischen Prüfung 44

9. Unerwünschte Ereignisse 44
9.1. Definition unerwünschter Ereignisse (AE) 44
9.2. Definition schwerwiegender unerwünschter Ereignisse (SAE) 45
9.3. Beurteilung der Intensität von unerwünschten Ereignissen: 45
9.4. Beurteilung des Kausalzusammenhangs 46

9.5. Dokumentation unerwünschter Ereignisse 46
9.6. Meldung schwerwiegender und/oder unerwünschter Ereignisse 46
9.7. Follow-ups von SAE 47
9.8. Meldung von Verdachtsfällen unerwarteter schwerwiegender Nebenwirkungen (Suspected Unexpected Serious Adverse Reaction, SUSAR) 47
9.9. Schwangerschaft 48
9.10. Ansprechpartner für die Meldung von SAE 48

10. Dokumentation 48
10.1 Dokumentationsbögen (CRF) 48
10.2. Prüfarztordner 48
10.3. Internetportal 49

11. Qualitätsmanagement 49
11.1 Überwachung des Studienablaufs und der Datenqualität 49
11.1.1 Monitoring 49
11.1.2. Audit 49
11.2. Standardisierung und Validierung 49
11.3. Referenzinstitutionen 50

12. Dateneingabe und Datenmanagement 50
12.1. Datenerhebung und Dokumentationsbögen 50
12.2. Datenverarbeitung 51
12.3. Generierung des Pseudonyms 51

13. Statistische Analyse 52
13.1. Fallzahlabschätzung 52
13.2. Statistische Auswertung 52
13.2.1 Hypothesen 52
13.2.2. Definition der Auswertepopulation 52
13.2.3. Auswertung primärer und sekundärer Zielparameter 52
13.2.4. Auswertung der Sicherheit 52
13.2.5. Mögliche Zwischenauswertungen und vorzeitiger Studienabbruch 53
13.2.6. Auswertemethodik 53

14. Berichterstattung 53
14.1. Protokolle 53
14.2. Prüfbericht 53
14.3. Abschlussbericht und Publikation 53

15. Ethische, rechtliche und verwaltungstechnische Aspekte 53
15.1. Rechtliche Voraussetzungen für die Studie 53
15.2. Patienteninformation und Einverständniserklärung 54
15.3. Patientenversicherung 54
15.4. Datenschutz und Schweigepflicht 54
15.5. Aufbewahrung der Daten 54
15.6. Gesetzliche Grundlagen 54
15.7. GCP 55
15.8. Finanzierung der Studie 55
15.9. Einhaltung des Protokolls und der Protokolländerungen 55

16. Literaturverzeichnis 56

17. Anhänge 58

3. Synopsis

Titel der Studie	Offene Verträglichkeitsstudie zur Evaluierung einer subkutanen Injektionslösung von 100 mg Anakinra in Kombination mit Riluzol bei Patienten mit Amyotropher Lateralsklerose (ALS).
Art des Vorhabens	Klinische Prüfung der Phase II nach AMG
Hauptprüfer	Prof. Dr. Thomas Meyer<br>Charité – Universitätsmedizin Berlin<br>Campus Virchow-Klinikum<br>Neurologische Klinik und Poliklinik<br>Augustenburger Platz 1, 13353 Berlin
Hypothese	In einer offenen Verträglichkeitsstudie soll überprüft werden, ob Anakinra (ANA) in einer subkutanen Injektionsdosis von 100 mg ANA in Kombination mit Riluzol eine Verträglichkeit bei Patienten mit ALS aufweist.
Fragestellung aufbauend auf dem wissenschaftlichen Hintergrund	Die ALS ist eine neurodegenerative Erkrankung des Erwachsenenalters, die durch eine selektive Degeneration von motorischen Nervenzellen im Kortex und im Myelon verursacht wird. In Folge der Motoneuronendegeneration entwickeln sich progrediente Paresen der Extremitäten-, Sprech-, Schluck- und Atemmuskulatur. Die ALS führt in einem mittleren Verlauf von 3-5 Jahren zum Tode durch respiratorische Insuffizienz. Riluzol (Sanofi-Synthelabo-Aventis) ist die bisher einzige zugelassene Medikation, die zu einer geringgradigen Lebenszeitverlängerung von 1,5 – 2, 5 Monaten führt. Riluzol ist ein Inhibitor der präsynaptischen Glutamatfreisetzung. Ein weiterer Wirkmechanismus von Riluzol ist die Hemmung der Glutamatfreisetzung aus aktivierter Mikroglia.<br><br>Die Neuroinflammation ist ein wesentlicher pathogenetischer Faktor der Motoneuronendegeneration. Inflammatorische Veränderungen sind bei der sporadischen ALS, der autosomal-dominanten Form der ALS sowie im transgenen SOD1-Mausmodell der ALS nachweisbar. Morphologisch ist eine Akkumulation von aktivierter Mikroglia, Astrozyten und T-Zellen festzustellen. Begleitende biochemische Veränderungen umfassen eine Aktivierung und Freisetzung des proinflammatorischen Zytokins Interleukin (IL)-1. Der Entzündungsmediator IL-1 vermittelt die Rekrutierung und Aktivierung von phagozytischen Zellen und induziert eine Immunantwort. Die Bioaktivität von IL-1 wird physiologisch durch den natürlich vorkommenden, hochselektiven Interleukin-1-Rezeptor-Antagonist (IL-1RN) kontrolliert.<br><br>Auf der Grundlage neuroinflammatorischer Veränderungen bei der ALS besteht eine wissenschaftliche Rationale einer klinischen Studie mit ANA, das über antiinflammatorische pharmakologische Eigenschaften verfügt. ANA ist ein rekombinant hergestellter IL-1RN (r-metHuIL-1RN), der das IL-1 direkt und selektiv hemmt.

	Die primäre Zielstellung der klinischen Studie beinhaltet die Sicherheit und Verträglichkeit von ANA in Kombinationsbehandlung mit Riluzol bei Patienten mit ALS. In einer offenen Verträglichkeitsstudie soll überprüft werden, ob eine subkutane Injektionsdosis von 100 mg ANA toleriert wird. Die Verträglichkeitsstudie ist als Grundlage für eine Phase IIb/III-Wirksamkeitsstudie anzusehen.
Prüfmedikation	Prüfmedikation: ANA (Kineret®) 100 mg Injektionslösung (Injektionszubereitung) in einer Fertigspritze in Kombination mit einer oralen Dosis 100 mg Riluzol.<br>Offene Behandlung mit 100 mg ANA + 100 mg Riluzol.
Vergleichsmedikation	Keine
Studiendesign	Offene Verträglichkeitsstudie zur Evaluierung der Sicherheit und Verträglichkeit von 100 mg ANA für die Dauer von 52 Wochen in Kombination mit Riluzol.
Zeitplan	Patientenrekrutierung 11-2010 bis 04-2011; Dauer der Studie 52 Wochen. Abschluss der klinischen Studie 04-2012.
Gesamtzahl der Patienten	Behandlung von 20 Patienten mit 100 mg ANA in Kombination mit 100 mg Riluzol.<br>Die biometrische Fallzahlbestimmung ergibt 17 auswertbare Patienten, um den Studieneffekt des primären Endpunktes statistisch zu sichern. Das Screening von 30 – 60 Patienten ist erforderlich, um 20 Patienten in die Studie zu rekrutieren.
Gesamtzahl der Zentren	1
Studienpopulation	Patienten mit Amyotropher Lateralsklerose (ALS)
Einschlusskriterien	• Patienten im Alter ≥ 18 Jahre und ≤ 80 Jahre.<br>• Klinische Diagnose einer ALS (revidierte El Escorial-Kriterien) mit dominanter Affektion des 2. motorischen Neurons oder der klinischen ALS-Variante einer Progressiven Muskelatrophie (PMA)<br>• Klinische Zeichen für eine Störung des 2. motorischen Neurons in mindestens einer anatomischen Region außerhalb der Bulbärregion<br>• Sporadische und familiäre ALS<br>• Symptombeginn mit Paresen sechs Monate bis vier Jahre vor Studieneinschluss<br>• Behandlung mit Riluzol 100 mg/Tag mindestens 3 Monate vor Studieneinschluss<br>• Schriftliche Patienteneinwilligung (laut AMG §40 (1) 3b)<br>• Kommunikationsfähigkeit, persönlich oder mit Hilfe einer bevollmächtigten Person<br>• Kommunikationsfähigkeit, verbal, manuell oder mittels elektronischem Kommunikationssystem<br>• Internetzugang, persönlich oder von bevollmächtigter Person<br>• Kühlmöglichkeit für ANA-Fertigspritzen (Kühlschrank) am

	Wohnort<br>• Keine Teilnahme an einer anderen Studie nach dem AMG während der Teilnahme an diesem Behandlungsplan<br>• Negativer Schwangerschaftstest oder mindestens 24 Monate postmenopausal
Ausschlusskriterien	• Diagnose einer ALS mit überwiegender Schädigung des 1. motorischen Neurons ohne klinische Merkmale einer begleitenden Schädigung des 2. motorischen Neurons in mindestens einer anatomischen Region außerhalb der Bulbärregion (klinische Variante einer spastischen ALS)<br>• Diagnose der klinischen ALS-Variante einer Primären Lateralsklerose (PLS)<br>• Patienten mit bekannter Überempfindlichkeit gegenüber ANA, Riluzol oder einem der Hilfsstoffe<br>• Klinisch schwerwiegendes Hypoventilationssyndrom mit einer Vitalkapazität < 50 %<br>• Schwangere oder stillende Frauen<br>• Frauen im gebärfähigen Alter, die keine hocheffektiven Maßnahmen zur Verhütung einer Schwangerschaft anwenden können oder wollen (definiert als Pearl-Index <1)<br>• Klinisch-signifikante EKG-Veränderungen einschließlich einer symptomatischen Bradykardie<br>• Kontinuierliche nicht-invasive Beatmung mit einer ventilationsfreien Zeit < 2 Stunden<br>• Tracheotomie und mechanische Ventilation<br>• Laborparameter außerhalb des Normbereiches, die einer klinisch-signifikanten kardiovaskulären, pulmonologischen, hämatologischen, hepatologischen, metabolischen und renalen Erkrankung entsprechen<br>• Maligne Tumorerkrankungen<br>• Schwere Nierenfunktionsstörung (Creatinine-Clearance < 30 ml/Minute)<br>• Vorgeschichte von rezidivierenden Infektionen oder eine Grunderkrankung, die für Infektionen prädisponiert<br>• Schwere Neutropenie (absolute Neutrophilenzahl < 1,5 x $10^{9/}$l)<br>• Monoklonale Gammopathie unklarer Signifikanz<br>• Infektionserkrankungen einschließlich der Infektion mit Tuberkulose, HIV, Hepatitis B und C<br>• Patienten mit klinisch schwerwiegenden Begleiterkrankungen, einschließlich psychiatrischer Erkrankungen<br>• Demenz und fehlende Einwilligungsfähigkeit<br>• Anamnese einer Epilepsie oder eines Gelegenheitsanfalls<br>• Behandlung mit einer sonstigen Studienmedikation < 3 Monate vor Studieneinschluss<br>• Krankheiten oder Funktionsstörungen, die nach Beurteilung des Prüfarztes eine Teilnahme an dieser Behandlung ausschließen<br>• Mangelnde Kooperationsbereitschaft<br>• Alle Kontraindikationen gegen Prüfmedikation (auch Hilfsstoffe der Arzneiform)<br>• Fehlende Bereitschaft zur Speicherung und Weitergabe pseudonymisierter Krankheitsdaten im Rahmen der

	klinischen Prüfung<br>• Gleichzeitige Anwendung von ANA und Etanercept oder anderen TNF-Antagonisten<br>• Unterbringung in einer Anstalt auf gerichtliche oder behördliche Anordnung (laut AMG §40 (1) 4)
Dokumentationszeitpunkte	Woche 0: Visite 1 (Screening)<br>Woche 1: Visite 2 (Einschluss und Beginn der Medikation)<br>Woche 2: Internetvisite 1<br>Woche 3: Internetvisite 2<br>Woche 4: Kontrollvisite 3 und Internetvisite 3<br>Woche 5: Internetvisite 4<br>Woche 6: Internetvisite 5<br>Woche 7: Internetvisite 6<br>Woche 8: Kontrollvisite 4 und Internetvisite 7<br>Woche 9: Internetvisite 8<br>Woche 10: Internetvisite 9<br>Woche 11: Internetvisite 10<br>Woche 12: Kontrollvisite 5 und Internetvisite 11<br>Woche 13: Internetvisite 12<br>Woche 14: Internetvisite 13<br>Woche 15: Internetvisite 14<br>Woche 16: Telefonvisite 1 und Internetvisite 15<br>Woche 17: Internetvisite 16<br>Woche 18: Internetvisite 17<br>Woche 19: Internetvisite 18<br>Woche 20: Telefonvisite 2 und Internetvisite 19<br>Woche 21: Internetvisite 20<br>Woche 22: Internetvisite 21<br>Woche 23: Internetvisite 22<br>Woche 24: Kontrollvisite 6 und Internetvisite 23<br>Woche 25: Internetvisite 24<br>Woche 26: Internetvisite 25<br>Woche 27: Internetvisite 26<br>Woche 28: Telefonvisite 3 und Internetvisite 27<br>Woche 29: Internetvisite 28<br>Woche 30: Internetvisite 29<br>Woche 31: Internetvisite 30<br>Woche 32: Telefonvisite 4 und Intervisite 31<br>Woche 33: Internetvisite 32<br>Woche 34: Internetvisite 33<br>Woche 35: Internetvisite 34<br>Woche 36: Kontrollvisite 7 und Internetvisite 35<br>Woche 37: Internetvisite 36<br>Woche 38: Internetvisite 37<br>Woche 39: Internetvisite 38<br>Woche 40: Telefonvisite 5 und Internetvisite 39<br>Woche 41: Internetvisite 40<br>Woche 42: Internetvisite 41<br>Woche 43: Internetvisite 42<br>Woche 44: Telefonvisite 6 und Internetvisite 43<br>Woche 45: Internetvisite 44<br>Woche 46: Internetvisite 45<br>Woche 47: Internetvisite 46<br>Woche 48: Telefonvisite 7 und Internetvisite 47

	Woche 49: Internetvisite 48<br>Woche 50: Internetvisite 49<br>Woche 51: Internetvisite 50<br>Woche 52: Visite 8 (Studienabschluss) und Internetvisite 51<br><br>Nach Abschluss der klinischen Prüfung verbleiben die Patienten in kontinuierlicher Behandlung mit ambulanter Vorstellung im Abstand von 3 Monaten.
Zielgrößen	Das primäre Ziel der Studie besteht in der Überprüfung der Sicherheit und Verträglichkeit einer subkutanen Injektion mit ANA in Verbindung mit einer oralen Dosis Riluzol bei ALS-Patienten. Der gewählte Stichprobenumfang ist nicht geeignet, eine Wirksamkeit von ANA in Kombination mit Riluzol bei der ALS zu sichern. Dennoch soll mittels sekundärer Studienendpunkte die Beeinflussung der ALS-Progressionsrate und der Lebensqualität durch die ANA-Behandlung untersucht werden.<br><br>*Primärer Endpunkt:*<br>Eine Untersuchung von Anzahl und Schweregrad<br>• Unerwünschter Ereignisse (UE)<br>• schwerwiegender unerwünschter Ereignisse (SUE), unerwünschter Arzneimittelwirkungen (UAW),<br>• unerwarteter unerwünschter Arzneimittelwirkungen (uUAW)<br>• sowie schwerwiegender unerwünschter Arzneimittelwirkungen (SUAW)<br>• Verdachtsfälle von unerwarteten schwerwiegenden Arzneimittelwirkungen (SUSAR)<br>• pathologischer Laborparameter<br><br>*Sekundäre Endpunkte:*<br>• Die Evaluierung einer Langzeitverträglichkeit und Arzneimittelsicherheit von ANA in Kombination mit Riluzol<br>• Anzahl der Patienten, die eine kontinuierliche nichtinvasive Ventilation oder Tracheostoma-gestützte invasive Ventilation benötigen<br>• Anzahl der Patienten, die eine perkutane endoskopische Gastrostomie (PEG) benötigen<br>• Anzahl der Patienten, die eine Behandlung mit ANA in Kombination mit Riluzol abgeschlossen haben<br>• Die Evaluierung einer klinischen Wirksamkeit von ANA auf die Progressionsrate der Paresen und funktionellen Einschränkungen bei der ALS, durch die vergleichende Untersuchung der intraindividuellen Progressionsrate des revidierten *ALS Functional Rating Scale* (ALS-FRS-R) in einem Studienzeitraum von 52 Wochen im Vergleich zur Progressionsrate vor der Studienteilnahme (Δ ALS-FRS-R).<br>• Die Untersuchung der Wirksamkeit von ANA auf die respiratorische Funktion bei der ALS, die durch eine forcierte Vitalkapazität im Verlauf von 52 Wochen bestimmt wird.<br>• Die Untersuchung der Wirksamkeit von ANA auf die respiratorische Funktion von ALS-Patienten, die eine nichtinva-

	sive Maskenventilation erhalten (Bestimmung der Ventilationszeit pro Tag).<br>• Ermittlung der Überlebenszeit oder des Zeitpunktes einer Tracheotomie durch respiratorische Insuffizienz (ausgenommen elektive Tracheotomie oder Tracheotomie zur Sekretkontrolle.
Sicherheit	**Aufklärung, Einverständnis und Dokumentation:**<br>• Aushändigung von schriftlichem Informationsmaterial an die Patientin/den Patienten über die Anwendung, die Risiken der Anwendung, die Notwendigkeit einer sicheren Kontrazeption und über geeignete Methoden zur sicheren Kontrazeption<br>• Schriftliche Patienteneinwilligung in die Behandlung<br>• Schriftliche Bestätigung der Aufklärung durch den Patienten und Erklärung, dass die Aufklärung verstanden worden ist<br>• Dokumentation von Aufklärung und Einwilligung<br><br>Patientinnen im gebärfähigen Alter, die sich weder einer Hysterektomie unterzogen haben bzw. deren Menopause weniger als 24 Monate zurückliegt, müssen vor Beginn der Behandlung mit der Prüfmedikation einen Schwangerschaftstest zum Ausschluss einer bestehenden Schwangerschaft durchführen.<br><br>Frauen in gebärfähigem Alter müssen außerdem mindestens einen Monat vor dem Behandlungsbeginn mit der Prüfmedikation während des Behandlungszeitraumes eine hoch effektive Methode zur Kontrazeption (definiert als Pearl Index < 1) wie z. B. die Pille, Hormonspirale, Hormonimplantat, Transdermalpflaster, eine Kombination aus zwei Barrieremethoden (Kondom und Diaphragma), eine durchgeführte Sterilisation oder sexuelle Abstinenz anwenden.<br><br>Maßnahmen vor der Behandlung mit der Prüfmedikation bei Frauen im gebärfähigen Alter:<br>• Schwangerschaftstest aus dem Urin vor Therapiebeginn<br>• Therapieabbruch bei Nachweis einer Schwangerschaft<br><br>**Verschreibungsverfahren für ANA:**<br>Die empfohlene Dosis von Kineret beträgt 100 mg und wird als subkutane Injektion einmal täglich angewendet. Die Dosis sollte jeden Tag ungefähr zur gleichen Zeit gegeben werden. Kineret ist gebrauchsfertig in einer Fertigspritze erhältlich. Um Beschwerden an der Einstichstelle zu vermeiden, wird ein Wechsel der Einstichstelle empfohlen. Eine Dosisanpassung ist bei Patienten mit einem Alter über 60 Jahren nicht erforderlich. Die Dosierung, Art und Dauer der Anwendung entsprechen den Angaben für Erwachsene im Alter von 18 bis 64 Jahren. In klinischen Studien wurden insgesamt keine Unterschiede hinsichtlich Unbedenklichkeit oder Wirksamkeit zwischen Patienten im Alter von ≥ 65 Jahren und jüngeren Patienten beobachtet

	Bei Patienten mit leicht eingeschränkter Nierenfunktion (Kreatinin-Clearance (CLCr) 50 bis 80 ml/Minute) ist keine Dosisanpassung erforderlich. Patienten mit schwerer Nierenfunktionsstörung (CLCr < 30 ml/Minute) werden von der Studie ausgeschlossen.<br><br>Selten wurden im Zusammenhang mit ANA über Fälle mit allergischen Reaktionen berichtet. In der Mehrzahl handelte es sich bei diesen Reaktionen um makulopapulöse oder urtikarielle Hautausschläge. Weiterhin enthält die Nadelschutzkappe der Fertigspritze getrockneten Naturkautschuk (ein Latexderivat), der Allergien verursachen kann. Wenn ein Patient Symptome entwickelt, die auf eine allergische Reaktion hindeuten oder unerklärtes hohes Fieber vorliegt, wird ANA abgesetzt.<br><br>ANA wurde mit einer erhöhten Inzidenz für schwerwiegende Infektionen (1,8%) versus Placebo (0,7%) in Verbindung gebracht. Bei einer kleinen Zahl von Patienten mit Asthma war die Inzidenz einer schwerwiegenden Infektion bei Behandlung mit ANA höher (4,5%), als bei Behandlung mit Placebo (0%). Bei Vorliegen einer schwerwiegenden Infektion wird der Patient aus der Studie ausgeschlossen.<br><br>Die Unbedenklichkeit und Wirksamkeit von ANA bei Patienten mit chronischen Infektionen wurden nicht untersucht. Patienten die eine Vorgeschichte von rezidivierenden Infektionen haben oder die Grunderkrankungen haben, die sie für Infektionen prädisponieren können, werden von der Studie ausgeschlossen.<br><br>Die Anwendung von ANA war bei 2,4% der Patienten mit einer Neutropenie (absolute Neutrophilenzahl (ANZ) < 1,5 x 10^{9}/l) assoziiert, verglichen mit 0,4% bei Placebopatienten. Bei keinem dieser Patienten war das Auftreten der Neutropenie mit schwerwiegenden Infektionen verbunden. Eine Neutropenie (ANZ < 1,5 x 10^{9}/l) ist als Ausschlusskriterium des Prüfprotokolls definiert. Die Anzahl der Neutrophilen wird vor Behandlung mit ANA sowie während der Studie monatlich in den ersten 6 Behandlungsmonaten und danach vierteljährlich bestimmt. Bei Patienten, die neutropenisch werden (ANZ < 1,5 x 10^{9}/l), wird die Anzahl der Neutrophilen sorgfältig überwacht und sie werden aus der Studie ausgeschlossen.<br><br>Bei gleichzeitiger Anwendung von ANA und einem Tetanus-/Diphterie-Toxin-Impfstoff wurde in einer Placebo-kontrollierten klinischen Studie (n = 126) kein Unterschied in der anti-Tetanus Antikörperantwort zwischen der mit ANA oder der mit Placebo behandelten Gruppe beobachtet. Es liegen keine Daten über die Wirkungen von Impfungen mit anderen inaktivierten Antigenen bei mit ANA behandelten Patienten vor. Über die Wirkung von Lebendimpfstoffen oder über die sekundäre Übertragung einer Infektion durch

	Lebendimpfstoffe bei Patienten, die ANA erhalten, sind keine Daten verfügbar. Während des Studienverlaufs ist die Verabreichung von Lebendimpfstoffen zu vermeiden.<br><br>Die Auswirkungen einer Behandlung mit ANA auf vorbestehende maligne Erkrankungen wurden nicht untersucht. Daher ist eine maligne Tumorerkrankung als Ausschlusskriterium des Prüfprotokolls definiert<br><br>ANA ist temperaturempfindlich und muss daher im Kühlschrank gelagert (2°C - 8°C) werden. Es darf dabei nicht eingefroren werden. Zum Zwecke der ambulanten Anwendung kann ANA einmalig über einen Zeitraum von 12 Stunden, bei Temperaturen nicht über 25°C, außerhalb des Kühlschrankes gelagert werden. Nach diesem Zeitraum darf ANA nicht wieder zurück in den Kühlschrank gelegt werden, sondern muss verworfen werden.<br><br>Dokumentation unerwünschter Ereignisse an:<br>Präsenzvisite V1 bis V8<br>Telefonvisite TV1 bis TV7<br>Dokumentation der Verträglichkeit an:<br>Präsenzvisite V2 bis V8<br>Internetvisite WebV1 bis WebV51
Abbruchkriterien	Für jeden Patienten wird die Beendigung oder der vorzeitige Abbruch der klinischen Studie dokumentiert. Für jeden Patienten mit einem vorzeitigen Abbruch der klinischen Studie wird die Ursache des Studienabbruchs ermittelt und dokumentiert. Die folgenden Kriterien können für den Abbruch der klinischen Studie hinreichend sein:<br><br>• Laborparameter außerhalb des Normbereiches (z. B.):<br>o Neutropenie (ANZ < 1,5 x 10^9/l)<br>o Schwere Nierenfunktionsstörung (CLCr < 30 ml/Minute)<br>o Erhöhung um das 5-fache des oberen Normwertes der ALT/AST-Werte<br>• Eintritt einer Schwangerschaft<br>• Signifikante Prüfplanverletzung<br>• Fehlende Compliance<br>• Schwerwiegende Infektion<br>• Klinische Hinweise auf eine allergische Reaktion einschließlich makulopapulöser oder urtikarieller Hautausschläge und einer unklaren Erhöhung der Körpertemperatur<br>• Schwerwiegende Hautreaktionen, insbesondere bullöse Hautreaktionen einschließlich des Stevens-Johnson-Syndroms und der toxisch-epidermalen Nekrolyse<br>• Pathologische Untersuchungsbefunde einschließlich der Elektrokardiographie<br>• Persönlicher Wunsch des Patienten<br>• Widerruf der Patienteneinwilligung<br>• Kontaktverlust<br>• Ortswechsel, der eine Einhaltung des Protokolls nicht

	möglich macht<br>• Tod des Patienten<br><br><u>Abbruch der gesamten Studie:</u><br>Ein vorzeitiges Studienende und Abbruch der gesamten Studie ist unter folgenden Bedingungen möglich, wenn:<br><br>• Ein Patient eine schwere Infektion erleidet mit möglicher, wahrscheinlicher oder sicherer Kausalität zur Studienmedikation und die nicht im Kontext der Grunderkrankung zu erklären ist.<br>• Neue und replizierte wissenschaftliche Erkenntnisse über ANA während der Laufzeit der Studie entstanden sind, die eine Unterlegenheit der ANA-Behandlung zeigen.<br>• Hinweise auf eine Häufung von schwerwiegenden unerwünschten Ereignissen vorliegen.<br><br>Die Entscheidung über den Abbruch der Studie wird durch die Studienleitung getroffen.<br>Nach Studienabbruch werden folgende Ereignisse dokumentiert und an den Sponsor der Studie weitergeleitet:<br>• Alle schwerwiegenden unerwünschten Ereignisse, die innerhalb von 30 Tagen nach der letzten Behandlung mit ANA aufgetreten sind.<br>• Jede Schwangerschaft, die innerhalb von 84 Tagen (12 Wochen) nach der letzten Dosis von ANA nachgewiesen wurde.
Statistische Auswertung	Hauptziel dieser konzeptionellen Pilotstudie, die gleichzeitig die Basis für eine statistisch begründbare Planung einer Phase II-Wirksamkeitsstudie darstellt, ist der Nachweis einer Verträglichkeit und verschiedener sekundärer Endpunkte. Der primäre Endpunkt und mehrere sekundäre Endpunkte werden zunächst explorativ untersucht und deskriptiv ausgewertet. Die konzeptionelle Studie wird mit n=20 Patienten durchgeführt. Ähnliche Fragestellungen werden in der Literatur mit einer vergleichbaren Patientenzahl beschrieben. Sekundäre Endpunkte werden je nach Skalierung und Verteilungstyp der erfassten Daten mit parametrischen Tests oder verteilungsunabhängigen Verfahren geprüft. Die biometrische Auswertung erfolgt in SAS®.
Pharmakologisch-toxikologische Prüfung	**Pharmakologische Eigenschaften:**<br>***Pharmakodynamische Eigenschaften:***<br>ANA gehört zur pharmakotherapeutische Gruppe der Immunsuppressiva (ATC-Code: L04AC03). ANA ist eine auf die Aminosäuren 26–177 verkürzte und endständig L–methionylierte Isoform des humanen Interleukin–1–Rezeptorantagonisten und besitzt eine Sequenzlänge von 153 Aminosäuren. Die Herstellung erfolgt durch *Escherichia coli-Stämme* mit rekombinanten Methoden. ANA neutralisiert die biologische Aktivität von Interleukin-1α (IL-1α) und Interleukin-1β (IL-1β) indem es kompetitiv deren Bindung an

den Interleukin-1 Typ I Rezeptor (IL-1RI) hemmt. Interleukin-1 (IL-1) ist ein zentrales proinflammatorisches Zytokin, das als Mediator vieler zellulärer Antworten dient. ANA hemmt *in vitro* die von IL-1 hervorgerufenen Reaktionen, einschließlich der Induktion von Stickstoffmonoxid und der Produktion von Prostaglandin E2.

Pharmakokinetische Eigenschaften

Die absolute Bioverfügbarkeit von ANA nach einer subkutanen Bolusinjektion von 70 mg liegt bei gesunden Probanden (n = 11) bei 95%. Der Absorptionsprozess ist der limitierende Faktor für die Geschwindigkeit der Elimination von ANA aus dem Plasma nach subkutaner Injektion. Die maximalen Plasmakonzentrationen von ANA wird 3 bis 7 Stunden nach subkutaner Gabe von ANA in klinisch relevanten Dosen (1 bis 2 mg/kg; n = 18) erreicht; die terminale Halbwertszeit lag zwischen 4 bis 6 Stunden. Nach täglicher subkutaner Gabe für bis zu 24 Wochen wurde keine unerwartete Kumulation von ANA beobachtet. Der Einfluss demographischer Kovariablen auf die Pharmakokinetik von ANA wurde mittels einer pharmakokinetischen Populationsanalyse untersucht, die 341 Patienten umfasste, welche bis zu 24 Wochen lang täglich eine subkutane Injektion von Anakinra in Dosen von 30, 75 und 150 mg erhielten. Die geschätzte Clearance von ANA nahm mit steigender Kreatinin-Clearance und zunehmendem Körpergewicht zu. Die pharmakokinetische Populationsanalyse zeigte, dass der mittlere Plasma-Clearancewert nach subkutaner Bolusgabe bei Männern ca. 14% höher lag als bei Frauen und bei Probanden < 65 Jahren um ca. 10% höher lag als bei Probanden ≥ 65 Jahren. Geschlecht und Alter waren jedoch nach Einstellung bzgl. Kreatinin-Clearance und Körpergewicht keine signifikanten Faktoren für die mittlere Plasma-Clearance.

Pharmokologisch-toxikologische Prüfung:

ANA zeigte bei der Ratte in Dosierungen, die bis zu hundertmal höher lagen als die beim Menschen, keine Auswirkungen auf Fruchtbarkeit, Frühentwicklung, Entwicklung des Embryos/Fetus oder peri- und postnatale Entwicklung. Beim Kaninchen wurden keine Auswirkungen auf die Entwicklung des Embryos/Fetus bei Dosierungen beobachtet, welche die Dosierung beim Menschen um das Hundertfache überstiegen. In Standardtestreihen zur Ermittlung des DNA-Gefährdungspotentials, induzierte ANA in Bakterien- oder Säugetierzellen keine Genmutationen. Darüber hinaus war durch ANA die Inzidenz von Chromosomenanomalien oder Mikronuklei in Knochenmarkzellen bei Mäusen nicht erhöht. Es wurden keine Langzeitstudien durchgeführt, um das kanzerogene Potential von ANA zu untersuchen. Daten von Mäusen mit IL-1ra Überexpression und von IL-1ra Mutant Knock-out Mäusen ergaben keine Hinweise auf ein erhöhtes Risiko für eine Tumorentwicklung. Eine konventionell durchgeführte toxikologische und toxikokinetische Interaktionsstudie an

	Ratten erbrachte keinen Hinweis darauf, dass ANA das toxikologische oder pharmakokinetische Profil von Methotrexat verändert.<br><br>**Pharmakokinetische Eigenschaften:**<br>*Absorption*:<br>Die absolute Bioverfügbarkeit von ANA nach einer subkutanen Bolusinjektion von 70 mg liegt bei gesunden Probanden (n = 11) bei 95%.<br><br>*Metabolismus*:<br>Zum gegenwärtigen Zeitpunkt ist genaue Verlauf und Charakter des ANA-Metabolismus beim Menschen nicht bekannt. Das Arzneimittel wird durch Hydrolyse mit anschließender Elimination der Abbauprodukte im Urin ausgeschieden.<br><br>*Elimination*:<br>Bei Patienten mit rheumatoider Arthritis wurden die maximalen Plasmakonzentrationen von ANA 3 bis 7 Stunden nach subkutaner Gabe von Anakinra in klinisch relevanten Dosen (1 bis 2 mg/kg; n = 18) erreicht; die terminale Halbwertszeit lag zwischen 4 bis 6 Stunden. Die geschätzte Clearance von Anakinra nahm mit steigender Kreatinin-Clearance und zunehmendem Körpergewicht zu. Die pharmakokinetische Populationsanalyse zeigte, dass der mittlere Plasma-Clearancewert nach subkutaner Bolusgabe bei Männern ca. 14% höher lag als bei Frauen und bei Probanden < 65 Jahren um ca. 10% höher lag als bei Probanden ≥ 65 Jahren. Geschlecht und Alter waren jedoch nach Einstellung bzgl. Kreatinin-Clearance und Körpergewicht keine signifikanten Faktoren für die mittlere Plasma-Clearance<br><br>*Wechselwirkungen mit anderen Arzneimitteln*:<br>Wechselwirkungen von ANA mit anderen Arzneimitteln sind nicht durch gezielte klinische Studien untersucht worden. In klinischen Studien wurden keine Interaktionen zwischen ANA und anderen Arzneimitteln (einschließlich nichtsteroidaler Antiphlogistika, Kortikosteroiden und Basistherapeutika (DMARDs)) beobachtet.<br><br>In einer klinischen Studie mit Patienten, die als Basistherapie Methotrexat erhielten, zeigten Patienten, die ANA und Etanercept erhielten, eine höhere Inzidenz schwerwiegender Infektionen (7%) und Neutropenien als Patienten, die nur mit Etanercept behandelt wurden. Die Inzidenz war ebenfalls höher als in vorangegangenen Studien, in denen ANA allein angewendet wurde. Die gleichzeitige Behandlung mit ANA und Etanercept zeigte keinen verbesserten klinischen Nutzen. Diese Ergebnisse haben keine klinische Bedeutung bei der ALS, da keine Komedikation mit Methotrexat oder Etanercept erfolgt. Aufgrund der fehlenden Komedikation mit Methotrexat oder Etanercept ist eine geringere Inzidenz schwerer Infektionen bei ALS-Patienten mit ANA-Behandlung zu erwarten. Durch das Ausschlusskriterium eines klinisch

schwerwiegenden Hypoventilationssyndrom (Vitalkapazität < 50 %) besteht ein geringeres Risko einer Pneumonie bei ALS-Patienten mit ANA-Behandlung.

Orale Kontrazeptiva:
Zur Anwendung von ANA bei Frauen mit oraler Kontrazeption liegen keine ausreichenden Daten vor.

Kanzerogenität/Mutagenität:
Das Risiko, Lymphome zu entwickeln, kann für Patienten mit rheumatoider Arthritis (RA) höher (im Durchschnitt 2-3-fach) sein. In klinischen Studien zeigten Patienten mit RA, welche ANA erhielten, eine höhere Inzidenz für Lymphome als die für die Normalbevölkerung zu erwartende Rate. Die Rate unter ANA-Behandlung stimmt jedoch mit derjenigen überein, die im Allgemeinen für Patienten mit rheumatoider Arthritis berichtet wurde.Die Inzidenzrate für maligne Erkrankungen war in klinischen Studien bei mit ANA behandelten Patienten und mit Placebo behandelten Patienten vergleichbar und unterschied sich nicht von der in der Normalbevölkerung. Ausserdem war die Gesamtinzidenz maligner Erkrankungen bei Patienten nach 3 Jahren ANA-Exposition nicht erhöht.

Schwangerschaft und Stillzeit:
Zur Anwendung von ANA bei schwangeren Frauen liegen keine ausreichenden Daten vor. Tierexperimentelle Studien gaben keine Hinweise auf direkte oder indirekte schädigende Wirkungen in Bezug auf Schwangerschaft, Entwicklung des Embryos/Fetus, Geburt oder postnatale Entwicklung. Frauen im gebärfähigen Alter müssen während der ANA-Behandlung eine zuverlässige Verhütungsmethode anwenden. Wenn bei einer Patientin oder der Partnerin eines Patienten die Menstruation ausbleibt oder vermutlich ausgeblieben ist oder es zu Abnormalitäten bei der Monatsblutung kommt oder der Verdacht auf eine Schwangerschaft besteht, sind ein Schwangerschaftstest und eine fachärztlich-gynäkologische Beratung erforderlich. Wenn während der ANA-Behandlung eine Schwangerschaft bei einer Patientin auftritt, sollte die Behandlung unverzüglich abgebrochen werden. Die Patientin oder die schwangere Partnerin wird zur weiteren Untersuchung und Beratung an ein in der Teratologie erfahrenen Gynäkologen und Geburtshelfer verwiesen.

Es ist nicht bekannt, ob ANA in die Muttermilch abgegeben wird. Frauen, die ANA einnehmen, sollten nicht stillen.

Nebenwirkungen:
In allen placebokontrollierten Studien war die am häufigsten berichtete Nebenwirkung mit ANA eine Reaktion an der Einstichstelle, welche bei der Mehrzahl der Patienten leicht bis mäßig war. Eine Reaktion an der Einstichstelle war der häufigste Grund für einen Studienabbruch bei mit ANA behandelten Patienten. Die Inzidenz von schwerwiegenden Nebenwirkungen bei der empfohlenen Dosis von ANA (100 mg/Tag) ist vergleichbar mit der unter Placebo (7,1%

	verglichen mit 6,5% in der Placebogruppe). Die Inzidenz einer schwerwiegenden Infektion lag bei den mit ANA behandelten Patienten höher als bei den Patienten, die Placebo erhielten (1,8% versus 0,7%). Ein Abfall der Neutrophilen trat bei Patienten, die ANA erhielten häufiger auf als unter Placebo.<br><br>*Reaktionen an der Einstichstelle*<br>Die häufigste und durchgehend berichtete Nebenwirkung, die der Behandlung mit ANA zugeschrieben wurde, waren Reaktionen an der Einstichstelle. Die Mehrheit (95%) der Reaktionen an der Einstichstelle wurde als leicht bis mäßig berichtet. Diese wurden in der Regel durch eines oder mehrere der folgenden Symptome charakterisiert: Erythem, Ekchymose, Entzündung und Schmerzen. Bei einer Dosis von 100 mg/Tag entwickelten 71% der Patienten eine Reaktion an der Einstichstelle, verglichen mit 28% der mit Placebo behandelten Patienten. Diese Reaktion wurde normalerweise innerhalb der ersten 4 Wochen der Therapie berichtet. Die mediane Dauer der oben erwähnten typischen Symptome betrug 14 bis 28 Tage. Nach dem ersten Behandlungsmonat war die Entwicklung von Reaktionen an der Einstichstelle bei Patienten, die zuvor keine Reaktionen an der Einstichstelle gezeigt hatten, selten.<br><br>*Schwerwiegende Infektionen*<br>Die Häufigkeit schwerwiegender Infektionen in Studien, die mit der empfohlenen Dosis (100 mg/Tag) durchgeführt wurden, war 1,8% bei mit ANA behandelten Patienten und 0,7% bei mit Placebo behandelten Patienten. Die Rate schwerwiegender Infektionen blieb über einen Beobachtungszeitraum von bis zu 3 Jahren stabil. Die beobachteten Infektionen waren vorwiegend bakteriellen Ursprungs wie zum Beispiel Entzündungen des Unterhautgewebes, Pneumonie sowie Knochen- und Gelenkinfektionen. Die meisten Patienten setzten die Studienmedikation fort, nachdem die Infektion vollständig zurückgegangen war. Es gab im Studienverlauf keine Todesfälle aufgrund schwerwiegender Infektionsepisoden. Seltene Fälle von opportunistischen Infektionen, einschließlich solcher, welche auf Pilz-, Mycobakterien-, Bakterien- und Viruspathogene zurückzuführen sind, wurden in klinischen Studien und bei der Anwendung nach der Zulassung beobachtet. Infektionen wurden in allen Organsystemen beobachtet. Über Infektionen wurde bei Patienten, welche ANA alleine, oder in Kombination mit immunosupressiven Substanzen erhielten, berichtet.<br><br>*Neutropenie*<br>In placebokontrollierten Studien mit ANA war die Behandlung mit geringen Abnahmen der mittleren Gesamtleukozyten werte und der absoluten Neutrophilenzahl (ANZ) assoziiert. Eine Neutropenie (ANZ < 1,5 x 10^9/l) wurde bei 2,4% der mit ANA behandelten Patienten verglichen mit 0,4% der Placebopatienten berichtet

	Immunogenität<br>In klinischen Studien wurden 3% der erwachsenen Patienten mindestens einmal während der Studie seropositiv auf Antikörper, die die biologische Wirkung von ANA neutralisieren können, getestet. Das Auftreten dieser Antikörper war üblicherweise vorübergehend und nicht mit klinisch unerwünschten Reaktionen oder verminderter Wirksamkeit assoziiert. Zusätzlich wurden in einer klinischen Studie 6% der pädiatrischen Patienten mindestens einmal während der Studie seropositiv auf Antikörper, die die biologische Wirkung von ANA neutralisieren können, getestet.<br><br>*Teratogenität:*<br>Tierexperimentelle Studien gaben keine Hinweise auf direkte oder indirekte schädigende Wirkungen in Bezug auf Schwangerschaft, Entwicklung des Embryos/Fetus, Geburt oder postnatale Entwicklung.<br><br>**Anwendungsgebiete:**<br>ANA ist zur Behandlung der Symptome der rheumatoiden Arthritis in Kombination mit Methotrexat bei Patienten indiziert, die nur unzureichend auf Methotrexat allein ansprechen. Die empfohlene Dosis von ANA beträgt 100 mg und wird als subkutane Injektion einmal täglich angewendet. Die Dosis sollte jeden Tag ungefähr zur gleichen Zeit gegeben werden. ANA ist gebrauchsfertig in einer Fertigspritze erhältlich. Um Beschwerden an der Einstichstelle zu vermeiden, wird ein Wechsel der Einstichstelle empfohlen.<br><br>**Riluzol**<br>Riluzol ist als Standardmedikation zugelassen und wird unabhängig vom Prüfplan vom Studienzentrum oder einem sonstigen behandelnden Arzt verordnet. Riluzol 50 mg steht als Filmtablette zur Verfügung. Pharmazeutischer Hersteller ist Sanofi Synthelabo-Aventis und wird mit dem Handelsnamen Rilutek-Filmtabletten® vertrieben. Eine Filmtablette Rilutek enthält 50 mg Riluzol. Die Dosierung beträgt 2 Filmtabletten/Tag. Die Anwendung erfolgt zur Verlängerung der Lebenserwartung oder zur Hinauszögerung der Zeit bis zum Einsatz der mechanischen Beatmung bei Patienten mit ALS. Als Gegenanzeigen von Riluzol sind Lebererkrankungen oder initiale Transaminasenerhöhungen über den 3-fachen Normbereich definiert. Anwendungsbeschränkungen ergeben sich bei vorbestehenden Leber- und Nierenfunktionsstörungen. Als Nebenwirkungen sind die folgenden Symptome bekannt: Asthenie, Nausea, Kopfschmerzen, abdominelle Schmerzen, Erbrechen, Benommenheit, Tachykardie, Schläfrigkeit, periorale Parästhesien und sehr selten: anaphylaktischen Reaktionen, Angioödeme und Pankreatitis. In Einzelfällen ist eine Neutropenie beschrieben worden. Die folgenden Wechselwirkungen wurden bisher dokumentiert: Verringerung der Elimination durch Hemmstoffe des Cyto-

	chrom P450-Isoenzyms CYP1A2 (z. B. Koffein, Diclofenac, Diazepam, Clomipramin, Imipramin, Fluvoxamin, Theophyllin, Amitriptylin und Chinolon); Beschleunigung der Elimination durch Induktoren von CYP1A2 (z.B. Zigarettenrauch, auf Holzkohle gegrillte Nahrung, Rifampizin und Omeprazol).
Mögliche Risiken, Kontraindikationen und Maßnahmen bei evtl. Zwischenfällen	*Schwerwiegende Infektionen*<br>ANA wurde mit einer erhöhten Inzidenz für schwerwiegende Infektionen (1,8%) versus Placebo (0,7%) in Verbindung gebracht. Bei Vorliegen einer schwerwiegenden Infektion wird die Behandlung mit ANA nicht weitergeführt.<br><br>*Neutropenie:*<br>Die Anwendung von ANA war bei 2,4% der Patienten mit einer Neutropenie assoziiert, verglichen mit 0,4% bei Placebopatienten. Eine Behandlung mit ANA darf nur begonnen werden, wenn die absolute Anzahl der Neutrophilen 1,5 x 10^9/l überschreitet. Die Leukozytenzahl und das Differentialblutbild werden im Studienverlauf nach Protokoll überwacht. Bei einer Verminderung der Neutrophilenanzahl während der ANA-Behandlung < 1,5 x 10^9/l ist eine Beendigung der Behandlung angezeigt.<br><br>*Allergische Reaktionen:*<br>Während klinischer Studien waren allergische Reaktionen im Zusammenhang mit der Anwendung von ANA selten. In der Mehrzahl handelte es sich bei diesen Reaktionen um makulopapulöse oder urtikarielle Hautausschläge. Mit Auftreten eines Hautausschlages wird die Behandlung mit ANA unmittelbar unterbrochen. Bei Vorliegen eines schweren Hautausschlags wird die Behandlung mit ANA nicht weitergeführt.<br><br>*Impfungen*<br>Bei gleichzeitiger Anwendung von ANA und einem Tetanus-/Diphterie-Toxin-Impfstoff wurde in einer Placebo-kontrollierten klinischen Studie (n = 126) kein Unterschied in der anti-Tetanus Antikörperantwort zwischen der mit ANA oder der mit Placebo behandelten Gruppe beobachtet. Es liegen keine Daten über die Wirkungen von Impfungen mit anderen inaktivierten Antigenen bei mit ANA behandelten Patienten vor. Über die Wirkung von Lebendimpfstoffen oder über die sekundäre Übertragung einer Infektion durch Lebendimpfstoffe bei Patienten, die ANA erhalten, sind keine Daten verfügbar. Lebendimpfstoffe werden daher nicht gleichzeitig mit ANA verabreicht.<br><br>*Gleichzeitige Behandlung mit ANA und TNF-Antagonisten*<br>Die gleichzeitige Anwendung von ANA und Etanercept wurde mit einem erhöhten Risiko schwerwiegender Infektionen und Neutropenien in Verbindung gebracht, verglichen mit der Anwendung von Etanercept allein. Eine gleichzeitige Anwendung von ANA und Etanercept oder anderen TNF-Antagonisten führt zur Beendigung der Behandlung mit ANA.

Risiko-Nutzen-Abwägung	Die ALS ist eine der schwerwiegendsten neurologischen Erkrankungen, die durch progrediente Extremitätenparesen, eine Immobilisierung und den Verlust der Sprech- und Schluckfunktionen bei Erhalt der kognitiven und emotionalen Funktionen gekennzeichnet ist. Durch fortschreitende Paresen der Atemmuskulatur kommt es zum Tod durch respiratorische Insuffizienz nach einem mittleren Krankheitsverlauf von 3-5 Jahren. Eine effektive Therapie der ALS steht nicht zur Verfügung. Das Medikament Riluzol (Rilutek®, Aventis-Sanofi-Synthelabo) führt zu einer geringen Lebenszeitverlängerung von 2-3 Monaten. Vor diesem Hintergrund ist die Identifizierung und Evaluierung weiterer neuroprotektiver Substanzen dringend erforderlich. ANA erscheint aufgrund seiner antiinflammatorischen und Interleukin-1-Rezeptor-antagonistischen Eigenschaften als interessante Prüfmedikation, die das Potential einer Wirksamkeit bei der ALS aufweist. Diese Annahme beruht auf den morphologischen, neurochemischen und molekularbiologischen Kenntnisstand zur Pathogenese der ALS sowie auf den Ergebnissen einer tierexperimentellen Studie mit ANA im transgenen Mausmodell der ALS.<br><br>Die hauptsächlichen Nebenwirkungen von ANA bestehen aus allergischen Reaktionen und schwerwiegenden Infektionen. Patienten mit häufigen Infektionen oder die Grunderkrankungen haben, die sie für Infektionen prädisponieren können, werden nicht in die Studie eingeschlossen. Bei Patienten, die im Studienverlauf einen schweren Hautausschlag und eine schwerwiegende Infektion erleiden, wird die Behandlung mit ANA nicht weitergeführt. Aufgrund des Schweregrades und der Geringgradigkeit der Therapieoptionen bei der ALS erscheint die ANA-Behandlung bei der ALS mit dem Ziel einer Verträglichkeits- und Sicherheitsstudie gerechtfertigt.<br><br>Studienspezifische invasive Untersuchungsmethoden sind innerhalb der Verträglichkeitsstudie weder vorgesehen noch erforderlich. Im Zusammenhang mit der Ermittlung der Arzneimittelsicherheit ist eine Blutabnahme zu definierten Visiten vorgesehen, in der paraklinische Parameter der Inflammation, der Leber- und Nierenfunktion sowie der hämatologischen Befunde erhoben werden. Die Risiken entsprechen denen einer venösen Blutabnahme und sind als äußerst gering einzuschätzen.

4. Ablaufschema

Visitenplan

Präsenzvisiten

Visitennummer	V1	V2	V3	V4	V5	V6	V7	V8
Woche	W0<br>+/- 2 Tage	W0<br>+/- 2 Tage	W4<br>+/- 2 Tage	W8<br>+/- 2 Tage	W12<br>+/- 2 Tage	W24<br>+/- 2 Tage	W36<br>+/- 2 Tage	W52<br>+/- 2 Tage
Einwilligungserklärung	+	-	-	-	-	-	-	-
Anamnese	+	-	-	-	-	-	-	-
Zwischenanamnese	-	+	+	+	+	+	+	+
Abfrage der Verträglichkeit	-	-	+	+	+	+	+	+
Körperliche Untersuchung	+	+	+	+	+	+	+	+
Manuelle Muskeltestung (MMT)	+	-	-	-	+	+	+	+
Vitalkapazität	+	-	+	+	+	+	+	+
Blutdruck und Puls	+	+	+	+	+	+	+	+
EKG	+	+	+	+	+	+	+	+
Messung der Körperlänge	+	-	-	-	-	-	-	-
Gewicht	+	-	-	+	+	+	+	+
Labordiagnostik	+	-	+	+	+	+	+	+
Schwangerschaftstest*	+	-	-	-	-	-	-	-
Abfrage unerwünschter Ereignisse	+	+	+	+	+	+	+	+
Selbstbewertung mit ALS-FRS-R	+	+	+	+	+	+	+	+
Medikamentenausgabe	-	+	+	+	+	+	+	-
Abschlussuntersuchung**								+

**bei gebärfähigen Frauen; gebärfähig definiert als: bis 2 Jahre nach Menopause*
*** Im Falle eines vorzeitigen Studienabbruches ist die Abschlussuntersuchung zum Zeitpunkt des Studienabbruches durchzuführen*

Visitenplan

Telefonvisiten

Visitennummer	TV1	TV2	TV3	TV4	TV5	TV6	TV7
Woche	W16	W20	W28	W32	W40	W44	W48
Abfrage unerwünschter Ereignisse	+	+	+	+	+	+	+
Selbstbewertung mit ALS-FRS-R	+	+	+	+	+	+	+

Visitenplan
Internetvisiten

Visitennummer	Web V1	Web V2	Web V3	Web V4	Web V5	Web V6	Web V7	Web V8	Web V9	Web V10	Web V11	Web V12	Web V13	Web V14
Woche	W2	W3	W4	W5	W6	W7	W8	W9	W10	W11	W12	W13	W14	W15
Selbstbewertung mit ALS-FRS-R	+	+	+	+	+	+	+	+	+	+	+	+	+	+
Selbstbewertung der Reaktionen an der Einstichstelle	+	+	+	+	+	+	+	+	+	+	+	+	+	+
Selbstbewertung von Kopfschmerzen (VRS)	+	+	+	+	+	+	+	+	+	+	+	+	+	+

Visitennummer	Web V15	Web V16	Web V17	Web V18	Web V19	Web V20	Web V21	Web V22	Web V23	Web V24	Web V25	Web V26	Web V27	Web V28
Woche	W16	W17	W18	W19	W20	W21	W22	W23	W24	W25	W26	W27	W28	W29
Selbstbewertung mit ALS-FRS-R	+	+	+	+	+	+	+	+	+	+	+	+	+	+
Selbstbewertung der Reaktionen an der Einstichstelle	+	+	+	+	+	+	+	+	+	+	+	+	+	+
Selbstbewertung von Kopfschmerzen (VRS)	+	+	+	+	+	+	+	+	+	+	+	+	+	+

Visitennummer	Web V29	Web 30	Web V31	Web V32	Web V33	Web V34	Web V35	Web V36	Web V37	Web V38	Web V39	Web V40	Web V41	Web V42
Woche	W30	W31	W32	W33	W34	W35	W36	W37	W38	W39	W40	W41	W42	W43
Selbstbewertung mit ALS-FRS-R	+	+	+	+	+	+	+	+	+	+	+	+	+	+
Selbstbewertung der Reaktionen an der Einstichstelle	+	+	+	+	+	+	+	+	+	+	+	+	+	+
Selbstbewertung von Kopfschmerzen (VRS)	+	+	+	+	+	+	+	+	+	+	+	+	+	+

Visitennummer	Web V43	Web V44	Web V45	Web V46	Web V47	Web V48	Web V49	Web V50	Web V51
Woche	W44	W45	W46	W47	W48	W49	W50	W51	W52
Selbstbewertung mit ALS-FRS-R	+	+	+	+	+	+	+	+	+
Selbstbewertung der Reaktionen an der Einstichstelle	+	+	+	+	+	+	+	+	+
Selbstbewertung von Kopfschmerzen (VRS)	+	+	+	+	+	+	+	+	+

Visitenplan
Übersicht aller Visiten

Woche	Präsenzvisite	Telefonvisite	Internetvisite
0	+ (Screening)		
1	+ (Einschluss)		
2			+
3			+
4	+		+
5			+
6			+
7			+
8	+		+
9			+
10			+
11			+
12	+		+
13			+
14			+
15			+
16		+	+
17			+
18			+
19			+
20		+	+
21			+
22			+
23			+
24	+		+
25			+
26			+
27			+
28		+	+
29			+
30			+
31			+
32		+	+
33			+
34			+
35			+
36	+		+
37			+
38			+
39			+
40		+	+
41			+
42			+
43			+
44		+	+
45			+
46			+
47			+
48		+	+
49			+
50			+
51			+
52	+ (Abschluss)		+

5.1 Einleitung:

Ausgangssituation:

Die Amyotrophe Lateralsklerose (ALS) ist eine fortschreitende und einheitlich tödliche neurodegenerative Erkrankung, für die derzeit keine Heilung besteht und nur ineffektive pharmakologische Therapieoptionen zur Verfügung stehen [1]. Es handelt sich um eine Erkrankung, die durch den selektiven und kombinierten Untergang von 1. und 2. Motoneuron charakterisiert ist. Dabei entstehen typische Symptomenkomplexe, die isoliert oder überlagert auftreten. Symptome des geschädigten 1. motorischen Neurons sind zentrale Paresen mit gesteigertem Muskeltonus und Eigenreflexen sowie pathologischen Fremdreflexen. Das Syndrom der Progressiven Muskelatrophie (PMA) läßt sich auf eine Schädigung des 2. motorischen Neurons zurückführen. Außer Myatrophien bestehen schlaffe Paresen, erloschene oder verminderte Eigenreflexe, Muskelatrophien und Faszikulationen. Die ALS führt durch eine chronische Hypoventilation und resultierende respiratorische Insuffizienz in einem mittleren Krankheitsverlauf von 3-5 Jahren zum Tod [1]. Die Betroffenen erfahren schwerwiegende Einschränkungen der manuellen Funktionen und ihrer Mobilität. Im Krankheitsverlauf der ALS kommt es bei der überwiegenden Zahl der Patienten zu einer Sprechstörung, während das Sprachverständnis und andere kognitive und intellektuelle Funktionen erhalten bleiben. Durch den Verlust der Sprechfunktionen, der natürlichen Nahrungsaufnahme und aller motorischer Leistungen mit Aussparung der Augenmotorik ist die ALS eine der schwerwiegendsten Erkrankungen der neurologischen Medizin. Die Patienten erkranken zwischen dem 20. und 70. Lebensjahr mit einer Häufung in der 5. Lebensdekade. 6000 bis 8000 Patienten sind in Deutschland betroffen, etwa 1500 Personen erfahren jährlich die Diagnose einer ALS.

Die bisherigen Erkenntnisse zur Ursache der ALS gehen im Wesentlichen auf eine seltene familiäre Form der ALS zurück [2]. Im Fall der erblichen ALS führen Mutationen im SOD1-Gen zur Umwandlung natürlich vorkommender körpereigener Eiweiße in ein toxisches Produkt. Das mutierte Eiweiß führt durch einen bisher unverstandenen Prozess zu einem selektiven Untergang der motorischen Nervenzellen. Ebenfalls unvollständig verstanden ist die Ursache der nichterblichen ALS, die bei etwa 95 – 98 % aller ALS-Patienten vorliegt. Zu vermuten ist, dass zahlreiche Gene mit unterschiedlichen Lokalisationen im menschlichen Genom zum Risiko einer ALS beitragen können. Es handelt sich um eine multifaktorielle Erkrankung, bei der verschiedene, akkumulierende Risikofaktoren verantwortlich sind. Dabei müssen mehrere genetische, exogene und stochastische Faktoren zu einem spezifischen Muster zusammentreffen.

Bei der ALS steht derzeit keine pharmakologische Therapie zur Verfügung, die den pathogenetischen Prozess signifikant beeinflussen und die Progression der Krankheitssymptome verhindern kann. Seit 1996 steht das Medikament Riluzol als zugelassene Therapie gegen die ALS zur Verfügung. Die Zulassung erfolgte als Ergebnis von 2 placebokontrollierten, multizentrischen Wirksamkeitsstudien, die eine geringgradige Lebenszeitexpansion von 85 Tagen im Vergleich zu Placebo in einem Studienzeitraum von 18 Monaten zeigen konnten [3,4]. Der Mechanismus der klinischen Wirksamkeit ist derzeit noch unbekannt. Als möglicher Mechanismus der geringgradigen Krankheitsverlangsamung wird eine Modulation der glutamatergen Neurotransmission, die Hemmung der synaptischen Glutamatfreisetzung und Verminderung exzitotoxischer Effekte diskutiert. Die Behandlung mit Riluzol in einer Dosis von 100 mg/Tag ist als Standardbehandlung bei der ALS etabliert.

Seit der Zulassung von Riluzol wurden mehrere Medikamentenstudien der Phasen II und III in Kombinationsbehandlung mit Riluzol durchgeführt. Kontrollierte Studien mit Gabapentin und Lamotrigin zielten auf eine Hemmung der glutamatvermittelten Neuronenschädigung. Dieser Ansatz erfolgte komplementär zu dem hypothetischen antiglutamatergen Wirkmechanismus von Riluzol. Die Studien mit Gabapentin und Lamotrigin sind akademische Untersuchungen, die als multizentrische, placebokontrollierte und randomisierte Studien einen Evidenzgrad Klasse Ib aufwiesen. Beide Studien zeigten keinen

Vorteil der Prüfsubstanz. Eine weitere Studie mit dem antiapoptotischen Medikament TCH346 wurde ebenfalls placebokontrolliert, multizentrisch und randomisiert durchgeführt und verlief ohne positive Beeinflussung des primären Studienendpunktes. Die Ergebnisse sind bisher unpubliziert. Die unveröffentlichten Ergebnisse zeigen keinen Vorteil der jeweiligen Prüfsubstanz. Eine weitere Therapiestrategie umfasste den Einsatz von neuronalen Wachstumsfaktoren. Die kontrollierten Studien mit CNTF, IGF1, BDNF und GDNF wurden ohne signifikanten therapeutischen Effekt beendet. Der Einsatz von trophischen Faktoren erfolgte unter der Vorstellung, dass durch neuronale Wachstumsfaktoren der Prozess der neuronalen Sprossung unterstützt und eine Lebenszeitexpansion erreicht werden kann. Aktuell wird eine multizentrische Studie mit Glatirameracetat in Kombination mit Riluzol im Vergleich zur Riluzol-Behandlung in Kombination mit Placebo durchgeführt. Die Studie wird vom pharmazeutischen Hersteller TEVA unterstützt. Glatirameracetat ist ein zugelassenes Medikament zur Behandlung der Multiplen Sklerose. Die Rationale der Anbindung bei der ALS geht auf experimentelle und klinische Daten zurück, die bei der ALS eine inflammatorische Komponente im pathogenetischen Mechanismus der Motoneuronendegeneration zeigen konnten. Die bisherigen negativen Ergebnisse kontrollierter Studien und die geringen Therapieeffekte von Riluzol machen die Entwicklung neuartiger und symptomatischer Therapiestrategien erforderlich. Aufgrund der begrenzten pathogenetischen Information ist im Zeitraum der geplanten klinischen Studie die Entwicklung ätiologisch-definierter Therapiestrategien nicht wahrscheinlich.

Riluzol (Rilutek®, Aventis-Sanofi-Synthelabo) stellt die einzige bisher zugelassene pharmakologische Therapieoption mit der Indikation der ALS dar [3,4]. Es handelt sich um einen Inhibitor der präsynaptischen Glutamatfreisetzung, der eine neuroprotektive Zielstellung verfolgt. Riluzol führt zu einer geringgradigen Lebenszeitverlängerung von 1,5 – 2, 5 Monaten. Eine effektive Therapie der ALS steht damit nicht zur Verfügung. Trotz der geringen klinischen Wirksamkeit ist die Substanz als Standardmedikation eingeführt und wird mit einer nahezu vollständigen Marktdurchdringung in Deutschland eingesetzt. Vor dem Hintergrund der geringen Effektivität der bisherigen Standardbehandlung ist die Identifizierung weiterer neuroprotektiver und symptomatischer Therapieoptionen dringend erforderlich.

Der Einsatz von Anakinra (ANA) bei der ALS verfolgt ein bisher wenig untersuchtes Konzept, das eine Verminderung der ALS-assoziierten Neuroinflammation beabsichtigt. Die morphologischen, neurochemischen, molekulargenetischen und tierexperimentellen Untersuchungsergebnisse zeigen siginifikante inflammatorische Prozesse im ZNS von ALS-Patienten bzw. von ALS-Modellsystemen, so dass eine Immunmodulation und antinflammatorische Therapiestrategie bei der ALS als plausibel erscheint [5-8]. Die Neuroinflammation ist ein wesentlicher pathogenetischer Faktor der Motoneuronendegeneration. Inflammatorische Veränderungen sind bei der sporadischen ALS, der autosomal-dominanten Form der ALS sowie im transgenen SOD1-Mausmodell der ALS nachweisbar. Morphologisch ist eine Akkumulation von aktivierter Mikroglia, Astrozyten und T-Zellen festzustellen [9,10]. Begleitende biochemische Veränderungen umfassen die Aktivierung der pro-inflammatorischen Cystein-Protease Caspase-1. Als Folge wird eine Entzündungsreaktion ausgelöst, bei der das Zytokin Interleukin 1β (IL-1ß) in seine biologisch aktive Form überführt und freigesetzt wird. Der Entzündungsmediator IL-1 vermittelt die Rekrutierung und Aktivierung von phagozytischen Zellen und induziert eine Immunantwort [11-13]. Diese durch IL-1 vermittelte Entzündung wirkt sich schädigend auf Zellen des zentralen Nervensystems aus und beschleunigt somit die Neurodegeneration im Verlauf der Erkrankung. IL-1 ist als Schlüsselmolekül der Neuroinflammation bei der ALS anzusehen Für ANA konnte in Tierexperimenten eine positive Wirkung auf ein transgenes ALS-Mausmodell gezeigt werden [14]. Die tierexperimentellen Daten und pathogenetischen Erkenntnisse zur Rolle der Neuroinflammation bei der ALS unterstützen die Hypothese, dass ANA über das Potential verfügt, die ALS-assoziierte Neurodegeneration zu verzögern. Untersuchungen im

transgenen ALS-Mausmodell (SOD1-G93A) zeigten bei einer Behandlung mit ANA eine signifikante Lebenszeitverlängerung [14].

ANA ist durch seine molekulare Größe nur in einem sehr geringe Maße in der Lage, in das zentrale Nervensystem (ZNS) vorzudringen. Ursache für die geringe Durchlässigkeit in das ZNS ist die Blut-Hirn-Schranke. Auf Grund dieser pharmakologischen Eigenschaften zielt ANA auf den Krankheitsprozess am dem 2. motorischen Neuron, das ebenfalls bei der ALS betroffen ist. Die ANA-Behandlungsstrategie ist vor allem bei ALS-Patienten geeignet, die eine überwiegende Betroffenheit des 2. Motoneurons aufweisen. Im Vordergrund steht die Behandlung von Patienten mit einer Progressiven Muskelatrophie (PMA). Dabei handelt es sich um eine klinische Variante der ALS, die eine schwere und ausschließliche Schädigung des 2. Motoneurons aufweisen. Klinisch stellt sich die Schädigung des 2. Motoneurons durch ausgeprägte Myatrophien, schlaffe Lähmungen und erloschene oder verminderte Eigenreflexe dar. Eine Behandlung von ALS-Patienten mit einer ausgeprägten Spastik scheint nach dem heutigen Kenntnisstand nicht geeignet, da die Ursache der Spastik in einer ALS-bedingten Schädigung von ZNS-Strukturen liegt, die von ANA nicht erreicht werden können. Daher sind Patienten mit einer überwiegenden Spastik von der Studie ausgeschlossen.

Auf der Grundlage neuroinflammatorischer Veränderungen bei der ALS besteht eine wissenschaftliche Rationale einer klinischen Studie mit ANA, das über antiinflammatorische pharmakologische Eigenschaften verfügt und selektive Hemmung des inflammatorischen Zytokins IL-1 aufweist. Die primäre Zielstellung der klinischen Studie beinhaltet die Sicherheit und Verträglichkeit von ANA in Kombinationsbehandlung mit Riluzol bei Patienten mit ALS. In einer offenen Verträglichkeitsstudie soll überprüft werden, ob eine subkutane Injektion von 100 mg ANA toleriert wird. Die Verträglichkeitsstudie ist als Grundlage für eine Phase IIb/III-Wirksamkeitsstudie anzusehen.

Das Ziel der Verträglichkeitsstudie mit ANA besteht darin, die Sicherheit einer Kombinationsbehandlung von Riluzol mit ANA bei der ALS zu untersuchen und eine Wirksamkeitsstudie von ANA in Kombination mit Riluzol vorzubereiten. Klinische Erfahrungen mit ANA bei der ALS oder anderen neurologischen Erkrankungen liegen bisher nicht vor. Bei der systematischen Erfassung von unerwünschten Arzneimittelwirkungen werden die allergische Reaktion, schwerwiegende Infektionen und eine Neutropenie als wesentliche Risiken beschrieben. Die geplante Dosierung der ANA-Verträglichkeitsstudie bei der ALS gehen auf die Dosierungsempfehlungen bei der rheumatoiden Arthritis zurück.

Stand des Wissens über die Prüfmedikation:

ANA-Amgen 100 mg Injektionslösung in einer Fertigspritze in Kombination mit Methotrexat ist indiziert zur Behandlung der rheumatoiden Arthritis, die nur unzureichend auf Methotrexat allein anspricht. Die klinische Wirksamkeit von ANA wurde in zwei randomisierten placebokontrollierten Doppelblind-Studien an 920 Probanden überprüft [15,16]. In einer Dosisfindungsstudie erhielten 419 Patienten mit aktivierter rheumatoider Arthritis entweder täglich 0,04 bis 2,0 mg ANA pro kg Körpergewicht oder Placebo. Zusätzlich behandelte man die Studienteilnehmer mit Methotrexat. Die Ansprechrate wurde nach 12 beziehungsweise 24 Wochen anhand der American College of Rheumatology (ACR)-Kriterien bestimmt. Die Anteile der Patienten, deren ACR-Kriterien sich um mindestens 20 Prozent verbesserten, waren nach 12 Wochen in allen Verumgruppen signifikant höher als unter Methotrexat plus Placebo. In einer zweiten Untersuchung, bei der die Patienten 100 mg ANA oder Placebo pro Tag plus Methotrexat erhielten, bestätigten sich die positiven Ansprechraten sowohl nach 12 als auch nach 24 Wochen. Nach 24 Wochen besserten sich die nach dem ACR-Schema definierten Symptome unter ANA bei 38 Prozent um mindestens ein Fünftel, bei 17 Prozent um die Hälfte und bei 6 Prozent sogar um knapp zwei Drittel. Unter Placebo waren es 22, 8 beziehungsweise 2 Prozent. In beiden Studien senkte der IL-1-Antagonist zudem typische Entzündungsparameter. Dieser Effekt hielt während des gesamten Studienzeitraums an.

Zudem bewerteten sowohl die Ärzte als auch die Patienten den Therapieerfolg von ANA anhand eines Fragebogens signifikant besser als den des Placebos.

Pharmakologische Eigenschaften von ANA

ANA ist ein humaner Interleukin-1 Rezeptorantagonist (r-metHuIL-1ra), der in *Escherichia coli*-Zellen durch rekombinante DNA-Technologie hergestellt wird. ANA neutralisiert die biologische Aktivität von Interleukin-1α (IL-1α) und Interleukin-1β (IL-1β) indem es kompetitiv deren Bindung an den Interleukin-1 Typ I Rezeptor (IL-1RI) hemmt. Interleukin-1 (IL-1) ist ein zentrales proinflammatorisches Zytokin, das als Mediator vieler zellulärer Antworten dient. ANA hemmt *in vitro* die von IL-1 hervorgerufenen Reaktionen, einschließlich der Induktion von Stickstoffmonoxid und der Produktion von Prostaglandin E2 und/oder von Kollagenase. Aufgrund der entzündungshemmenden Eigenschaften wird ANA bei Patienten mit rheumatoider Arthritis eingesetzt.

Absorption:

Die absolute Bioverfügbarkeit von ANA nach einer subkutanen Bolusinjektion von 70 mg liegt bei gesunden Probanden (n = 11) bei 95%. Der Absorptionsprozess ist der limitierende Faktor für die Geschwindigkeit der Elimination von ANA aus dem Plasma nach subkutaner Injektion. Bei Patienten mit rheumatoider Arthritis wurden die maximalen Plasmakonzentrationen von ANA 3 bis 7 Stunden nach subkutaner Gabe von ANA in klinisch relevanten Dosen (1 bis 2 mg/kg; n =18) erreicht; die terminale Halbwertszeit lag zwischen 4 bis 6 Stunden.

Verteilung:

Das genaue Verteilungsprofil von ANA wurde beim Menschen noch nicht beschrieben. Das exakte Verteilungsvolumen ist nicht bekannt. ANA zeigt keine signifikante Penetration der Blut-Hirn-Schranke.

Metabolismus:

Zum gegenwärtigen Zeitpunkt ist der genaue Verlauf und Charakter des ANA-Metabolismus beim Menschen nicht bekannt. Das Arzneimittel wird durch spontane Hydrolyse mit anschließender Elimination der Abbauprodukte im Urin ausgeschieden. Der hepatische Metabolismus und die renale Ausscheidung von ANA sind minimal.

Elimination:

Bei Patienten mit rheumatoider Arthritis wurde nach täglicher subkutaner Gabe für bis zu 24 Wochen keine unerwartete Kumulation von ANA beobachtet. Der Einfluss demographischer Kovariablen auf die Pharmakokinetik von ANA wurde mittels einer pharmakokinetischen Populationsanalyse untersucht, die 341 Patienten umfasste, welche bis zu 24 Wochen lang täglich eine subkutane Injektion von ANA in Dosen von 30, 75 und 150 mg erhielten. Die geschätzte Clearance von ANA nahm mit steigender Kreatinin-Clearance und zunehmendem Körpergewicht zu. Die pharmakokinetische Populationsanalyse zeigte, dass der mittlere Plasma-Clearancewert nach subkutaner Bolusgabe bei Männern ca. 14% höher lag als bei Frauen und bei Probanden < 65 Jahren um ca. 10% höher lag als bei Probanden ≥ 65 Jahren. Geschlecht und Alter waren jedoch nach Einstellung bzgl. Kreatinin-Clearance und Körpergewicht keine signifikanten Faktoren für die mittlere Plasma-Clearance.

Wechselwirkungen mit anderen Arzneimitteln:

Wechselwirkungen von ANA mit anderen Arzneimitteln sind nicht durch gezielte klinische Studien untersucht worden. In klinischen Studien wurden keine Interaktionen zwischen ANA und anderen Arzneimitteln (einschließlich nichtsteroidaler Antiphlogistika, Kortikosteroiden und Basistherapeutika) beobachtet.

Gleichzeitige Behandlung mit Kineret und TNF-Antagonisten
In einer klinischen Studie mit Patienten, die als Basistherapie Methotrexat erhielten, zeigten Patienten, die ANA und Etanercept erhielten, eine höhere Inzidenz schwerwiegender Infektionen (7%) und Neutropenien als Patienten, die nur mit Etanercept behandelt wurden. Die Inzidenz war ebenfalls höher als in vorangegangenen Studien, in denen ANA allein angewendet wurde. Die gleichzeitige Behandlung mit ANA und Etanercept zeigte keinen verbesserten klinischen Nutzen. Die gleichzeitige Anwendung von ANA und Etanercept oder einem anderen TNF-Antagonisten wird nicht empfohlen.

Orale Kontrazeptiva:
Die Wechselwirkungen von ANA mit oralen Kontrazeptiva sind nicht durch gezielte klinische Studien untersucht worden.

Kanzerogenität/Mutagenität:
In Standardtestreihen zur Ermittlung des DNA-Gefährdungspotentials, induzierte ANA in Bakterien- oder Säugetierzellen keine Genmutationen. Darüber hinaus war durch ANA die Inzidenz von Chromosomenanomalien oder Mikronuklei in Knochenmarkzellen bei Mäusen nicht erhöht. Es wurden keine Langzeitstudien durchgeführt, um das kanzerogene Potential von ANA zu untersuchen. Daten von Mäusen mit IL-1ra Überexpression und von IL-1ra Mutant Knock-out Mäusen ergaben keine Hinweise auf ein erhöhtes Risiko für eine Tumorentwicklung. Eine konventionell durchgeführte toxikologische und toxikokinetische Interaktionsstudie an Ratten erbrachte keinen Hinweis darauf, dass ANA das toxikologische oder pharmakokinetische Profil von Methotrexat verändert

Schwangerschaft und Stillzeit:
Zur Anwendung von ANA bei schwangeren Frauen liegen keine ausreichenden Daten vor. Tierexperimentelle Studien gaben keine Hinweise auf direkte oder indirekte schädigende Wirkungen in Bezug auf Schwangerschaft, Entwicklung des Embryos/Fetus, Geburt oder postnatale Entwicklung. ANA zeigte bei der Ratte in Dosierungen, die bis zu hundertmal höher lagen als die beim Menschen, keine Auswirkungen auf Fruchtbarkeit, Frühentwicklung, Entwicklung des Embryos/Fetus oder peri- und postnatale Entwicklung. Beim Kaninchen wurden keine Auswirkungen auf die Entwicklung des Embryos/Fetus bei Dosierungen beobachtet, welche die Dosierung beim Menschen um das Hundertfache überstiegen. Es ist nicht bekannt, ob ANA in die Muttermilch abgegeben wird. Frauen, die ANA einnehmen, sollten nicht stillen.

Nebenwirkungen:
Die häufigsten Nebenwirkungen in Verbindung mit der Anwendung von ANA sind Reaktionen an der Einstichstelle (≥1/10) und Kopfschmerzen (≥1/10). Reaktionen an der Einstichstelle waren bei der Mehrzahl der Patienten leicht bis mäßig. Eine Reaktion an der Einstichstelle war der häufigste Grund für einen Studienabbruch bei mit ANA behandelten Patienten. Weitere häufige unerwünschte Arzneimittelwirkungen sind schwerwiegende Infektionen (≥1/100, <1/10), allergische Reaktionen und eine Neutropenie (≥1/100, <1/10). Seltene unerwünschte Arzneimittelwirkungen (>0,01 %) sind opportunistische Infektionen.

Mögliche Risiken, Kontraindikation und Maßnahmen bei Zwischenfällen mit ANA:
Die Anwendung von ANA kann unerwünschte Wirkungen verursachen.

Reaktionen an der Einstichstelle
Die häufigste und durchgehend berichtete Nebenwirkung, die der Behandlung mit ANA zugeschrieben wurde, waren Reaktionen an der Einstichstelle. Die Mehrheit (95%) der Reaktionen an der Einstichstelle wurde als leicht bis mäßig berichtet. Diese wurden in der Regel durch eines oder mehrere der folgenden Symptome charakterisiert: Erythem, Ekchymose, Entzündung und Schmerzen. Bei einer Dosis von 100 mg/Tag entwickelten 71% der Patienten eine Reaktion an der Einstichstelle, verglichen mit 28% der mit Placebo

behandelten Patienten. Diese Reaktion wurde normalerweise innerhalb der ersten 4 Wochen der Therapie berichtet. Die mediane Dauer der oben erwähnten 6 typischen Symptome betrug 14 bis 28 Tage. Nach dem ersten Behandlungsmonat war die Entwicklung von Reaktionen an der Einstichstelle bei Patienten, die zuvor keine Reaktionen an der Einstichstelle gezeigt hatten, selten. Patienten die an der Studie teilnehmen werden in der Anwendung der Fertigspritze geschult.

Kopfschmerzen
Eine häufige Nebenwirkungen in Verbindung mit der Anwendung von ANA sind Kopfschmerzen. Bei Vorliegen eines Kopfschmerzes kann die Behandlung mit ANA weitergeführt werden.

Schwerwiegende Infektionen
Die Häufigkeit schwerwiegender Infektionen in Studien, die mit der empfohlenen Dosis (100 mg/Tag) durchgeführt wurden, war 1,8% bei mit ANA behandelten Patienten und 0,7% bei mit Placebo behandelten Patienten. Die Rate schwerwiegender Infektionen blieb über einen Beobachtungszeitraum von bis zu 3 Jahren stabil. Die beobachteten Infektionen waren vorwiegend bakteriellen Ursprungs wie zum Beispiel Entzündungen des Unterhautgewebes, Pneumonie sowie Knochen- und Gelenkinfektionen. Die meisten Patienten setzten die Studienmedikation fort, nachdem die Infektion vollständig zurückgegangen war. Es gab im Studienverlauf keine Todesfälle aufgrund schwerwiegender Infektionsepisoden. Mit Auftreten einer ANA-assoziierten schwerwiegenden Infektion wird die Behandlung abgebrochen.

Opportunistische Infektionen
Seltene Fälle von opportunistischen Infektionen, einschließlich solcher, welche auf Pilz-, Mycobakterien-, Bakterien- und Viruspathogene zurückzuführen sind, wurden in klinischen Studien und bei der Anwendung nach der Zulassung beobachtet. Infektionen wurden in allen Organsystemen beobachtet. Über Infektionen wurde bei Patienten, welche ANA alleine, oder in Kombination mit immunosupressiven Substanzen erhielten, berichtet. Mit Auftreten einer ANA-assoziierten opportunistischen Infektion ist es erforderlich, die Behandlung abzubrechen.

Neutropenie:
In Verbindung mit der klinischen Anwendung von ANA wurden eine Leuko- und Neutropenie berichtet. Die Leukozytenzahl und das Differentialblutbild werden im Studienverlauf nach Protokoll überwacht. Bei einer Verminderung der Neutrophilenanzahl während der ANA-Behandlung $< 1{,}5 \times 10^9/l$ ist eine Beendigung der Behandlung angezeigt.

Allergische Reaktionen:
Während klinischer Studien waren allergische Reaktionen im Zusammenhang mit der Anwendung von ANA selten. In der Mehrzahl handelte es sich bei diesen Reaktionen um makulopapulöse oder urtikarielle Hautausschläge. Falls es zu einer schweren allergischen Reaktion kommt, muss die Gabe von ANA abgebrochen und eine geeignete Behandlung eingeleitet werden. Die Nadelschutzkappe der Fertigspritze enthält getrockneten Naturkautschuk (ein Latexderivat), der Allergien verursachen kann. Mit Auftreten eines Hautausschlages wird die Behandlung mit ANA unmittelbar unterbrochen. Bei Vorliegen eines schweren Hautausschlags wird die Behandlung mit ANA nicht weitergeführt.

Maligne Erkrankungen
Das Risiko, Lymphome zu entwickeln, kann für Patienten mit rheumatoider Arthritis (RA) höher (im Durchschnitt 2-3-fach) sein. In klinischen Studien zeigten Patienten, welche ANA erhielten, eine höhere Inzidenz für Lymphome als die für die Normalbevölkerung zu erwartende Rate. Die Rate unter ANA-Behandlung stimmt jedoch mit derjenigen überein, die im Allgemeinen für Patienten mit rheumatoider Arthritis berichtet wurde. Die Inzidenzrate für maligne Erkrankungen war in klinischen Studien bei mit ANA behandelten Patienten und mit

Placebo behandelten Patienten vergleichbar und unterschied sich nicht von der in der Normalbevölkerung. Ausserdem war die Gesamtinzidenz maligner Erkrankungen bei Patienten nach 3 Jahren Kineret-Exposition nicht erhöht.

Immunogenität

In klinischen Studien wurden 3% der erwachsenen Patienten mindestens einmal während der Studie seropositiv auf Antikörper, die die biologische Wirkung von Anakinra neutralisieren können, getestet. Das Auftreten dieser Antikörper war üblicherweise vorübergehend und nicht mit klinisch unerwünschten Reaktionen oder verminderter Wirksamkeit assoziiert. Zusätzlich wurden in einer klinischen Studie 6% der pädiatrischen Patienten mindestens einmal während der Studie seropositiv auf Antikörper, die die biologische Wirkung von ANA neutralisieren können, getestet

Detaillierte Informationen zu den chemischen und physikalischen Eigenschaften, die Ergebnisse der toxikologischen Prüfung und pharmakodynamischen Untersuchungen, die Daten zur Pharmakokinetik und Ergebnisse bisher durchgeführter klinischer Studien einschließlich der bisher bekannten Arzneimittelwirkungen und deren Häufigkeit, die Erkenntnisse über Nebenwirkungen und Ergebnisse der klinischen Prüfung sind der Prüferinformation des Herstellers (Investigator's Brochure) zu entnehmen.

Riluzol

Riluzol ist als Standardmedikation zugelassen und wird unabhängig vom Prüfplan vom Studienzentrum oder einem sonstigen behandelnden Arzt verordnet. Riluzol 50 mg steht als Filmtablette zur Verfügung. Pharmazeutischer Hersteller ist Sanofi Synthelabo-Aventis und wird mit dem Handelsnamen Rilutek-Filmtabletten® vertrieben. Eine Filmtablette Rilutek enthält 50 mg Riluzol. Die Dosierung beträgt 2 Filmtabletten/Tag. Die Anwendung erfolgt zur Verlängerung der Lebenserwartung oder zur Hinauszögerung der Zeit bis zum Einsatz der mechanischen Beatmung bei Patienten mit ALS. Als Gegenanzeigen von Riluzol sind Lebererkrankungen oder initiale Transaminasenerhöhungen über den 3-fachen Normbereich definiert. Anwendungsbeschränkungen ergeben sich bei vorbestehenden Leber- und Nierenfunktionsstörungen. Als Nebenwirkungen sind die folgenden Symptome bekannt: Asthenie, Nausea, Kopfschmerzen, abdominelle Schmerzen, Erbrechen, Benommenheit, Tachykardie, Schläfrigkeit, periorale Parästhesien und sehr selten: anaphylaktischen Reaktionen, Angioödeme und Pankreatitis. In Einzelfällen ist eine Neutropenie beschrieben worden. Die folgenden Wechselwirkungen wurden bisher dokumentiert: Verringerung der Elimination durch Hemmstoffe des Cytochrom P450-Isoenzyms CYP1A2 (z. B. Koffein, Diclofenac, Diazepam, Clomipramin, Imipramin, Fluvoxamin, Theophyllin, Amitriptylin und Chinolon); Beschleunigung der Elimination durch Induktoren von CYP1A2 (z.B. Zigarettenrauch, auf Holzkohle gegrillte Nahrung, Rifampizin und Omeprazol).

5.2. Fragestellung und Begründung

Auf der Grundlage neuroinflammatorischer Veränderungen bei der ALS besteht eine wissenschaftliche Rationale einer klinischen Studie mit ANA. Das Medikament verfügt über antiinflammatorische Eigenschaften und weist eine hochselektive Modulation des proinflammatorischen Zytokins IL-1 auf. Die primäre Zielstellung der klinischen Studie beinhaltet die Sicherheit und Verträglichkeit von ANA in Kombinationsbehandlung mit Riluzol bei Patienten mit ALS. In einer offenen Verträglichkeitsstudie soll überprüft werden, ob eine subkutane Injektion von 100 mg ANA toleriert wird. Die Verträglichkeitsstudie ist als Grundlage für eine Phase IIb/III-Wirksamkeitsstudie anzusehen. Die Hypothese der Verträglichkeitsstudie beinhaltet, dass die Anzahl der unerwünschten Arzneimittelwirkungen, der unerwünschten Ereignisse und der schwerwiegenden unerwünschten Ereignisse bei Patienten in einer Behandlung mit 100 mg ANA in Kombination mit Riluzol 100 mg keine signifikante Zunahme aufweist. Dabei soll überprüft werden, ob ANA in Kombinationsbehandlung mit Riluzol in einer chronischen Anwendung über 12 Monate eine hinreichende Verträglichkeit aufweist. Mit diesen Vorhaben wird eine Wirksamkeitsstudie

vorbereitet, sofern die Ergebnisse eine ausreichende Verträglichkeit von ANA bei der ALS zeigen. Für die Etablierung einer neuartigen Therapiestrategie besteht eine medizinische Notwendigkeit, da die bisherige Standardtherapie mit Riluzol sehr geringgradige klinische Effekte aufweist und eine dringende Optimierung erfordert. Das Studienvorhaben ist ethisch vertretbar, da auf der Basis der vorliegenden Studienergebnisse der rheumatoiden Arthritis eine gute Verträglichkeit zu erwarten ist. Aus den genannten Studien werden Tagesdosierungen bis 2,0 mg ANA pro kg Körpergewicht berichtet. Die geplante offene Verträglichkeitsstudie zur Evaluierung der Sicherheit und Verträglichkeit von ANA bei der ALS ist mit einer Dosis von 100 mg geplant, die bei der rheumatoiden Arthritis ausreichend toleriert wurde.

Begründung des Behandlungsverfahrens:
Es handelt sich um eine offene Verträglichkeitsstudie zur Evaluierung der Sicherheit und Verträglichkeit von 100 mg ANA bei Patienten mit ALS. Die Behandlung mit ANA erfolgt in Kombination mit Riluzol. Die Riluzol-Behandlung ist als Standardtherapie der ALS anzusehen. Die Studie wird offen durchgeführt. Ein wesentliches Einschlusskriterium in die Studie ist die Patienteneinwilligung in die Behandlung mit ANA. Aufgrund der Gewährleistung einer Standardtherapie mit Riluzol sowie einer gleichwertigen symptomatischen Therapie erscheint das gewählte Behandlungsverfahren als ethisch vertretbar. ANA zeigt keine signifikante Penetration der Bluthirnschranke. Aus diesem Grund ist das primäre Zielsubstrat dieser Studie das 2. Motoneuron. Es werden daher nur ALS-Patienten (revidierte El Escorial-Kriterien) mit einer klinischen ALS-Variante der Progressiven Muskelatrophie (PMA) oder mit dominanter Affektion des 2. Motoneurons in die Studie eingeschlossen.

5.3. Risiko-Nutzen-Abwägung

Die ALS ist eine der schwerwiegendsten neurologischen Erkrankungen, die durch progrediente Extremitätenparesen, Immobilisierung sowie den Verlust der Sprech- und Schluckfunktionen bei Erhalt der kognitiven und emotionalen Funktionen gekennzeichnet ist. Durch fortschreitende Paresen der Atemmuskulatur kommt es zum Tod durch respiratorische Insuffizienz nach einem mittleren Krankheitsverlauf von 3-5 Jahren. Eine effektive Therapie der ALS steht nicht zur Verfügung. Das Medikament Riluzol (Rilutek®, Aventis Sanofi Synthelabo) führt zu einer geringen Lebenszeitverlängerung von 2-3 Monaten. Vor diesem Hintergrund ist die Identifizierung und Evaluierung weiterer neuroprotektiver Substanzen dringend erforderlich. ANA erscheint aufgrund seiner antiinflammatorischen Eigenschaften als interessante Prüfmedikation, die das Potential einer Wirksamkeit bei der ALS aufweist. Diese Annahme beruht auf dem morphologischen, neurochemischen und molekularbiologischen Kenntnisstand zur Pathogenese der ALS sowie auf den Ergebnissen einer tierexperimentellen Studie mit ANA im transgenen Mausmodell der ALS. Die hauptsächliche Nebenwirkung von ANA besteht in einer schwerwiegenden Infektion. ALS-Patienten mit einer Vorgeschichte von rezidivierenden Infektionen, einer Grunderkrankungen, die für Infektionen prädisponiert oder einem klinisch schwerwiegenden Hypoventilationssyndrom (Vitalkapazität < 50 %) werden nicht in die Studie aufgenommen. Bei Vorliegen einer schwerwiegenden Infektion wird die Behandlung mit ANA beendet. Aufgrund des Schweregrades und der Geringgradigkeit der Therapieoptionen bei der ALS sowie der Risikominimierung von ANA-bedingten schwerwiegenden Infektionen erscheint die ANA-Behandlung bei der ALS gerechtfertigt.

Studienspezifische invasive Untersuchungsmethoden sind weder vorgesehen noch erforderlich. Ebenfalls ist eine Entnahme von Gewebsproben nicht vorgesehen. Im Zusammenhang mit der Ermittlung der Arzneimittelsicherheit ist eine Blutabnahme zu definierten Visiten vorgesehen, in der paraklinische Parameter der Inflammation, der Leber- und Nierenfunktion sowie der hämatologischen Befunde erhoben werden. Die Risiken entsprechen denen einer venösen Blutabnahme und sich als äußerst gering einzuschätzen.

6. Ziele der klinischen Prüfung

Das primäre Ziel der Studie besteht in der Überprüfung der Sicherheit und Verträglichkeit einer subkutanen Injektion mit ANA in Verbindung mit Riluzol bei ALS-Patienten. Der gewählte Stichprobenumfang ist nicht geeignet, eine Wirksamkeit von ANA in Kombination mit Riluzol bei der ALS zu beweisen. Dennoch soll durch sekundäre Studienendpunkte die Beeinflussung von ANA auf die ALS-Progressionsrate untersucht werden.

6.1. Hauptzielkriterium

Primäres Studienziel:

Eine Untersuchung von Anzahl und Schweregrad unerwünschter Ereignisse (UE), schwerwiegender unerwünschter Ereignisse (SUE), unerwünschter Arzneimittelwirkungen (UAW), unerwarteter unerwünschter Arzneimittelwirkungen (uUAW), schwerwiegender unerwünschter Arzneimittelwirkungen (SUAW), Verdachtsfälle unerwarteter schwerwiegender Arzneimittelwirkungen (SUSAR), sowie pathologischer Laborparameter.

6.2. Nebenzielkriterien

Sekundäre Studienziele:

- Evaluierung einer Langzeitverträglichkeit und Arzneimittelsicherheit von ANA in Kombination mit Riluzol
- Anzahl der Patienten, die eine kontinuierliche nicht-invasive Ventilation oder Tracheostomagestützte invasive Ventilation benötigen
- Anzahl der Patienten, die eine perkutane endoskopische Gastrostomie (PEG) benötigen,
- Anzahl der Patienten, die eine Behandlung mit ANA in Kombination mit Riluzol abgeschlossen haben
- Die Evaluierung einer klinischen Wirksamkeit von ANA auf die Progressionsrate der Paresen und funktionellen Einschränkungen bei der ALS, durch die vergleichende Untersuchung der intraindividuellen Progressionsrate des revidierten *ALS Functional Rating Scale* (ALS-FRS-R) in einem Studienzeitraum von 52 Wochen im Vergleich zur Progressionsrate vor der Studienteilnahme (Δ ALS-FRS-R) [17-19]
- Untersuchung der Wirksamkeit von ANA auf die respiratorische Funktion bei der ALS, die durch eine forcierte Vitalkapazität im Verlauf von 52 Wochen bestimmt wird [20]
- Untersuchung der Wirksamkeit von ANA auf die respiratorische Funktion von ALS-Patienten, die eine nichtinvasive Maskenventilation erhalten (Bestimmung der Ventilationszeit pro Tag)
- Ermittlung der Überlebenszeit oder des Zeitpunktes einer Tracheotomie durch respiratorische Insuffizienz (ausgenommen elektive Tracheotomie oder Tracheotomie zur Sekretkontrolle [20]

6.3. Studiendesign

Das Studienvorhaben ist eine offene Verträglichkeitsstudie zur Evaluierung der Sicherheit und Verträglichkeit von 100 mg ANA für die Dauer von 52 Wochen in Kombination mit Riluzol.

Studiencharakteristik

Das Vorhaben ist eine Studie, die der Evaluierung von Verträglichkeit und Sicherheit dient. Mit der rheumatoiden Arthritis wurden verschiedene Pilotstudien, Dosisfindungsstudien und Phase II/III-Studien mit ANA durchgeführt. Daten zur Sicherheit und Verträglichkeit von ANA bei neurodegenerativen Erkrankungen liegen bisher nicht vor, so dass in dieser Studie erste Verträglichkeitsinformationen von ANA bei der ALS generiert werden.

Studienorganisation:

Die Studie wird an einem Studienzentrum durchgeführt. Die Patientenzahl beträgt 20 Personen. Die ALS-Patienten werden monozentrisch rekrutiert. Aus den Erfahrungswerten früherer ALS-Therapiestudien ist ein Screening von etwa 30 ALS-Patienten erforderlich, um die geplante Zahl von 20 Patienten zu rekrutieren. Ebenfalls im Erfahrungsbereich

zurückliegender und publizierter ALS-Studien ist eine Drop-Out-Rate von höchstens 10% der Patienten anzunehmen, die bereits bei der Berechnung der erforderlichen Patientenzahl berücksichtigt ist. Die vorgesehene Patientenzahl kann zu über 50 % aus den Patienten der ALS-Ambulanz des Sponsors rekrutiert werden. Weitere Patienten sind über eine Patienteninformation auf den Internetseiten der ALS-Ambulanz an der Charité zu erreichen (www.als-charite.de). Im Zeitraum 11/2002 – 02/2010 wurden sechs Phase II-Studien an diesem Studienzentrum mit 165 Studienteilnehmern abgeschlossen. Die Vorerfahrungen haben erbracht, dass die Rekrutierung von ALS-Patienten aufgrund der etablierten Informationswege (Internetseiten, Publikationen, Selbsthilfeorganisationen, Medien-informationen) realistisch ist. Dabei kann das Screening von 30 ALS-Patienten in einem Rekrutierungszeitraum von 6 Monaten erreicht werden. In Deutschland ist von 6000 ALS-Patienten auszugehen, während die Anzahl der ALS-Neuerkrankungen 1500 Personen pro Jahr umfasst. Die Patientenstruktur am Studienzentrum zeigt zu 50 % eine Versorgung von Patienten aus Berlin und dem Bundesland Brandenburg, während der verbleibende Teil der Ambulanz-Patienten sich aus überregionalen Bundesländern vorstellt. Vor diesem Hintergrund ist eine überregionale Patientenrekrutierung möglich.

6.4. Zeitplan

Geplanter Beginn der Studie ist der 01.11.2010. Die Rekrutierungsphase erstreckt sich über 6 Monate im Zeitraum 11/2010 bis 04/2011. Der voraussichtliche Zeitpunkt des Therapieendes liegt 04/2012. Die geschätzte Dauer der Auswertung beträgt 2 Monate. Die Vorlage des Berichtes ist für 06/2012 geplant. Für das Ende der Therapiephase ist 04/2012 vorgesehen. Das reguläre Ende der gesamten Studie einschließlich der Auswertung und der Vorlage des Abschlussberichts ist der 31.06.2012. Der Einschluss des letzten Patienten wird durch die geplante Anzahl eingebrachter Patienten definiert. Die Rekrutierung von Studienpatienten ist mit 20 ALS-Patienten abgeschlossen.

7. Patienten

7.1. Einschlusskriterien:

- Patienten im Alter ≥ 18 Jahre und ≤ 80 Jahre.
- Klinische Diagnose einer ALS (revidierte El Escorial-Kriterien) mit dominanter Affektion des 2. motorischen Neurons oder der klinischen ALS-Variante einer Progressiven Muskelatrophie (PMA)
- Klinische Zeichen für eine Störung des 2. motorischen Neurons in mindestens einer anatomischen Region außerhalb der Bulbärregion
- Sporadische und familiäre ALS
- Symptombeginn mit Paresen sechs Monate bis vier Jahre vor Studieneinschluss
- Behandlung mit Riluzol 100 mg/Tag mindestens 3 Monate vor Studieneinschluss
- Schriftliche Patienteneinwilligung (laut AMG §40 (1) 3b)
- Kommunikationsfähigkeit, persönlich oder mit Hilfe einer bevollmächtigten Person
- Kommunikationsfähigkeit, verbal, manuell oder mittels elektronischem Kommunikationssystem
- Internetzugang, persönlich oder von bevollmächtigter Person
- Kühlmöglichkeit für ANA-Fertigspritzen (Kühlschrank) am Wohnort
- Keine Teilnahme an einer anderen Studie nach dem AMG während der Teilnahme an diesem Behandlungsplan
- Negativer Schwangerschaftstest oder mindestens 24 Monate postmenopausal

7.2. Ausschlusskriterien:

- Diagnose einer ALS mit überwiegender Schädigung des 1. motorischen Neurons ohne klinische Merkmale einer begleitenden Schädigung des 2. motorischen Neurons in mindestens einer anatomischen Region außerhalb der Bulbärregion (klinische Variante einer spastischen ALS)
- Diagnose der klinischen ALS-Variante einer Primären Lateralsklerose (PLS)

- Patienten mit bekannter Überempfindlichkeit gegenüber ANA, Riluzol oder einem der Hilfsstoffe
- Klinisch schwerwiegendes Hypoventilationssyndrom mit einer Vitalkapazität < 50 %
- Schwangere oder stillende Frauen
- Frauen im gebärfähigen Alter, die keine hocheffektiven Maßnahmen zur Verhütung einer Schwangerschaft anwenden können oder wollen (definiert als Pearl-Index <1)
- Klinisch-signifikante EKG-Veränderungen einschließlich einer symptomatischen Bradykardie
- Kontinuierliche nicht-invasive Beatmung mit einer ventilationsfreien Zeit < 2 Stunden
- Tracheotomie und mechanische Ventilation
- Laborparameter außerhalb des Normbereiches, die einer klinisch-signifikanten kardiovaskulären, pulmonologischen, hämatologischen, hepatologischen, metabolischen und renalen Erkrankung entsprechen
- Maligne Tumorerkrankung
- Schwere Nierenfunktionsstörung (Creatinine-Clearance < 30 ml/Minute)
- Vorgeschichte von rezidivierenden Infektionen oder eine Grunderkrankung, die für Infektionen prädisponiert
- Schwere Neutropenie (absolute Neutrophilenzahl < 1,5 x $10^{9/}$l)
- Monoklonale Gammopathie unklarer Signifikanz
- Infektionserkrankungen einschließlich der Infektion mit Tuberkulose, HIV, Hepatitis B und C
- Patienten mit klinisch schwerwiegenden Begleiterkrankungen, einschließlich psychiatrischer Erkrankungen
- Demenz und fehlende Einwilligungsfähigkeit
- Anamnese einer Epilepsie oder eines Gelegenheitsanfalls
- Behandlung mit einer sonstigen Studienmedikation < 3 Monate vor Studieneinschluss
- Krankheiten oder Funktionsstörungen, die nach Beurteilung des Prüfarztes eine Teilnahme an dieser Behandlung ausschließen
- Mangelnde Kooperationsbereitschaft
- Alle Kontraindikationen gegen Prüfmedikation (auch Hilfsstoffe der Arzneiform)
- Fehlende Bereitschaft zur Speicherung und Weitergabe pseudonymisierter Krankheitsdaten im Rahmen der klinischen Prüfung
- Gleichzeitige Anwendung von ANA und Etanercept oder anderen TNF-Antagonisten
- Unterbringung in einer Anstalt auf gerichtliche oder behördliche Anordnung (laut AMG §40 (1) 4)

8. Prüfmaterialien
8.1. Studienmedikation:
8.1.1. Prüfmedikation:
8.1.1.1 Beschreibung der Prüfmedikation:

Anakinra
Die Prüfmedikation ist Kineret 100 mg Injektionslösung in einer Fertigspritze. ANA ist ein rekombinanter humaner Interleukin-1 Rezeptorantagonist (r-metHuIL-1ra). In der Bundesrepublik Deutschland ist das Handelspräparat unter dem Namen Kineret 100 mg Injektionslösung verfügbar. Das Medikament ist für die subkutane Injektion vorgesehen. Die Studienmedikation wird in Form von Kineret 100 mg Injektionslösung in einer Fertigspritze zur Verfügung gestellt. Die Prüfmedikation wird durch die Charité Apotheke ausgehändigt. Die Prüfmedikation wird über die Apotheke von dem Hersteller, die Swedish Orphan Biovitrum AB bezogen. Die Prüfmedikation wird von der Charité Apotheke gelagert und über eine Kühlkette an die Patienten für die Dauer von 28 Tagen ausgeliefert.

Darreichungsform:
Die Fertigspritze enthält 100 mg ANA in 0,67 ml (150 mg/ml) und ist mit der Aufschrift „Kineret 100 mg“ gekennzeichnet. Die folgenden Hilfsstoffe kommen zur Anwendung: Natriumcitrat, Natriumchlorid, Natriumedetat, Polysorbat 80, Natriumhydroxid und Wasser für Injektionszwecke
Die Fertigspritze besteht aus Glas (Gasart I) mit einem Gummistopfen aus Bromobutylkautschuk. Die Nadelschutzkappe der Fertigspritze enthält getrockneten Naturkautschuk

Behältnis, Verpackung und Einheiten pro Packung:
Die Fertigspritzen aus Glas befinden sich in Packungen mit 28 Fertigspritzen.

Lagerung:
Die Lagerung von Kineret 100 mg Fertigspritzen ist im Kühlschrank bei 2 bis 8 Grad Celsius erforderlich. Die Lagerung in der Originalverpackung erfolgt zum Schutz vor Licht. Zum Zwecke der ambulanten Anwendung kann Kineret einmalig, vor Ablauf des Verfalldatums, über einen Zeitraum von 12 Stunden, bei Temperaturen nicht über 25°C, außerhalb des Kühlschrankes gelagert werden. Nach diesem Zeitraum darf das Produkt nicht wieder zurück in den Kühlschrank gelegt werden sondern muss verworfen werden.

Riluzol
Die Basisbehandlung von ALS-Patienten ist eine Monotherapie mit Riluzol 100 mg/Tag. Riluzol verfügt über geringgradige krankheitsmodifizierende Eigenschaften und führt zu einer moderaten Lebenszeitverlängerung. Das Medikament ist seit 1996 in Deutschland als pharmakologische Behandlung der ALS zugelassen. Die Behandlung mit Riluzol ist damit ein Einschlusskriterium für die ANA-Studie. Bei Medikationsbeginn mit Riluzol können bei einem Teil der Patienten Nebenwirkungen oder Veränderungen von Laborparametern auftreten. Um eine ursächliche Abgrenzung zu Anzahl und Schwere unerwünschter Ereignisse, schwerwiegender unerwünschter Ereignisse und unerwünschter Arzneimittelwirkungen von ANA in Kombination mit Riluzol zu gewährleisten, ist eine Riluzol-Behandlung für mindestens 1 Monat vor Studienbeginn erforderlich. Die Vormedikation und kontinuierliche Anwendung von Riluzol ist ein Einschlusskriterium.

Riluzol ist als Standardmedikation zugelassen und wird unabhängig vom Prüfplan vom Studienzentrum oder einem sonstigen behandelnden Arzt verordnet. Riluzol 50 mg steht als Filmtablette zur Verfügung. Pharmazeutischer Hersteller ist Sanofi Synthelabo-Aventis und wird mit dem Handelsnamen Rilutek-Filmtabletten® vertrieben. Eine Filmtablette Rilutek enthält 50 mg Riluzol. Die Dosierung beträgt 2 Filmtabletten/Tag. Die Anwendung erfolgt zur Verlängerung der Lebenserwartung oder zur Hinauszögerung der Zeit bis zum Einsatz der mechanischen Beatmung bei Patienten mit ALS.

Rilutek-Filmtabletten werden in einer Verpackung mit 56 Filmtabletten vertrieben. Die Lagerung erfolgt auf Raumtemperatur. Hinsichtlich Haltbarkeit und Lagerung bestehen keine besonderen Erfordernisse. Neben dem Inhaltsstoff Riluzol 50 mg sind die folgenden weiteren Bestandteile bekannt: Kalziumhydrogenphosphat, Mikrokristalline, Zellulose, hochdisperses Siliciumdioxid, Magnesiumstearat, Croscarmellose-Natrium, Hypromellose, Macrogol 6000 und Titandioxid.

8.1.1.2. Verzeichnis der Nebenwirkungen und Wechselwirkungen
Die Anwendung von ANA kann unerwünschte Wirkungen verursachen, die im Abschnitt 5.1. beschrieben werden.

Als Gegenanzeigen von Riluzol sind Lebererkrankungen oder initiale Transaminasenerhöhungen über den 3-fachen Normbereich definiert. Anwendungsbeschränkungen ergeben sich bei vorbestehenden Leber- und Nierenfunktionsstörungen. Als Neben-

wirkungen sind die folgenden Symptome bekannt: Asthenie, Nausea, Kopfschmerzen, abdominelle Schmerzen, Erbrechen, Benommenheit, Tachykardie, Schläfrigkeit, periorale Parästhesien und sehr selten: anaphylaktische Reaktionen, Angioödeme und Pankreatitis. In Einzelfällen ist eine Neutropenie beschrieben worden. Die folgenden Wechselwirkungen wurden bisher dokumentiert: Verringerung der Elimination durch Hemmstoffe des Cytochrom P450-Isoenzyms CYP1A2 (z. B. Koffein, Diclofenac, Diazepam, Clomipramin, Imipramin, Fluvoxamin, Theophyllin, Amitriptylin und Chinolon); Beschleunigung der Elimination durch Induktoren von CYP1A2 (z.B. Zigarettenrauch, auf Holzkohle gegrillte Nahrung, Rifampizin und Omeprazol).

8.1.2. Placebo und Vergleichsmedikation:

Untersuchungsziel ist die Verträglichkeit und Sicherheit von ANA in Kombination mit Riluzol bei Patienten mit ALS. Eine Placebobehandlung ist nicht vorgesehen. Der Verzicht auf eine Placebobehandlungsgruppe geht auf die umfangreichen Informationen zu den unerwünschten Arzneimittelwirkungen der bisherigen Standardbehandlung mit Riluzol zurück. Ein Studiendesign mit einem Placebo ist bei der Zielstellung der Studie nicht erforderlich und unbegründet.

8.2. Behandlungsschema

8.2.1. Anweisung für die Applikation:

Dosierung, Zeitpunkt und Dauer der Verabreichung von ANA:

Die Prüfmedikation besteht aus kontinuierlicher Behandlung mit 100 mg Riluzol und einer Dosis von 100 mg ANA/Tag für die Dauer von 52 Wochen. Die Einnahme erfolgt im Tagesverlauf mit 1 Fertigspritze Kineret 100 mg. Die Patienten erhalten eine Kombinationsbehandlung von 100 mg Riluzol. Die Riluzol-Behandlung besteht als Basismedikation und Vorbehandlung in unveränderter Dosierung im gesamten Behandlungszeitraum.

ANA wird durch den Hersteller, Swedish Orphan Biovitrum AB zur Verfügung gestellt. Die Apotheke am Prüfzentrum liefert das Medikament unter Aufrecherhaltung einer erforderlichen Kühlkette an die Wohnadresse des Patienten. In der häuslichen Umgebung wird das Medikament ebenfalls in einem Kühlschrank (2°C - 8°C) gelagert. In dem Studienzentrum erfolgt zu Studienbeginn eine individuelle Injektionsschulung der Patienten.

Erforderliche Maßnahmen zur Empfängnisverhütung:

Bei Patientinnen, die sich nach eigenen Angaben einer Hysterektomie unterzogen haben oder deren Menopause mehr als 24 Monate zurückliegt, muss der Arzt das Risiko einer Schwangerschaft einschätzen und über empfängnisverhütende Maßnahmen beraten.

Patientinnen, die sich keiner Hysterektomie unterzogen haben und deren Menopause weniger als 24 Monate zurückliegt, müssen mindestens einen Monat vor Behandlungsbeginn von ANA, während des Behandlungszeitraumes und einen Monat nach Beendigung der Behandlung zuverlässige Methoden zur Kontrazeption anwenden. Als zuverlässige Verhütungsmethode ist der gleichzeitige Einsatz einer hochwirksamen Methode der Empfängnisverhütung (Intrauterinpessar, hormonelle Kontrazeptiva, Tubenligatur, Vasektomie beim Partner) und mindestens eine zusätzliche wirksame Methode (Diaphragma, Pessar, Portioklappe oder Latexkondom beim männlichen Partner) anzuwenden.

Verschreibungsverfahren für ANA:

Die in der Bekanntmachung der Arzneimittelkommission der Deutschen Ärzteschaft verlangten Sicherheitsanforderungen beinhalten die folgenden Maßnahmen:

- Ausführliche Aufklärung der Patientin/des Patienten
- Risiken der Therapie
- ANA darf nicht an andere Personen ab- oder weitergegeben werden.

- Während der ANA-Behandlung und mind. 1 Monat nach Therapieende darf keine Blutspende erfolgen.

Aufklärung, Einverständnis und Dokumentation:

- Aushändigung von schriftlichem Informationsmaterial an die Patientin/den Patienten über die Anwendung, die Risiken der Anwendung, die Notwendigkeit einer sicheren Kontrazeption und über geeignete Methoden zur sicheren Kontrazeption.
- Schriftliche Patienteneinwilligung in die Behandlung
- Schriftliche Bestätigung der Aufklärung durch den Patienten und Erklärung, dass die Aufklärung verstanden worden ist.
- Dokumentation von Aufklärung und Einwilligung

Verordnung von ANA:

Bei der Bereitstellung von ANA sind geltenden arzneimittelrechtlichen und apothekenrechtlichen Vorschriften sowie die Meldepflicht von Verdachtsfällen unerwünschter Arzneimittelwirkungen einzuhalten.

Erstverschreibung:

Nach der Patientenberatung wird das Formular „Patienteneinwilligungserklärung" ausgefüllt und unterschrieben. Das Formular „Patienteneinwilligungserklärung" wird vom Patienten als auch vom aufklärenden Arzt unterschrieben. Die Bereitstellung einer Kühlkette und die Belieferung von ANA an die Wohnadresse der Patienten erfolgt durch die Charité Apotheke als Studienpartner.

8.2.2. Anweisung für Dosisanpassung:

Die Beendigung der Studienmedikation kann unter den folgenden Bedingungen erforderlich werden:

- Schwerwiegendes unerwünschtes Ereignis (siehe 9.1.)
- Intoleranz gegenüber einer Dosis von 100 mg ANA
- Neutropenie (absolute Neutrophilenzahl < 1,5 x $10^{9/}$l)

8.2.2.1. Notfallmaßnahmen

In klinischen Studien bei Patienten mit rheumatoider Arthritis wurden keine dosislimitierenden Toxizitäten beobachtet. In Studien zur Sepsis erhielten 1015 Patienten Kineret in Dosierungen bis zu 2 mg/kg/Stunde über einen Behandlungszeitraum von 72 Stunden. Das Nebenwirkungsprofil aus diesen Studien unterscheidet sich insgesamt nicht von dem aus Studien zur rheumatoiden Arthritis.
Bei Medikamentenintoxikation oder Überempfindlichkeitsreaktionen ist eine Vorstellung in der Rettungsstelle des Virchow-Klinikums oder ggf. eine wohnortnahe Erstversorgung in einem Klinikum der Vollversorgung angezeigt.

8.3. Begleitmedikation

Symptomatische und supportive Therapien werden unabhängig von der ANA-Behandlung durchgeführt. Eine Wechselwirkung mit symptomatischen Therapien und supportiven Maßnahmen einschließlich der nicht-invasiven Maskenventilation oder perkutane endoskopische Gastrostomie (PEG) ist nicht zu erwarten.

8.3.1. Besondere Kennzeichnung

Die Kennzeichnung von Kineret 100 mg Fertigspritzen beinhaltet die Bezeichnung des Arzneimittels, die Darreichungsform, Dosis des Medikamentes, den Inhalt nach Stückzahl, die Art der Anwendung und den arzneilich wirksamen Bestandteil.

8.4. Lagerung, Aus- und Rückgabe sowie Dokumentation der Studienmedikation

Das Medikament wird über die kooperierende Apotheke als reguläre Handelsware erworben. Die Lagerung, Kühlkette, Belieferung, Aus- und Rückgabe erfolgt durch die kooperierende

Apotheke oder durch den Prüfarzt. Der Empfang der Medikation wird durch den Prüfarzt, den Apotheker sowie den Patienten dokumentiert. Nach Belieferung erfolgt die Aufbewahrung der Prüfmedikation in der häuslichen Umgebung des Patienten in einem Kühlschrank.

Leere Behältnisse und unverbrauchte Einheiten des Prüfmedikamentes werden vom Prüfarzt entgegengenommen. Nicht verbrauchte Prüfmedikation wird vom Prüfarzt an die kooperierende Apotheke weitergeleitet und dort vernichtet.

Die Dokumentation der Weitergabe des Prüfmedikamentes und des Verbrauchs, des Verlustes von Medikation sowie des Nichtgebrauchs und der Restbestand werden mit den folgenden Angaben dokumentiert:

- Angabe von Verordnung, Verbrauch, Nichtgebrauch oder Verlust von Prüfmedikation
- Datum der Ausgabe und des Eingangs von Prüfmedikation
- Menge der Prüfmedikation
- Kodierungsnummer der Studienpatienten (außer bei Restbeständen)

Die Prüfmedikation darf nur für Studienzwecke benutzt werden und darf auf keinen Fall außerhalb des Prüfplans Anwendung finden. Die Patienten werden verpflichtet unverbrauchte Einheiten zu jeder Visite an das Prüfzentrum zurückzubringen. Die Prüfmedikation wird vor Belieferung in der kooperierenden Apotheke aufbewahrt.

8.5. Compliance

Die Behandlung mit der Prüfmedikation erfolgt auf ambulanter Basis, so dass die folgenden Maßnahmen zur Überprüfung und Dokumentation der Compliance vorgesehen sind:

- In monatlichen Telefonvisiten und wöchentlichen Internetvisiten wird die Behandlung der Patienten überprüft.

8.6. Anforderungen an Prüfzentren und Prüfärzte:

Die einzubringende Patientenzahl von 20 Personen ist aus dem Patientenbestand am Studienzentrum sowie durch zusätzliche Rekrutierung möglich. Die Diagnose einer ALS wurde bei der überwiegenden Patientenzahl in einer auswärtigen neurologischen Klinik gestellt und nach eigener Untersuchung überprüft und bestätigt. Am Prüfzentrum sind alle diagnostischen Voraussetzungen erfüllt, um die Erst- und Bestätigungsdiagnose einer ALS zu stellen (Elektromyographie, Elektroneurographie, Liquordiagnostik, MRT-Diagnostik). Zum Studieneinschluss sind jedoch keine der genannten diagnostischen Maßnahmen erforderlich, so dass keine invasiven (Elektromyographie, Liquordiagnostik) oder bildgebenden Untersuchungen (MRT) erforderlich sind.

8.7. Aufnahme und Registrierung

8.7.1. Rekrutierungs-Logbuch

Für den Rekrutierungsvorgang wird ein Logbuch eingerichtet, in dem alle Patienten konsekutiv erfasst werden, die den Einschlusskriterien entsprechen. Dabei werden Gründe für die Nichtaufnahme in die Studie dokumentiert. Die Rekrutierung wird aus der Gruppe der bereits in Betreuung befindlichen Patienten und durch Veröffentlichung auf den Internetseiten des ALS-Studienzentrums durchgeführt. Der Vorschlag für studienbezogenen Internetpublikation befindet sich in den Anlagen. Die Internetadresse der ALS-Ambulanz des Studienzentrums lautet: www.als-charite.de.

8.7.2. Registrierung

Patienten, die alle Einschluss- und Ausschlusskriterien erfüllen und ihre Einwilligung zur Teilnahme an der Studie gegeben haben, werden mit den folgenden Daten registriert, die auf einem Registrierungsbogen dokumentiert werden. Auf dem Registrierungsbogen sind die folgenden Daten dokumentiert:

- Bezeichnung und Anschrift des Prüfzentrums
- Name des verantwortlichen Prüfarztes

- Telefon-, Fax-Nr. und E-Mail-Adresse des Prüfarztes
- Pseudonym des Patienten
- Geschlecht
- Alter
- Erkrankungsdauer (in Monaten)
- Erkrankungsart (Bulbärtyp oder Extremitätentyp)
- Affektion des 2. Motoneurons (Progressive Muskelatrophie (PMA))
- ALS-FRS
- Progressionsrate (ALS-FRS/Krankheitsverlauf [in Monaten])

8.8. Studienablauf

8.8.1. Klinische Untersuchungen und Statuserhebungen

Das Untersuchungs- und Beobachtungsprogramm beinhaltet anamnestische Erhebungen, internistisch-körperliche, neurologisch-klinische Untersuchungen, eine elektrokardiographische Diagnostik, die Bestimmung der Vitalkapazität, laborchemische Untersuchungen sowie eine Selbsterhebung der ALS-Symptomschwere. Bei Frauen im gebärfähigen Alter ist zu Beginn der Studie ein Schwangerschaftstest aus dem Blut vorgesehen. Die Untersuchungszeitpunkte und Intervalle der Präsenzvisiten, Telefonvisiten und Internetvisiten sind dem Ablaufschema in Abschnitt 4 zu entnehmen.

Bildgebende Verfahren, invasive Untersuchungen und spezielle diagnostische Verfahren mit der Notwendigkeit einer Validierung sind nicht vorgesehen.

Jede Begleitmedikation, auch außerhalb der ALS-Indikation, wird dokumentiert. Dabei sind der Behandlungsbeginn, das Medikament, die Dosierung und Indikation zu dokumentieren. Die Zwischenanamnese bei den Verlaufsuntersuchungen beinhaltet eine Medikamentenanamnese, bei der die Beendigung und Neuaufnahme jeder Form von Begleitmedikation erfasst wird.

8.8.1.1. Eingangs- und Einschlussuntersuchung

Durch eine klinisch-neurologische Untersuchung und Sichtung der zusatzdiagnostischen Untersuchungsbefunde (bereits vorliegende Daten der Elektromyographie, Elektroneurographie, Liquordiagnostik und MRT-Diagnostik) wird die Diagnose einer ALS verifiziert und eine Einschätzung der Erkrankungsschwere vorgenommen (revidierte El Escorial-Kriterien, ALS-FRS-R). Die anamnestischen klinischen Daten zum Zeitpunkt des Erkrankungsbeginns, der ALS-Verlaufsform (Dominanz des 2. motorischen Neurons oder Progressive Muskelatrophie (PMA)) und eine eigenanamnestische Abschätzung des ALS-FRS werden erfasst. Bei der Eingangsuntersuchung wird die Erfüllung der Auswahlkriterien überprüft. Außerdem werden die für die Verträglichkeitsbeurteilung relevanten Basisdaten erhoben. Der Patient erfährt eine Aufklärung über das Studienvorhaben. Die Einwilligung des Patienten wird eingeholt. Die Eingangsuntersuchung (V1) umfasst das folgende Untersuchungsprogramm:

- Anamnese
- Körpergröße und Körpergewicht
- Internistisch-körperliche Untersuchung
- Klinisch-neurologische Untersuchung
- Manuelle Muskeltestung (MMT)
- Messung von Blutdruck und Herzfrequenz
- Elektrokardiographie
- Messung der Vitalkapazität
- Schwangerschaftstest bei Frauen im gebärfähigen Alter
- Hämatologische Diagnostik: Differentialblutbild, Hämatokrit, Thromboplastinzeit, international-normierte Ratio (INR), Prothrombinzeit (PTT), Fibrinogen

- Klinisch-chemische Untersuchungen: AST, ALT, GGT, Bilirubin-gesamt, Bilirubin-konjugiert, alkalische Phosphatase, Kreatin, Harnstoff, Natrium (Na^+), Kalium (K^+), CRP
- Infektionsdiagnostik: Tuberkulin-Test (Mendel-Mantoux-Test), bei BCG-geimpften Patienten wird der Mendel-Mantoux-Test durch einen γ-Interferon-Test ersetzt, anti-HBc IgG+IgM, HBs-Antigen, anti-HIV 1+2
- Immunelektrophorese mit Immunfixation zum Ausschluss einer signifikanten monoklonalen Gammopathie
- Serumwerte von IL-1b, IL-1RA, TNF, IL-6, IL-8
- Revidierte ALS-Funktions-Bewertungsskala (*ALS Functional Rating-Scale* - ALS-FRS-R)

In der Screening-Untersuchung und den Nachfolgevisiten wird eine körperliche Untersuchung durchgeführt. Sie beinhaltet eine Inspektion und ggf. Palpation oder Auskultation des Kopfes, der Ohren und der Augen, des Schlundes, Thorax und Abdomens, der Schilddrüse, ausgewählter Lymphknoten, der Haut und Extremitäten. Die Daten über die körperliche Untersuchung werden in der Patientenakte dokumentiert. Signifikante Befunde im Studienverlauf werden im eCRF dokumentiert. Klinisch signifikante pathologische Befunde nach Einschätzung des Prüfarztes im Studienverlauf werden als unerwünschtes klinisches Ereignis definiert und als SAE gemeldet.

Die muskuläre Schwäche ist das Hauptsymptom der ALS. Die manuelle Muskeltestung (MMT) wurde als Standardmethode zur Einschätzung motorischer Defizite bei neurologischen Erkrankungen entwickelt. Der MMT ist ein international etabliertes Maß der Willkürmotorik. Der MMT erfasst insgesamt 16 Muskelgruppen, die eine Einschätzung der oberen Extremitäten (7 Muskelgruppen), der unteren Extremitäten (7 Muskelgruppen) sowie der paravertebralen zervikalen Muskelgruppen (2 Muskelgruppen) erfasst. Die Störung der Willkürmotorik wird in 6 Schweregraden (Kraftgrad 0-5) skaliert. Der MMT ergibt damit einen maximalen Gesamtscore von 150.

Die Überprüfung der Vitalparameter beinhaltet den systolischen und diastolischen Blutdruck sowie den Radialpuls (Herzfrequenz). Die Messung der Vitalparameter erfolgt in der liegenden Position nach einer Ruhezeit von mind. 3 Minuten. Die Blutdruckmessung erfolgt an einem Arm, der bei Wiederholungsmessungen wieder gewählt wird. Die Blutdruckmessung wird vor einer möglichen Blutentnahme durchgeführt.

Die Vitalkapazität (VK) wird durch eine Spirometrie bestimmt und liefert die Daten zur forcierten Vitalkapazität. Die VK dient als Maß der neuromuskulär-bedingten Hypoventilation. Die forcierte VK ist das Luftvolumen, das der Patient nach einer maximalen Inspiration durch eine forcierte und vollständige Expiration ausatmet. Die forcierte VK wird als prozentualer Wert eines standardisierten prädiktiven Wertes der VK angegeben und dokumentiert. Die Ergebnisse der Spirometrie werden ausgedruckt, so dass die Primärdaten in der Patientendokumentation (*source data*) zur Verfügung stehen und in den elektronischen Dokumentationsbogen (eCRF) übertragen werden.

Die Elektrokardiographie (EKG) erfolgt als 12-Kanal-Standardableitung. Ein Ausdruck der EKG-Ableitung wird in der Patientenakte dokumentiert. Die Interpretation und Auswertung erfolgt durch den Prüfarzt. Bei Hinweisen auf pathologische Befunde erfolgt eine kardiologische Einschätzung am Studienzentrum.

Der ALS-FRS-R ist ein strukturierter Fragenkatalog, in dem 12 Funktionsbereiche erfasst werden, die typischerweise bei der ALS beeinträchtigt werden [21]. Der ALS-FRS-R wurde für den Gebrauch bei klinischen Studien von ALS-Patienten entwickelt. Die 12 Funktionsbereiche beinhalten die Einschätzung der manuellen Funktionen, der Lauf- und Stehfunktion, der Schluckfunktion, der sprachlichen Kommunikation und der Atemfunktion. Jeder der 12 Funktionsbereiche ist in 5 Beeinträchtigungsgrade skaliert (0-4 Punkte), so dass sich ein Gesamtscore von 48 Punkten ergibt.

8.8.1.2. Verlaufsuntersuchungen

Die Visitenabfolge der Verlaufsuntersuchungen ist der tabellarischen Übersicht (siehe 4.0) zu entnehmen. Dabei ist das folgende Untersuchungsprogramm vorgesehen:

Zu den Präsenzvisiten 3 bis 7 (V3-V7)

- Zwischenanamnese einschließlich Medikamentenanamnese
- Internistisch-körperliche Untersuchung
- Klinisch-neurologische Untersuchung
- Manuelle Muskeltestung (MMT)
- Vitalparameter einschließlich Messung von Blutdruck und Herzfrequenz
- Elektrokardiographie
- Messung der Vitalkapazität
- Hämatologische Diagnostik: Differentialblutbild, Hämatokrit, Thromboplastinzeit, international-normierte Ratio (INR), Prothrombinzeit (PTT), Fibrinogen
- Klinisch-chemische Untersuchungen: AST, ALT, GGT, Bilirubin-gesamt, Bilirubin-konjugiert, alkalische Phosphatase, Kreatin, Harnstoff, Natrium (Na^+), Kalium (K^+), CRP
- Serumwerte von IL-1b, IL-1RA, TNF, IL-6, IL-8
- Revidierte ALS-Funktions-Bewertungsskala (ALS-FRS-R)
- Abfrage unerwünschter Ereignisse

Zu den Telefonvisiten 1 bis 7 (TV1-TV7)

Die Telefonvisiten finden nach Woche 16 im Abstand von 1 Monat statt. Die Telefonvisiten erfolgen durch eine Studienschwester am Studienzentrum. Dabei ist das folgende Visitenprogramm vorgesehen:

- Medikamentenanamnese
- Revidierte ALS-Funktions-Bewertungsskala (ALS-FRS-R)
- Abfrage unerwünschter Ereignisse

Zu den Internetvisiten 1 bis 51 (WebV1-WebV51)

Zur Internetvisite steht eine browserbasierte Anwendung bereit, die als Internetportal über www.ALSHome.de standardisierte Selbstbewertungen in eine Datenbank transferiert, die durch den Patienten und den Studienarzt eingesehen und in der Entwicklung verfolgt werden können. Die internetgestützte Selbstbewertung und Dokumentation der Nebenwirkungen zielt darauf ab, den Patienten die Möglichkeit zu geben, den Krankheits- und Behandlungsverlauf zwischen den Visiten zu dokumentieren, sie selbst zu verfolgen und für den Studienarzt Informationen zu generieren, die ein verbessertes Abbild von Nebenwirkungen und des Krankheitsverlaufes schaffen. Die zusätzlich erhobenen Daten sind auf der Internetplattform www.ALShome.de für den Patienten und den Studienarzt einsehbar und können im Rahmen der Präsenzvisiten unterstützend herangezogen werden. Eine Intervention basierend auf diesen Daten zwischen den Präsenzvisiten ist nicht vorgesehen. Dabei ist das folgende Visitenprogramm vorgesehen:

- Selbstbewertung mit revidierter ALS-Funktions-Bewertungsskala (ALS-FRS-R)
- Selbstbewertung von Kopfschmerzen mit Verbale Rating-Skala (VRS)
- Selbstbewertung der Reaktionen an der Einstichstelle

Die Verbale Rating-Skala (VRS) ist ein validiertes Assessmentinstrument zur Selbsteinschätzung der Schmerzintensität. Sie wird unter anderem bei der Erfassung von Kopfschmerzen verwendet. Hierbei wird die Stärke des Schmerzes in Begriffe (keine - leicht - mäßig - stark - sehr stark – unerträglich) gefasst.

Die Selbstbewertung der Reaktionen an der Einstichstelle ist eine Einschätzung der Läsion an der Hautoberfläche mittels eines festgelegten kurzen Fragebogens mit standardisierten Antworten. Die Ausprägung wird wie folgt bewertet: Rötung (Schweregrad 0-3), Schwellung (Schweregrad 0-3), Schmerzen (Schweregrad 0-3), Blutung der Haut (Schweregrad 0-3). Die

Selbstbewertung der Reaktionen an der Einstichstelle soll die Dokumentation des Studienarztes unterstützen.

8.8.1.3. Abschlussuntersuchung :

Die Abschlussuntersuchung findet am Ende der Studienteilnahme zur Visite 8 nach einer Behandlungszeit von 12 Monaten statt. Das Untersuchungsprogramm umfasst eine Beurteilung des Gesundheitszustandes des Patienten vor Entlassung aus dieser Studie, das die folgenden Untersuchungsschritte beinhaltet:

- Zwischenanamnese einschließlich Medikamentenanamnese
- Körpergröße und Körpergewicht
- Internistisch-körperliche Untersuchung
- Klinisch-neurologische Untersuchung
- Manuelle Muskeltestung (MMT)
- Messung von Blutdruck und Herzfrequenz
- Elektrokardiographie
- Messung der Vitalkapazität
- Hämatologische Diagnostik: Differentialblutbild, Hämatokrit, Thromboplastinzeit, international-normierte Ratio (INR), Prothrombinzeit (PTT)
- Klinisch-chemische Untersuchungen: AST, ALT, GGT, Bilirubin-gesamt, Bilirubin-konjugiert, alkalische Phosphatase, Kreatin, Harnstoff, Natrium (Na^+), Kalium (K^+), CRP, Fibrinogen,
- Serumwerte von IL-1b, IL-1RA, TNF, IL-6, IL-8
- Revidierte ALS-Funktions-Bewertungsskala (ALS-FRS-R)
- Abfrage unerwünschter Ereingisse

3 Monate nach Beendigung der Studie ist eine Nachuntersuchung in der Behandlungs- und Beobachtungsgruppe vorgesehen, die dem Untersuchungsprogramm der Abschlussuntersuchung in der Beobachtungsgruppe entspricht.

8.8.1.4. Laboruntersuchung

Die unter 8.8. genannten Laboruntersuchungen werden in den Labors des Instituts für Laboratoriumsmedizin und Pathobiochemie und des Instituts für medizinische Immunologie am Studienzentrum durchgeführt. Eine Untersuchung von Parametern in Speziallabors außerhalb des Klinikums ist nicht vorgesehen. Die Bewertung der Ergebnisse aus der Labordiagnostik erfolgt nach klinikeigenen Referenzbereichen (Übersicht siehe Anlage 8). Ein studienbegleitendes wissenschaftliches Begleitprogramm ist nicht vorgesehen.

8.9. Dauer der Studienteilnahme für den einzelnen Patienten:

Der Studieneinschluss erfolgt zur Visite 1. Die ANA-Behandlung umfasst 12 Monate, in deren Verlauf 7 Präsenzvisiten, 7 Telefonvisiten und 51 Internetvisiten stattfinden. Die Studienteilnahme und Behandlung mit der Prüfmedikation endet nach 12 Monaten am Tag der Visite 8. Als Nachbeobachtung ist eine Visite 3 Monate nach Abschluss der Studie vorgesehen.

8.10. Vorzeitiger Studienabbruch eines einzelnen Patienten

Ein vorzeitiges Ausscheiden eines Patienten aus der Studie ist möglich oder erforderlich, wenn mindestens eines der Abbruchkriterien vorliegt. Die folgenden Ereignisse sind als Begründung für den vorzeitigen Studienabbruch anzusehen:

- Laborparameter außerhalb des Normbereiches (z. B.):
  - Neutropenie (ANZ < 1,5 x 10^9/l)
  - Schwere Nierenfunktionsstörung (CLCr < 30 ml/Minute)
  - Erhöhung um das 5-fache des oberen Normwertes der ALT/AST-Werte
- Eintritt einer Schwangerschaft
- Signifikante Prüfplanverletzung

- Fehlende Compliance
- Schwerwiegende Infektion
- Klinische Hinweise auf eine allergische Reaktion einschließlich makulopapulöser oder urtikarieller Hautausschläge und einer unklaren Erhöhung der Körpertemperatur
- Schwerwiegende Hautreaktionen, insbesondere bullöse Hautreaktionen einschließlich des Stevens-Johnson-Syndroms und der toxisch-epidermalen Nekrolyse
- Pathologische Untersuchungsbefunde einschließlich der Elektrokardiographie
- Persönlicher Wunsch des Patienten
- Widerruf der Patienteneinwilligung
- Kontaktverlust
- Ortswechsel, der eine Einhaltung des Protokolls nicht möglich macht
- Tod des Patienten

Nach dem Ausscheiden aus der Behandlungsgruppe wird das Untersuchungsprogramm wie nach regulärem Therapieende (Visite 8) durchgeführt. Weiterhin ist eine Nachbeobachtung nach 3 Monaten vorgesehen um möglichst vollständige Daten zu erheben.

8.11. Vorzeitiger Abbruch der gesamten klinischen Prüfung

Ein vorzeitiges Studienende und Abbruch der gesamten Studie sind unter bestimmten Bedingungen möglich, die im Folgenden definiert werden:

- Ein Patient mit möglicher, wahrscheinlicher oder sicherer Kausalität einer schweren Infektion, die nicht im Kontext der Grunderkrankung zu erklären ist. Die Entscheidung wird mit dem Referenzinstitut vorgenommen. In die Entscheidung gehen maßgeblich die Schwere der Infektion und die Wahrscheinlichkeit der Kausalität ein.
- Entscheidung der Studienleitung bei unvertretbaren Risiken und Toxizitäten von ANA in Kombination mit Riluzol unter Nutzen-Risiko-Abwägung.
- Neue wissenschaftliche Erkenntnisse über ANA während der Laufzeit der Studie.

Die Entscheidung über den Abbruch der Studie wird durch die Studienleitung getroffen.

9. Unerwünschte Ereignisse

9.1. Definition unerwünschter Ereignisse (AE)

Als unerwünschtes Ereignis wird jedes medizinisch unerwünschte Ereignis (Adverse Event (AE)) definiert, das einem studienteilnehmenden Patienten widerfährt und das nicht unbedingt in einem kausalen Zusammen-hang mit der Prüfmedikation steht. Als unerwünschtes Ereignis sind Erkrankungen, Krankheitszeichen oder Symptome anzusehen, die nach Einschluss der Patienten in die Studie eintreten oder sich verschlechtern. Die Ausprägung wird wie folgt bewertet:

- leicht
- mäßig
- schwer
- lebensbedrohend

Für jedes Ereignis wird eine Kausalitätsbewertung zur Prüfmedikation vorgenommen:

- nicht beurteilbar
- ohne Zusammenhang
- möglicher Zusammenhang
- wahrscheinlicher Zusammenhang
- sicherer Zusammenhang

Die folgenden ALS-bedingten unerwünschten Ereignisse sind möglich oder zu erwarten:

- Progression der Paresen
- Stürze als Folge progredienter Gangstörung mit traumatischen Ereignissen einschließlich Frakturen

- Kachexie durch Myatrophie und Dysphagie
- Fortschreitende dysphagische Störung mit Erfordernis einer perkutanen endoskopischen Gastrostomie (PEG) einschließlich stationärer Anlage einer PEG
- Fortschreitende Hypoventilation einschließlich der Initiierung einer nicht-invasiven intermittierenden Maskenventilation (intermittierende Selbstbeatmung)
- Progrediente respiratorische Insuffizienz mit Todesfolge oder Durchführung einer Tracheotomie mit mechanischer Ventilation

Die folgenden klinischen Ereignisse sind im Zusammenhang mit der ANA-Behandlung zu erwarten oder möglich (siehe Punkt 5.1.).
- Reaktionen an der Einstichstelle
- Kopfschmerzen
- Allergische Reaktionen
- Schwerwiegende Infektionen
- Neutropenie

9.2. Definition schwerwiegender unerwünschter Ereignisse (SAE)

Als schwerwiegend ist ein unerwünschtes Ereignis zu definieren, das die folgenden Merkmale aufweist:
- Lebensbedrohlichkeit oder Tod
- Notwendigkeit einer stationären Behandlung oder deren Verlängerung
- bleibende oder schwerwiegende Behinderung oder Invalidität
- kongenitale Anomalien oder Geburtsfehler

Bekannte und zu erwartende SAE:
In direktem Bezug zur Studie sind schwerwiegende unerwünschte Ereignisse als bekannte Folge der ALS oder der ANA-Behandlung zu unterscheiden. SAE, die auf die Behandlung mit ANA zurückzuführen sind, sind als schwerwiegende unerwünschte Arzneimittelreaktion (Serious Adverse Reaction (SAR)) zu werten. Das bedeutendste SAR in Verbindung mit ANA ist die schwerwiegende Infektion. Weitere ANA-assoziierte SAR sind allergische Reaktionen und eine Neutropenie, deren Wahrscheinlichkeit außerhalb einer onkologischen Indikation als selten zu betrachten ist. Diese SARs sind entsprechend Kap. 9.6 zu melden.

SAE im Zusammenhang mit der Grunderkrankung:
Im Zusammenhang mit der ALS sind SAE mit Lebensbedrohung oder Todesfolge zu erwarten, da die ALS eine einheitlich fortschreitende und fatal verlaufende Erkrankung darstellt. Im natürlichen Verlauf der Erkrankung ergibt sich eine fortschreitende neuromuskulär bedingte Hypoventilation, die zu einer respiratorischen Insuffizienz führt. Bei der Mehrheit der Patienten führt die respiratorische Insuffizienz zum Tode, während ein Teil der Patienten sich für eine stationäre Behandlung, Durchführung einer Tracheotomie und mechanische Ventilation entscheidet. Häufige SAE im Zusammenhang mit der ALS sind als Folge der progredienten Dysphagie zu erwarten, die zu einer stationären Behandlung und Anlage einer perkutanen endoskopischen Gastrostomie (PEG) führt. Andere ALS-bedingte SAE bestehen in der stationären Behandlung von traumatologischen Folgen der Paresen und Stürze. Diese SAEs, die im Zusammenhang mit der Grunderkrankung stehen, werden als AEs dokumentiert, sind jedoch ausgenommen von der unten aufgeführten Meldepflicht an den Sponsor.

9.3. Beurteilung der Intensität von unerwünschten Ereignissen:

Die folgenden Intensitätsmerkmale unerwünschter Ereignisse sind zu differenzieren:
- Leicht: Das unerwünschte Ereignis ist vorübergehend und vom Patienten leicht zu tolerieren.
- Mäßig: Das unerwünschte Ereignis bereitet dem Patienten Unannehmlichkeiten und eine Behinderung bei üblichen Tätigkeiten.

- Schwer: Das unerwünschte Ereignis bereitet dem Patienten erhebliche Störungen seiner üblichen Aktivitäten.
- Lebensbedrohend: Klinische Situationen, die unverzügliche Interventionen notwendig machen

9.4. Beurteilung des Kausalzusammenhangs

Für die Beurteilung des Zusammenhangs zwischen der Anwendung von ANA in Kombination mit Riluzol bei der ALS und dem Auftreten von unerwünschten Ereignissen sowie schwerwiegenden unerwünschten Ereignissen werden folgende Definition verwendet:

- *Sicherer Zusammenhang:*

Eine Reaktion, die in einem nachvollziehbaren zeitlichen Ablauf nach der Anwendung von ANA in Kombination mit Riluzol folgt, einem bekannten oder erwarteten Antwortmuster auf das Prüfprodukt ANA folgt und nach Absetzen oder Dosisreduktion regredient ist und bei erneuter Exposition wieder auftritt.

- *Wahrscheinlicher Zusammenhang:*

Eine Reaktion, die einem nachvollziehbaren Ablauf nach der Anwendung von ANA folgt, einem bekannten oder erwarteten Antwortmuster auf das Prüfprodukt ANA folgt und nach Absetzen oder Dosisreduktion regredient ist und nicht durch die bekannten Merkmale der ALS oder anderer bekannter klinischer Zustände des Patienten erklärt werden kann.

- *Möglicher Zusammenhang:*

Eine Reaktion, die einem nachvollziehbaren zeitlichen Ablauf nach Anwendung von ANA folgt, einem bekannten oder erwarteten Antwortmuster auf das Prüfprodukt ANA folgt, die aber leicht auch durch eine Reihe anderer Faktoren hervorgerufen werden könnte.

- *Ohne Zusammenhang:*

Eine Reaktion, bei der ausreichende Informationen für die Annahme vorliegen, dass kein Zusammenhang mit ANA in Kombination mit Riluzol besteht.

- *Nicht beurteilbar:*

Eine Einschätzung des Zusammenhangs zwischen dem unerwünschten Ereignis und der Prüfmedikation ist nicht möglich.

9.5. Dokumentation unerwünschter Ereignisse:

Jedes unerwünschte Ereignis wird im eCRF und in der Patientenakte dokumentiert.
Die Dokumentation umfasst die Art des Ereignisses, den Beginn, die Dauer, den Schweregrad, die getroffenen Maßnahmen zur Behandlung, die Einschätzung der Kausalität und den Ausgang des Ereignisses. In Zusammenhang stehende Krankheitszeichen und Laborwertveränderungen werden, nach eingehender Überprüfung durch den Prüfarzt, syndromatisch zusammengefasst und einer einzigen Erkrankung zugeordnet. Labordaten, die außerhalb des Normbereiches liegen, werden vom Prüfarzt hinsichtlich ihrer klinischen Bedeutung bewertet und bei entsprechender Relevanz ebenfalls als unerwünschtes Ereignis erfasst und entsprechend ihrer Bewertung gemeldet. Alle unerwünschten Ereignisse sind bis zur Regredienz oder Stabilisierung zu verfolgen.

Der Sponsor dokumentiert ausführlich alle ihm von den Prüfärzten mitgeteilten unerwünschten Ereignisse. Diese Aufzeichnungen übermittelt der Sponsor der zuständigen Bundesoberbehörde und den zuständigen Behörden anderer Mitgliedstaaten der EU und anderen Vertragsstaaten des Abkommens über den EWR, in deren Hoheitsgebiet die Studie durchgeführt wird, auf Anforderung.

9.6. Meldung schwerwiegender und/oder unerwünschter Ereignisse

Der Prüfarzt meldet jedes nach Therapiebeginn (Visite 2) aufgetretene schwerwiegende unerwünschte Ereignis nach Bekanntwerden innerhalb von 24 Stunden und jedes

medizinisch relevante unerwünschte Ereignis (z. B. Laborwerte (Neutropenie), Ausschlusskriterien) innerhalb von 7 Tagen unter Verwendung des SAE-Meldebogens an das KKS Charite. Die Meldung erfolgt per Fax an die Nummer +49-30-450-565919. Wenn die erforderlichen Informationen zu diesem Zeitpunkt nicht verfügbar sind, müssen Folgeberichte erstellt werden. Bei Todesfällen wird nach Möglichkeit eine Kopie des Autopsiebefundes beigelegt.

Jedes SAE wird einer unabhängigen Zweitbewertung durch einen vom Sponsor benannten Medical Monitor unterzogen. Die Einschätzung der Kausalität, die vom Prüfarzt getroffen wurde, hat die höhere Priorität.

Im Rahmen dieser Studie sollten die folgenden schwerwiegenden Ereignisse von der Meldepflicht ausgenommen werden, da sie einheitlich im natürlichen Verlauf der ALS zu erwarten sind (vgl. auch 9.2):

- Stationäre Behandlung zur Adaptation einer nicht-invasiven Maskenventilation (intermittierende Selbstbeatmung)
- Stationäre Behandlung zur Anlage einer perkutanen endoskopischen Gastrostomie (PEG)
- Progression von Extremitätenparesen
- Schwerwiegende oder unerwartete Ereignisse, die nach der Registrierung, jedoch vor Behandlungsbeginn eintreten

9.7. Follow-ups von SAE

Sämtliche SAE müssen bis zum Ausgang/Klärung des SAE verfolgt werden, bzw. bis eine Erklärung der Ursache vorliegt oder kein Kontakt mehr zum Patienten möglich ist. Der Prüfarzt ist dafür verantwortlich, dass die nötigen Informationen zur Erklärung und Befundung des SAE ergänzt werden. Dazu muss zu jedem nicht abgeschlossenem SAE ein Follow-up Bericht innerhalb von 24h nach Bekanntwerden bzw. beim Eintreten neuer Umstände an das KKS Charité geschickt werden. Dazu wird ein neuer SAE-Bogen verwendet, auf dem das Datum der ursprünglichen SAE-Meldung vermerkt wird.

9.8. Meldung von Verdachtsfällen unerwarteter schwerwiegender Nebenwirkungen (Suspected Unexpected Serious Adverse Reaction, SUSAR)

Definition: SUSAR (Suspected Unexpected Serious Adverse Reaction) sind Verdachtsfälle von unerwarteten schwerwiegenden Nebenwirkungen, mit folgenden Kriterien:

- Art oder Schweregrad stimmen nicht mit den, im Referenzdokument (Investigator’s Brochure) genannten Information über das Prüfpräparat überein (GCP-V §3 (9).
- Es muss ein schwerwiegendes unerwünschtes Ereignis vorliegen. Die folgenden unerwünschten Ereignisse gelten als schwerwiegend:
  - Lebensbedrohlich oder tödlich
  - Stationäre Behandlung erforderlich oder verlängert
  - Bleibende oder schwerwiegende Behinderung oder Invalidität
  - Geburtsfehler oder kongenitale Anomalie
- Es muss ein ursächlicher Zusammenhang zwischen der Verabreichung des Prüfpräparates und dem Ereignis bestehen (nachteilige und unbeabsichtigte Reaktion auf ein Prüfpräparat, unabhängig von dessen Dosierung, GCP-V § 3 (7).

Der Sponsor meldet jeden ihm bekannt gewordenen Verdachtsfall einer unerwarteten schwerwiegenden Nebenwirkung (SUSAR) unverzüglich, spätestens aber innerhalb von 15 Tagen nach Bekanntwerden, der zuständigen Ethikkommission, der zuständigen Bundesoberbehörde und den zuständigen Behörden anderer Mitgliedstaaten der EU und anderen Vertragsstaaten des Abkommens über den EWR, in deren Hoheitsgebiet die Studie durchgeführt wird. Weiterhin unterrichtet er alle an der Studie beteiligten Prüfärzte.

Der Sponsor übermittelt bei einem SUSAR, welches zu einem Todesfall geführt hat oder lebensbedrohlich ist, unverzüglich, spätestens aber innerhalb von 7 Tagen nach Bekanntwerden, der zuständigen Ethikkommission, der zuständigen Bundesoberbehörde und den zuständigen Behörden anderer Mitgliedstaaten der EU und anderen

Vertragsstaaten des Abkommens über den EWR, in deren Hoheitsgebiet die Studie durchgeführt wird, sowie allen beteiligten Prüfärzten alle für die Bewertung wichtigen Informationen und innerhalb von höchsten 8 weiteren Tagen die weiteren relevanten Informationen.

Der Sponsor unterrichtet unverzüglich, spätestens aber innerhalb von 15 Tagen nach Bekanntwerden, die zuständige Ethikkommission, die zuständige Bundesoberbehörde und die zuständigen Behörden anderer Mitgliedstaaten der EU und anderen Vertragsstaaten des Abkommens über den EWR, in deren Hoheitsgebiet die Studie durchgeführt wird, über jeden Sachverhalt, der eine erneute Überprüfung der Nutzen-Risiko-Bewertung des Prüfpräparates erfordert. Hierzu gehören insbesondere:

- Einzelfallberichte von erwarteten schwerwiegenden Nebenwirkungen mit einem unerwarteten Ausgang
- Erhöhung der Häufigkeit erwarteter schwerwiegender Nebenwirkungen, die als klinisch relevant bewertet wird
- SUSARs, die sich ereigneten, nachdem die betroffene Person die klinische Prüfung bereits beendet hat (3 Monate nach Studienende/-ausschluss)
- Ereignisse im Zusammenhang mit der Studiendurchführung oder der Entwicklung des Prüfpräparates, die möglicherweise die Sicherheit der betroffenen Personen beeinträchtigen können

Personenbezogene Daten werden vor ihrer Übermittlung immer pseudonymisiert.

9.9. Schwangerschaft

Das Auftreten einer Schwangerschaft bei einer Patientin oder aber der Partnerin eines Patienten während der Studienteilnahme und bis zu 42 Tagen nach der Verabreichung der letzten Injektion des Prüfpräparates muss **umgehend** an das KKS Charité gemeldet werden. Eine schwangere Patientin wird sofort von der Studie ausgeschlossen.

Der Verlauf der Schwangerschaft muss bis zum Ende verfolgt werden. Dies beinhaltet eine spontane oder freiwillige Beendigung, Details der Geburt, das Vorhandensein oder Fehlen von Geburtsfehlern, Missbildungen oder Komplikationen beim Neugeborenen und/oder der Mutter.

Die Dokumentation erfolgt auf dem Schwangerschaftsberichtsbogen, der an das KKS Charité gefaxt wird (s.o.). Für die Nachverfolgung der Schwangerschaft ist von der Patientin eine gesonderte Einwilligungserklärung einzuholen, die es erlaubt, Informationen über den Verlauf und den Ausgang der Schwangerschaft zu berichten. Nach der Geburt des Kindes muss für die Nachverfolgung des Gesundheitszustandes eine Einwilligung der Sorgeberechtigten eingeholt werden.

9.10. Ansprechpartner für die Meldung von SAE:

Rita Pilger
Abteilung Pharmakovigilanz
Koordinierungszentrum für Klinische Studien Charité
Augustenburger Platz 1, 13353 Berlin
Tel.: 030.450-553872; 030.450-553016 (Sekretariat)
Fax: 030.450-553937
E-Mail: rita.pilger@charite.de

10. Dokumentation

10.1 Dokumentationsbögen (CRF)

Die Datenerfassung erfolgt durch elektronische Dokumentationsbögen (eCRF) durch eine Studiensoftware, die durch das KKS zur Verfügung gestellt wird.

10.2. Prüfarztordner

Im Prüfarztordner werden alle essentiellen Dokumente gemäß ICHGCP Kapitel 8 im Prüfzentrum vor Ort abgelegt.

10.3. Internetportal

Die Datenerhebung der Internetvisiten erfolgt durch elektronische Dokumentation über ein Internetportal (www.ALSHome.de) und einer dazugehörigen Datenbankstruktur. Diese Quelldaten werden durch eine Reportfunktion in ein PDF-Dokument umgewandelt und stehen damit als Quelldokument zur Verfügung. Die Dateneingabe erfolgt ausschließlich patientenseitig bzw. durch eine bevollmächtigte Person. Eine hohe Datenqualität wird unter anderem dadurch erreicht, dass innerhalb der elektronischen Patientendatenbank die Eingabewerte bereits weitgehend auf Plausibilität und zulässige Wertebereiche geprüft werden. Fehleingaben werden vom System automatisiert erkannt.

11. Qualitätsmanagement

11.1 Überwachung des Studienablaufs und der Datenqualität

1.1.1.1 Monitoring

Der verantwortliche Monitor wird aus dem Koordinierungszentrum für klinische Studien (KKS) mit Sitz am Campus Virchow-Klinikum der Charité Universitätsmedizin Berlin bestellt. Im Rahmen des Monitorings werden Aufgaben zur Qualitätssicherung und Kontrolle des Studienablaufes sowie der Datenqualität wahrgenommen. Die Visiten des Monitors während der klinischen Studie finden vor Ort statt, um die Vollständigkeit der Dokumentation und der Dateneingabe in die Patientendokumentationsbögen (eCRF) sowie die Einhaltung des Prüfplanes und der GCP-Richtlinien zu kontrollieren. Weiterhin wird der Zeitplan der klinischen Studie einschließlich der Patientenrekrutierung überprüft. Die verantwortlichen Prüfärzte müssen bei der Visite des Monitors verfügbar sein und die Arbeit des Monitors vor Ort unterstützen. Weitere Aufgaben des Monitorings umfassen die Kontrolle der Auswahlkriterien, die Kontrolle der prüfplangemäßen Behandlung und Einhaltung der Untersuchungs- und Bewertungstermine.

Der Zugang des Monitors zu den Studienunterlagen wird in der Prüfervereinbarung zugesichert und der Patient stimmt dem durch seine Unterschrift auf der Einwilligungserklärung zu.

11.1.2. Audit

Die Einbeziehung in ein Audit-Programm ist nicht vorgesehen.

11.2. Standardisierung und Validierung

Die Studie wird monozentrisch durchgeführt, so dass eine spezifische Standardisierung nicht erforderlich ist. Alle eingesetzten Messmethoden und Bewertungskriterien sind als validiert einzuschätzen. Die klinisch-chemischen und hämatologischen Laboruntersuchungen erfolgen innerhalb des Studienzentrums, für die eine Validierung und Zertifizierung der durchführenden Abteilung vorliegt (Übersicht über Referenzwerte in der Anlage). Die Bestimmung der Vitalkapazität (VK) erfolgt nach einer gewichtsadaptierten Normierung. Die Bewertungskriterien des ALS-Schweregrades (ALS-FRS) sind validiert. Der ALS-FRS ist ein international etablierter Bewertungsstandard. Die Bewertungskriterien des Kopfschmerzes erfolgen über die validierte Verbale Rating-Skala (VRS). Die VRS ist ein etablierter Bewertungsstandard des Kopfschmerzes.

11.3. Referenzinstitutionen

Die Einbeziehung einer Referenzinstitution ist nicht regelhaft erforderlich und vorgesehen. Eine unabhängige Überprüfung oder Bewertung kritischer Daten durch die Referenzinstitution ist nur dann geplant, falls bei einer Häufung unerwünschter klinischer Ereignisse eine Risiko-Nutzen-Abwägung durch den Leiter der klinischen Prüfung nicht abschließend erreicht werden kann. Als Gutachter ist die folgende Institution vorgesehen:

Universitätsklinikum Ulm, Neurologische Klinik
Prof. Dr. med. Albert C. Ludolph
Direktor der Neurologischen Klinik
Steinhövelstr. 9
89075 Ulm

Die Referenzinstitution verfügt über eine beratende Funktion, um einen vorzeitigen Studienabbruch eines einzelnen Patienten oder den vorzeitigen Abbruch der gesamten klinischen Prüfung zu entscheiden.

12. Dateneingabe und Datenmanagement

Alle patientenbezogenen Daten werden in pseudonymisierter Form erfasst. Jeder Patient ist durch eine Patientennummer unverwechselbar gekennzeichnet, die bei der Registrierung zugewiesen wird. Der Prüfarzt führt eine vertrauliche Patientenliste, in der die Kenndaten mit dem vollen Patientennamen verbunden sind. Die folgenden Personen haben Zugriff auf die vertrauliche Patientenliste:

- Prüfärzte
- Studienschwestern
- Monitore

Dateneingabe und Datenmanagement der Internetvisiten

Die Bestimmungen des Datenschutzgesetzes werden für das Internetportal www.ALShome.de berücksichtigt und kommen vollständig zur Anwendung. Die online-Befragung mittels Internetportal ist eine browserbasierte Anwendung, die zur Gewinnung von Selbstbewertungen dient und in keinem direkten Patientenkontakt steht. Das Datenschutzkonzept des Internetportals, welches in der Studie genutzt werden soll, wurde vom Datenschutzbeauftragten der Charité geprüft und positiv begutachtet.

12.1. Datenerhebung und Dokumentationsbögen

Die Datenerhebung/-eingabe erfolgt ausschließlich durch internetbasierte elektronische Dokumentationsbögen (*electronic case report forms* - eCRF). Alle Daten sind vollständig pseudonymisiert (Details hierzu siehe 12.2 Datenverarbeitung).

Sämtliche Zugänge zur Studiensoftware mit Zuweisung gewünschter Rollen (z.B. Studienzentrale, Prüfarzt bzw. Dateneingabe, Monitor) werden nur durch die Studienzentrale beim KKS beantragt.

Der Prüfarzt bewahrt die medizinischen Primärdaten (*source document*) einschließlich der Labordaten, des Elektrokardiogramms, der Spirometriebefunde und anderer zuatzdiagnostischer Daten im Original am Studienzentrum auf. Alle Informationen des eCRF müssen sich auf das Primärdokument (Patientenakte) mit zusatzdiagnostischen Befunden zurückführen lassen. Die folgenden Dokumente werden bis 10 Jahre nach Abschluss der klinischen Studie am Studienzentrum aufbewahrt:

- Ethikvotum und Genehmigung durch die zuständige Bundesoberbehörde zur Durchführung der klinischen Studie
- Patientenakte und zusatzdiagnostische Befunde (*source data*)
- Kopien des Patientendokumentationsbogens (eCRF) in Druckform auf elektronischen Medien

- Patienteninformation und unterschriebene Einwilligungserklärung

Die Studiensoftware wurde am KKS etabliert und validiert. Die Software entspricht den Anforderungen der Datensicherheit. Das Programm führt eine automatische Validierung der Dateneingabe durch und überprüft die Dateneingabe hinsichtlich Diskrepanz und fehlender Daten. Die Datenübertragung erfolgt über eine sichere Internetverbindung vom Studienzentrum an das KKS. Kommt eine internetbasierte Datenübertragung nicht zustande, sind die elektronischen Daten zunächst in einem papierbasierten Dokument (eCRF-Eingabemasken als PDF-Dokument verfügbar) aufzunehmen, um nach dem Wiederherstellen des elektronischen Systems eine rasche Überleitung der Daten zu gewährleisten. Der Monitor wird systematisch den eCRF hinsichtlich Vollständigkeit und Richtigkeit der Dateneingabe überprüfen. Der verantwortliche Mitarbeiter des Monitorings wird bei formalen und inhaltlichen Fehlern der Datenerfassung den Prüfarzt instruieren, um eine Korrektur der Datenerfassung vorzunehmen. Bei den geplanten Monitorbesuchen im Studienzentrum wird eine vergleichende Überprüfung zwischen Primärdaten (*source data*) und den elektronischen Dokumentationsbögen (eCRF) vorgenommen.

Die Patientendokumentation ist mit Tinte oder Kugelschreiber vorzunehmen; Bleistifteintragungen sind nicht zulässig. Korrekturen sind wie folgt vorzunehmen: Der falsche Eintrag wird mit einer einfachen Linie durchgestrichen, die korrekte Information daneben eingetragen und vom Prüfarzt signiert sowie mit Datum versehen.

12.2. Datenverarbeitung

Sämtliche pseudonymisierten Studiendaten werden im RDE-Verfahren (RDE: Remote Data Entry) mit der Studiensoftware secuTrial® erfasst. Der Zugang zur Studiensoftware, die vollständig internetbasiert arbeitet, wird über Authentifizierungsprozeduren, ein Rollenmanagement sowie über verschlüsselte Verbindungen gesichert. Das Hosting der Studiendaten, einschließlich täglicher Datensicherung, erfolgt im KKS Charité.

Die Dateneingabe erfolgt zeitnah mit der Datenerhebung. Eine hohe Datenqualität wird u. a. dadurch erreicht, dass innerhalb der eCRF die Eingabewerte bereits weitgehend auf Plausibilität und erwartete/zulässige Wertebereiche geprüft werden. Vom System erkannte Fehleingaben können/müssen sofort durch den Prüfer korrigiert werden. Ein integriertes Dialogsystem zwischen Monitor, Prüfer und Studienzentrale trägt zusätzlich dazu bei. Sämtliche Einträge und Datenänderungen und Datenkorrekturen werden in einem AuditTrail dokumentiert.

Nach Beendigung der Studie werden die Daten (alle Studiendaten plus Metainformationen, wie Angaben zu den beteiligten Prüfzentren, Personen, Rollen und den kompletten AuditTrail) exportiert (SAS- und CDISC-Format) und mittels Statistiksoftware SAS® (SAS Institute Inc., USA) vollständig auf Plausibilität und Konsistenz geprüft. Nicht plausible oder fehlende Daten werden nach Rücksprache mit dem Prüfarzt korrigiert bzw. ergänzt. Die Datenbank wird anschließend als „geschlossen“ erklärt.

Die elektronische Dokumentation enthält alle aus der Studiensoftware exportierten Dateien, sämtliche SAS-Scripte und Datenänderungsprotokolle sowie die „geschlossene“ Datenbank.
Die biometrische/statistische Auswertung der Studiendaten erfolgt ebenfalls in SAS®.

12.3. Generierung des Pseudonyms

Die Studiensoftware generiert ein zufälliges Pseudonym, welches aus drei Buchstaben und 3 Ziffern besteht und keinen erkennbaren Zusammenhang zum Patienten aufweist.
Eine Patientenzuordnung ist nur über die Unterlagen des Arztes möglich. Bei Aufnahme des Patienten in die Studie wird ein Datenblatt mit den Personendaten (Name, Vorname, Geschlecht, Geburtsdatum) und dem zugewiesenen Pseudonym gedruckt und nicht gespeichert.

13. Statistische Analyse

13.1. Fallzahlabschätzung

Hauptziel dieser Pilotstudie ist der Nachweis der Verträglichkeit durch eine Untersuchung der Häufigkeit des Auftretens von Nebenwirkungen (Anzahl und schwere unerwünschter Ereignisse, schwerwiegender unerwünschter Ereignisse, unerwünschter Arzneimittelwirkungen, unerwarteter unerwünschter Arzneimittelwirkungen sowie schwerwiegender unerwünschter Arzneimittelwirkungen und pathologischer Laborparameter). Diese Pilotstudie wird mit N = 20 Patienten durchgeführt.

13.2. Statistische Auswertung

13.2.1 Hypothesen

In einer offenen Verträglichkeitsstudie soll überprüft werden, ob Anakinra (ANA) in einer subkutanen Injektionsdosis von 100 mg ANA in Kombination mit Riluzol eine Verträglichkeit bei Patienten mit ALS aufweist.

13.2.2. Definition der Auswertepopulation

Die Auswertung erfolgt in der intent-to-treat-Population.

13.2.3. Auswertung primärer und sekundärer Zielparameter

Alle Zielgrößen (primäre und sekundäre Endpunkte) werden zunächst explorativ untersucht und deskriptiv ausgewertet.

Sekundäre Endpunkte werden je nach Skalierung und Verteilungstyp der erfassten Daten mit parametrischen Tests oder verteilungsunabhängigen Verfahren geprüft.

Die biometrische Auswertung erfolgt in SAS® durch das KKS Charité.

13.2.4. Auswertung der Sicherheit

Der primäre Endpunkt betrifft die Sicherheit und Verträglichkeit von ANA in Kombination mit Riluzol bei Patienten mit ALS. Die folgenden Endpunkte sind vorgesehen, die eine Sicherheitsanalyse gestatten:

- Eine Untersuchung der Anzahl und Schwere von Infektionen
- Anzahl der Patienten, die eine kontinuierliche nicht-invasive Ventilation oder tracheostomagestützte invasive Ventilation benötigen.
- Die Ermittlung der Überlebenszeit oder des Zeitpunktes einer Tracheotomie durch respiratorische Insuffizienz (ausgenommen elektive Tracheotomie oder Tracheotomie zur Sekretkontrolle).

Die Einschätzung der Arzneimittelsicherheit beruht in erster Linie auf der Frequenz unerwünschter Ereignisse und der Überschreitung von Referenzwerten von Laborparametern. Andere Sicherheitsdaten gehen auf die internistisch-neurologische Untersuchung, die Ermittlung der Vitalparameter, der forcierten Vitalkapazität und die EKG-Ableitung zurück. Unerwünschte Ereignisse werden als absolute und prozentuale Häufigkeit für jede Art des klinischen Ereignisses separat ausgewiesen. Dabei wird eine Zuordnung der unerwünschten Ereignisse zu einzelnen Organsystemen vorgenommen. Die UE werden hinsichtlich des Schweregrades und der Kausalität zur Prüfmedikation klassifiziert. Pathologische Laborparameter werden mit den folgenden Daten ausgewiesen: Absolutwert, Abweichung vom Referenzwert, mittlere Laborwerte, Median, Standardabweichung, Anzahl der Patienten mit pathologischen Laborwerten. Klinisch-relevante Laborwertabweichungen werden gesondert gekennzeichnet.

13.2.5. Mögliche Zwischenauswertungen und vorzeitiger Studienabbruch

Eine Zwischenauswertung ist für 05-2011 vorgesehen. Bei einer Häufung klinisch-signifikanter unerwünschter klinischer Ereignisse ist eine Entscheidung über die Fortführung der Studie vorgesehen. Die Zwischenauswertung erfolgt durch das KKS Charité.

13.2.6. Auswertemethodik

siehe 13.2.3

14. Berichterstattung

14.1. Protokolle

Zur Dokumentation des Fortgangs und der Entwicklung der Studie werden Protokolle von allen stattfindenden Treffen der Gremien verfasst (Studienzentrum, Monitoring, Biometrie).

14.2. Prüfbericht

Die statistische Auswertung und die Erstellung eines integrierten Abschlussberichtes wird vom Sponsor bzw. vom Vertreter des Sponsors in Zusammenarbeit mit dem KKS durchgeführt. Alle enthaltenen Informationen des Berichtes sind vertraulich.

14.3. Abschlussbericht und Publikation

Nach Abschluss der biometrischen Auswertung wird ein integrierter Bericht vom Sponsor bzw. Vertreter des Sponsors erstellt. Der Bericht wird unterschrieben vom Studienleiter, dem Biometriker des KKS sowie dem verantwortlichen Monitor. Die Ergebnisse der klinischen Studie werden durch eine Publikation veröffentlicht, die den Standards des Konsort-Statements aus dem Jahre 2001 entspricht. In der vorgesehenen Publikation werden die folgenden Informationen berichtet:

- Gesamtinzidenz unerwünschter Ereignisse
- Art, Dauer, Schweregrad, Ausgang und Kausalität der unerwünschten Ereignisse
- Laborparameter

Die Veröffentlichung der Studienergebnisse ist unabhängig von den Ergebnissen nach Abschluss der klinischen Studie geplant. Die Autoren sind der Vertreter des Sponsors, die Prüfärzte und die verantwortlichen Mitarbeiter des KKS. Eine Veröffentlichung von Zwischen- und Teilergebnissen ist nicht vorgesehen.

15. Ethische, rechtliche und verwaltungstechnische Aspekte

15.1. Rechtliche Voraussetzungen für die Studie

Votum der Ethikkommission (gem. AMG § 42 (1) und GCP-V § 7)

Prüfplan, Patienteninformation und Einwilligungserklärung werden der zuständigen Ethikkommission (Landesamt für Gesundheit und soziales Berlin) zur Begutachtung vorgelegt. Die Studie wird erst nach Erhalt des zustimmenden Votums begonnen.
Die Ethikkommission wird (vom Sponsor) über alle Änderungen im Prüfplan (gem. GCP-V § 10) und über alle Ereignisse, die die Sicherheit der Patienten beeinträchtigen könnten, umgehend informiert. Ferner wird die Ethikkommission über alle dem Sponsor bekannt gewordene Verdachtsfälle von unerwarteten schwerwiegenden Nebenwirkungen sowie über das reguläre oder vorzeitige Ende der Studie unterrichtet.
Die Prüfärzte müssen bei der für sie zuständigen Ethikkommission angemeldet sein (Einreichen der Qualifikationsnachweise), bevor sie Patienten in die Studie aufnehmen. Es ist erforderlich, die Ethikkommission über Prüfplanänderungen (gem. GCP-V § 10) zu informieren.

Genehmigung der Bundesoberbehörde (gem. AMG § 42 (2) und GCP-V § 7)

Die Studie wird der zuständigen Bundesoberbehörde (BfArM) zur Genehmigung vorgelegt. Mit der Studie wird erst dann begonnen, wenn diese Genehmigung vorliegt.

Meldung bei den Landesbehörden (gem. AMG § 67)
Die Durchführung dieser Studie wird den zuständigen Behörden (für Berlin: LaGeSo) gemeldet. Der Sponsor und sämtliche Prüfärzte sind dort namentlich zu nennen

15.2. Patienteninformation und Einverständniserklärung

Aufklärung der Patienten:
Vor Aufnahme in die Studie wird jeder Patient vom Prüfarzt über das Wesen, die Ziele, die zu erwartenden Vorteile und mögliche Risiken der Studie informiert.

Einwilligung zur Studienteilnahme:
Jeder Patient muss eine schriftliche Einwilligung zur Teilnahme an der Studie erklären. Dem Patienten/der Patientin muss dabei ausreichend Zeit und Gelegenheit gegeben werden, um vor der Erklärung von Studienmaßnahmen über seine Teilnahme zu entscheiden und offene Fragen zu klären. Die Einwilligungserklärung wird vom Patienten und vom Prüfarzt unterzeichnet. Ist der Patient einwilligungsfähig, aber aufgrund der motorischen Symptome der ALS nicht in der Lage, eigenhändig zu unterschreiben, muss ein Zeuge die erfolgte mündliche Aufklärung durch Unterschrift bestätigen. Die Teilnahme nicht einwilligungsfähiger Patienten ist nicht vorgesehen. Die Patienteninformation und Einwilligungserklärung liegen in 2-facher Ausfertigung vor. Ein Exemplar verbleibt beim Prüfarzt, während das andere Exemplar dem Patienten ausgehändigt wird.

15.3. Patientenversicherung

Für die vorliegende klinische Prüfung besteht eine Probandenversicherung für klinische Studien mit HDI Gerling Industrie Versicherung AG, Am Schönenkamp 45, 40599 Düsseldorf. Es handelt sich um eine Probanden-Jahresversicherung für eine Universitätsklinik, die durch den folgenden Versicherungsmakler vermittelt wird: Ecclesia mildenberger HOSPITAL GmbH, Klingenbergstr. 4, 32754 Detmold. Die Versicherungs-schein-Nr. lautet: 5701032603017.

15.4. Datenschutz und Schweigepflicht

Die Patienten werden darüber informiert, dass ihre krankheitsbezogenen Daten in pseudonymisierter Form gespeichert und für wissenschaftliche Auswertungen verwendet werden. Die Patienten haben das Recht, über die gespeicherten Daten informiert zu werden.

15.5. Aufbewahrung der Daten

Die Originale aller zentralen Studiendokumente einschließlich der Dokumentationsbögen werden beim Sponsor für mind. 10 Jahre nach Fertigstellung des Abschlussberichtes aufbewahrt. Der Leiter der klinischen Prüfung bewahrt die administrativen Dokumente (Korrespondenz mit der Ethik-Kommission und den Überwachungsbehörden), die Patientenidentifikationsliste, die Einwilligungserklärung, Kopien der Dokumentationsbögen und der allgemeinen Studiendokumentation (Prüfplan, Amendments) für den genannten Zeitraum auf. Die Originaldaten der Studienpatienten (Krankenakten) sind entsprechend der am Prüfzentrum geltenden Archivierungsfristen aufzubewahren, während die Mindestarchivierungsfrist 15 Jahre beträgt.

15.6. Gesetzliche Grundlagen

Die Grundsätze für die ordnungsgemäße Durchführung der klinischen Prüfung von Arzneimitteln (Bundesanzeiger Nr. 243 vom 30.12.1987), die Bestimmungen des Deutschen Arzneimittelgesetzes (AMG 76, zuletzt geändert im Jahr 2004) und die Arzneimittel-prüfrichtlinien (1999) werden eingehalten. Das Protokoll wird dem Bundesinstitut für Arzneimittel und Medizinprodukte (BfArM) vorgelegt. Die Anzeige der Studie bei den zuständigen Überwachungsbehörden wird durch das Koordinierungszentrum für klinische Studien (KKS) im Auftrag des Sponsors vorgenommen.

15.7. GCP

Die Empfehlungen der guten klinischen Praxis, gültig seit dem 17.01.1997 werden berücksichtigt (siehe ICH-GCP: *International Conference on Harmonisation* – Good Clinical Practice).

15.8. Finanzierung der Studie

Die Finanzierung der Studie erfolgt durch den Sponsor. Die Prüfmedikation wird durch den Hersteller, die Swedish Orphan Biovitrum AB für die Gesamtzahl von 20 Patienten und die geplante Studiendauer von 12 Monaten zur Verfügung gestellt. Die entstehenden personellen, administrativen und logistische Kosten sowie die Verbrauchsmittel werden durch den Sponsor bereitgestellt.

15.9. Einhaltung des Protokolls und der Protokolländerungen

Der Prüfplan ist genau einzuhalten. Jede vom Prüfarzt zu vertretende Abweichung von den vorgesehenen Untersuchungs- und Behandlungsmaßnahmen und Veränderungen des Zeitplanes sind zu dokumentieren und zu begründen (z.B. Notfallmaßnahmen). Prüfplanänderungen oder Ergänzungen zum Prüfplan können nur vom Leiter der klinischen Studie veranlasst und autorisiert werden.

16. Literaturverzeichnis

1. Rowland LP, Shneider NA. Amyotrophic lateral sclerosis. N Engl J Med. 2001;344:1688-700.
2. Cleveland DW, Rothstein JD. From Charcot to Lou Gehrig: deciphering selective motor neuron death in ALS. Nat Rev Neurosci. 2001;2:806-19.
3. Bensimon G, Lacomblez L, Meininger V. A controlled trial of riluzole in amyotrophic lateral sclerosis. ALS/Riluzole Study Group. N Engl J Med. 1994;330:585-91.
4. Lacomblez L, Bensimon G, Leigh PN, Guillet P, Meininger V. Dose-ranging study of riluzole in amyotrophic lateral sclerosis. Amyotrophic Lateral Sclerosis/Riluzole Study Group II. Lancet. 1996;347:1425-31.
5. McGeer PL, McGeer EG. Inflammatory processes in amyotrophic lateral sclerosis. Muscle Nerve. 2002;26:459-70.
6. Weydt P, Yuen EC, Ransom BR, Moller T. Increased cytotoxic potential of microglia from ALS-transgenic mice. Glia.2004;48:179-82.
7. Hensley K, Fedynyshyn J, Ferrell S, Floyd RA, Gordon B, Grammas P, Hamdheydari L, Mhatre M, Mou S, Pye QN, Stewart C, West M, West S, Williamson KS. Message and protein-level elevation of tumor necrosis factor alpha (TNF alpha) and TNF alpha-modulating cytokines in spinal cords of the G93A-SOD1 mouse model for amyotrophic lateral sclerosis. Neurobiol Dis. 2003;14:74-80.
8. Bruce KM, Narayan K, Kong HC, Larmour I, Lopes EC, Turner BJ, Bertram JF, Cheema SS. Chemotherapy delays progression of motor neuron disease in the SOD1 G93A transgenic mouse. Chemotherapy. 2004;50:138-42.
9. Greig NH, Mattson MP, Perry T, Chan SL, Giordano T, Sambamurti K, Rogers JT, Ovadia H, Lahiri DK. New Therapeutic Strategies and Drug Candidates for Neurodegenerative Diseases: p53 and TNF-alpha Inhibitors, and GLP-1 Receptor Agonists. Ann N Y Acad Sci. 2004;1035:290-315.
10. Clement AM, Nguyen MD, Roberts EA, Garcia ML, Boillee S, Rule M, McMahon AP, Doucette W, Siwek D, Ferrante RJ, Brown RH Jr, Julien JP, Goldstein LS, Cleveland DW. Wild-type nonneuronal cells extend survival of SOD1 mutant motor neurons in ALS mice. Science. 2003;302:113-7.
11. Burls A, Jobanputra P. The trials of anakinra. Lancet. 2004; 364:827-8.
12. Fleischmann RM, Schechtman J, Bennett R, Handel ML, Burmester GR, Tesser J, Modafferi D, Poulakos J, Sun G. Anakinra, a recombinant human interleukin-1 receptor antagonist (r-metHuIL-1ra), in patients with rheumatoid arthritis: A large, international, multicenter, placebo-controlled trial. Arthritis Rheum. 2003;48:927-34.
13. Dinarello CA. Interleukin-1beta and the autoinflammatory diseases. N Engl J Med. 2009;360:2467-70.
14. Meissner F, Molawi K, Zychlinsky A. Mutant superoxide dismutase 1-induced IL-1beta accelerates ALS pathogenesis. Proc Natl Acad Sci U S A. 2010;107:13046-50.
15. Cohen SB, Moreland LW, Cush JJ, Greenwald MW, Block S, Shergy WJ, Hanrahan PS, Kraishi MM, Patel A, Sun G, Bear MB; 990145 Study Group. A multicentre, double blind, randomised, placebo controlled trial of anakinra (Kineret), a recombinant interleukin 1 receptor antagonist, in patients with rheumatoid arthritis treated with background methotrexate. Ann Rheum Dis. 2004;63:1062-68.
16. Cohen S, Hurd E, Cush J, Schiff M, Weinblatt ME, Moreland LW, Kremer J, Bear MB, Rich WJ, McCabe D. Treatment of rheumatoid arthritis with anakinra, a recombinant human interleukin-1 receptor antagonist, in combination with methotrexate: results of a twenty-four-week, multicenter, randomized, double-blind, placebo-controlled trial. Arthritis Rheum. 2002;46:614-24.
17. Cedarbaum JM, Stambler N. Performance of the Amyotrophic Lateral Sclerosis Functional Rating Scale (ALSFRS) in multicenter clinical trials. J Neurol Sci. 1997;152 Suppl 1:S1-9.
18. Kaufmann P, Levy G, Thompson JL, Delbene ML, Battista V, Gordon PH, Rowland LP, Levin B, Mitsumoto H. The ALS-FRS-R predicts survival time in an ALS clinic population. Neurology. 2005;64:38-43.

19. Magnus T, Beck M, Giess R, Puls I, Naumann M, Toyka KV. Disease progression in amyotrophic lateral sclerosis: predictors of survival. Muscle Nerve. 2002;25:709-14.
20. Traynor BJ, Zhang H, Shefner JM, Schoenfeld D, Cudkowicz ME; NEALS Consortium. Functional outcome measures as clinical trial endpoints in ALS. Neurology. 2004;63:1933-5.
21. Brooks BR, Miller RG, Swash M, Munsat TL; World Federation of Neurology Research Group on Motor Neuron Diseases. El Escorial revisited: revised criteria for the diagnosis of amyotrophic lateral sclerosis. Amyotroph Lateral Scler Other Motor Neuron Disord. 2000;1:293-9.

17. Anhänge

1. Revidierte ALS-Funktions-Bewertungsskala (ALS *Functional Rating Scale-Revised*; ALS-FRS-R)
2. Manuelle Muskeltestung (*Manual Muscle Testing*, MMT)
3. Verbale Rating-Skala (VRS)
4. Selbstbewertung der Reaktionen an der Einstichstelle
5. Investigator´s Brochure (siehe Antragsunterlagen)
6. Muster der Patienteninformation (siehe Antragsunterlagen)
7. Muster der Einwilligungserklärung (siehe Antragsunterlagen)
8. Referenzbereichsliste, Institut für Laboratoriumsmedizin und Pathobiochemie, Campus Virchow-Klinikum

Anlage1: **Revidierte ALS-Funktions-Bewertungsskala**
(ALS Functional Rating Scale-Revised - ALSFRS-R)

**1. Sprache**	
4	Normaler Sprachfluss
3	Wahrnehmbare Sprachstörungen
2	Verständlich bei Wiederholung
1	Sprache kombiniert mit nichtverbaler Kommunikation
0	Verlust der verständlichen Sprache
**2. Speichelfluss**	
4	Normal
3	Geringfügig, aber eindeutig mit Übermaß an Speichel im Mund; nach Speichelverlust möglich
2	Mäßiger vermehrter Speichelfluss; geringe Speichelverlust möglich
1	Deutlicher vermehrter Speichelfluss; teilweise mit Speichelverlust
0	Deutlicher Speichelverlust; Taschentuch ständig erforderlich
**3. Schlucken**	
4	Normale Essgewohnheiten
3	Beginnende Essprobleme mit gelegentlichem Verschlucken
2	Änderung der Nahrungskonsistenz
1	Ergänzende Sondenernährung erforderlich
0	Keine orale Nahrungsaufnahme; ausschließlich PEG-Sondennahrung
**4. Handschrift**	
4	Normal
3	Langsam oder unordentlich, alle Wörter lesbar
2	Nicht alle Wörter lesbar
1	Kann Stift halten
0	Kann Stift nicht halten
**5. Gebrauch von Besteck**	
4	Normal
3	Etwas langsam und unbeholfen, aber keine Hilfe erforderlich
2	Kann das Essen meistens schneiden, aber unbeholfen und langsam; bracht teilweise Hilfe
1	Essen muss mundgerecht vorgeschnitten werden, aber kann noch langsam allein essen
0	Muss gefüttert werden
**6. Ankleiden und Körperpflege**	
4	Normale Funktion
3	Unabhängige und vollständige Selbstpflege mit Mühe
2	Zeitweilige Hilfe oder Hilfsverfahren
1	Zur Selbsthilfe ist Hilfspersonal erforderlich
0	Vollständige Abhängigkeit

**7. Umdrehen im Bett und Richten der Bettdecke**	
4	Normal
3	Etwas langsam und unbeholfen, aber keine Hilfe erforderlich
2	Kann sich allein umdrehen oder Bettlaken Zurechtziehen, aber mit großer Mühe
1	Kann die Drehung bzw. das Zurechtziehen der Bettdecke beginnen, aber nicht alleine ausführen
0	Vollständige Abhängigkeit
**8. Gehen**	
4	Normal
3	Beginnende Gehschwierigkeiten durch Schwäche der Beine
2	Deutliche Gangstörung; nur mit Unterstützung oder Gebrauch von Hilfsmitteln möglich
1	Nicht gehfähig, aber gezielte Bewegungen der Beine möglich
0	Keine zielgerichtete Beinbewegung
**9. Treppensteigen**	
4	Normal
3	Langsam
2	Leichte Unsicherheit oder Ermüdung
1	Braucht Unterstützung
0	Nicht möglich
**10. Luftnot**	
4	Keine
3	Beim Gehen
2	Bei Aktivitäten des täglichen Lebens einschließlich Essen, Baden, Ankleiden
1	Leichte Atemnot im Sitzen
0	Schwere Atemnot im Sitzen
**11. Luftnot im Liegen**	
4	Keine
3	Wiederholte nächtliche Luftnot, aber flachen Liegen ist möglich
2	Regelmäßige Verwendung von mehr als 2 Kissen zum Schlafen erforderlich
1	Kann nur im Sitzen schlafen
0	Hochgradige Schlafstörung
**12. Atemhilfen**	
4	Keine Atemhilfe erforderlich
3	Zwischenzeitliche Atemhilfe durch stundenweise Maskenbeatmung (weniger als 8 Stunden)
2	Atemhilfe durch anhaltende Maskenbeatmung in den Nachtstunden (mehr als 8 Stunden)
1	Atemhilfe durch anhaltende Maskenbeatmung in den Tag- und Nachtstunden (mehr als 20 Stunden)
0	Künstliche Beatmung durch Luftröhrenschnitt (Tracheotomie)

Anlage 2: **Manuelle Muskeltestung *(Manual Muscle Testing (MMT)***

Datum der Untersuchung<br>….. …….. ……<br>Tag Monat Jahr	0<br>(Keine Kontraktion)	1<br>(Minimale Kontraktion ohne Bewegungs-effekt)	2<br>(Bewegung unter Auf-hebung der Schwerkraft)	3<br>(Bewegung gegen die Schwerkraft ohne Widerstand)	4<br>(Bewegung gegen die Schwerkraft und einen Widerstand)	5<br>(Normale Kraft)
**Obere Extremitäten**						
Daumenadduktion	Links					
	Rechts					
Fingerbeugung	Links					
	Rechts					
Beugung im Handgelenk	Links					
	Rechts					
Beugung im Ellenbogengelenk	Links					
	Rechts					
Streckung im Ellenbogengelenk	Links					
	Rechts					
Armabduktion	Links					
	Rechts					
	0<br>(Keine Kontraktion)	1<br>(Minimale Kontraktion ohne Bewegungs-effekt)	2<br>(Bewegung unter Auf-hebung der Schwerkraft)	3<br>(Bewegung gegen die Schwerkraft ohne Widerstand)	4<br>(Bewegung gegen die Schwerkraft und einen Widerstand)	5<br>(Normale Kraft)
**Untere Extremitäten**						
Plantarflexion der Zehen	Links					
	Rechts					
Plantarflexion des Fußes	Links					
	Rechts					

Dorsalflexion des Fußes	Links					
	Rechts					
Beugung im Kniegelenk	Links					
	Rechts					
Streckung im Kniegelenk	Links					
	Rechts					
Hüftbeugung	Links					
	Rechts					
Hüftabduktion	Links					
	Rechts					
**Kopfhaltemuskulatur**						
Kopfbeugung						
Kopfstreckung						

Anlage 3: **Verbale Rating-Skala (VRS)**

Im Folgenden finden Sie eine Skala, die einer Selbstbewertung der Kopfschmerzen dient.

Wir bitten Sie, die nächstliegende Antwort zu markieren:

keinen Kopfschmerz	leichten Kopfschmerz	mäßigen Kopfschmerz	starken Kopfschmerz	sehr starken Kopfschmerz	unerträglichen Kopfschmerz

Anlage 4: **Selbstbewertung der Reaktionen an der Einstichstelle**

Im Folgenden stellen wir Ihnen eine Reihe von Fragen, die einer Selbstbewertung der Reaktionen an der Einstichstelle dienen.

Wir bitten Sie, die nächstliegende Antwort zu markieren:

	**Rötung**
0	Keine Rötung
1	Geringgradige Rötung
2	Mittelgradige Rötung
3	Starke Rötung
	**Schwellung**
0	Keine Schwellung
1	Geringgradige Schwellung
2	Mittelgradige Schwellung
3	Starke Schwellung
	**Schmerzen**
0	Keine Schmerzen
1	Geringgradige Schmerzen
2	Mittelgradige Schmerzen
3	Starke Schmerzen
	**Blutung der Haut**
0	Keine Blutung der Haut
1	Geringgradige Blutung der Haut
2	Mittelgradige Blutung der Haut
3	Starke Blutung der Haut
