# Supplementary material for: Interleukin-1 Antagonist Anakinra in Amyotrophic Lateral Sclerosis—A Pilot Study
Source: PLoS One. 2015 Oct 7;10(10):e0139684. doi: 10.1371/journal.pone.0139684 (PMC4596620; doi:10.1371/journal.pone.0139684)
Supplement: S2 File — (PDF) [file pone.0139684.s007.pdf]

## 2. Table of contents

1. Responsibilities ....02
2. Contents ....03
3. Synopsis ....05
4. Flow chart ....21

5.1 Introduction ....25
5.2. Problem and rationale ....31
5.3. Risk-benefit analysis ....32

6. Aims of clinical trial ....33
6.1. Primary endpoint ....33
6.2. Other target criteria ....33
6.3. Study design ....33
6.4. Schedule ....34

7. Patients ....34
7.1. Inclusion criteria ....34
7.2. Exclusion criteria ....34

8. Test materials ....35
8.1. Study medication ....35
8.1.1. Test medication ....35
8.1.1.1. Description of test medication ....35
8.1.1.2. List of side effects and pharmacological interactions ....36
8.1.2. Placebo and Comparative medication ....37
8.2. Treatment plan ....37
8.2.1. Instructions for drug delivery ....37
8.2.2. Instructions for dose adjustment ....38
8.2.2.1 Medical emergency measures ....38
8.3. Concomitant medication....38
8.3.1. Specific designation....38
8.4. Storage, distribution, return and documentation of study medication ....38
8.5. Compliance ....39
8.6. Requirements of trial centers and clinicians involved ....39
8.7. Inclusion and registration ....39
8.7.1. Recruitment logbook ....39
8.7.2. Registration ....39
8.8. Study plan ....40
8.8.1. Clinical examinations and status surveys ....40
8.8.1.1. Initial and screening examinations ....40
8.8.1.2. Follow-up examinations ....42
8.8.1.3. Final examination ....43
8.8.1.4. Laboratory examination ....43
8.9. Duration of study participation for the patient ....43
8.10. Premature drop out of study of the patient ....43
8.11. Premature termination of clinical trial ....44

9. Adverse events ....44
9.1. Definition of adverse events (AE) ....44
9.2. Definition of serious adverse events (SAE) ....45
9.3. Assessment of intensity of adverse events ....45
9.4. Assessment of causal link ....46

9.5. Documentation of adverse effects ....................................................................46
9.6. Notification of serious and/or adverse events ....................................................46
9.7. Follow-ups after Serious Adverse Events (SAE)................................................47
9.8. Notification of Suspected Unexpected Serious Adverse Reaction, (SUSAR) ....47
9.9. Pregnancy ..........................................................................................................48
9.10. Contacts for notification of SAE .......................................................................48

10. Documentation ....................................................................................................48
10.1 Case report forms (CRF) ...................................................................................48
10.2. Investigator site file ..........................................................................................48
10.3. Internet portal ....................................................................................................49

11. Quality management ...........................................................................................49
11.1 Monitoring of study course and data quality ......................................................49
11.1.1 Monitoring .......................................................................................................49
11.1.2. Audit ...............................................................................................................49
11.2. Standardization and validation ..........................................................................49
11.3. Reference institutions .........................................................................................50

12. Input and management of data ............................................................................50
12.1. Data collection and documentation forms ..........................................................50
12.2. Data processing ...................................................................................................51
12.3. Generation of pseudonyme ..................................................................................51

13. Statistical analysis ..................................................................................................52
13.1. Assessment of case numbers ...............................................................................52
13.2. Statistical analysis ...............................................................................................52
13.2.1 Hypotheses .........................................................................................................52
13.2.2. Definition of evaluation population .....................................................................52
13.2.3. Evaluation of primary and secondary target parameters ...................................52
13.2.4. Evaluation of safety ...........................................................................................52
13.2.5. Possible interim analyses and premature termination of clinical trial .............53
13.2.6. Evaluation methods ...........................................................................................53

14. Reporting ...............................................................................................................53
14.1. Protocols ...............................................................................................................53
14.2. Audit report ...........................................................................................................53
14.3. Final report and publication ..................................................................................53

15. Ethical, legal and administrative aspects ..............................................................53
15.1. Legal requirements for the study ..........................................................................53
15.2. Patient information and informed consent ...........................................................54
15.3. Patient insurance ...................................................................................................54
15.4. Data protection and medical confidentiality..........................................................54
15.5. Filing of data ..........................................................................................................54
15.6. Legal aspects .........................................................................................................54
15.7. Good Clinical Practive (GCP) ................................................................................55
15.8. Funding of the study ..............................................................................................55
15.9. Compliance with the protocol and protocol amendments ....................................55

16. References ................................................................................................................56

17. Annexe .......................................................................................................................58

Scientific background for the study: ALS is a neurodegenerative disease of adult onset caused by selective degeneration of motor neurons in the cortex and the spinal cord. The degeneration of motor neurons results into progressive paralysis of the limbs, speech, swallowing and respiratory muscles. ALS is always fatal and patients die of respiratory insufficiency between 3 and 5 years after diagnosis. So far, Riluzol (Sanofi-Synthelabo-Aventis) is the only approved medication that results into a minor prolongation of life (1.5 to 2.5 months). Riluzol is an inhibitor of the presynaptical release of glutamate. Another effect of Riluzol is the inhibition of glutamate release in activated microglia.

Neuroinflammation is an essential pathogenicity factor of motor neuron degeneration. Inflammatory changes can be found in sporadic ALS, the autosomal dominant form of ALS as well as in the SOD1-mouse model of the ALS. This can be demonstrated morphologically through the accumulation of activated microglia, astrocytes, and T-cells. At the biochemical level, these changes are accompanied by the activation and release of the proinflammatory cytokine Interleukin (IL)-1. The inflammatory mediator IL-1 mediates the recruitment and activation of phagocytic cells and induces an immune response. The bioactivity of IL-1 is physiologically controlled by the naturally occurring, highly selective Interleukin-1-Receptor-Antagonist (IL-1RN).

On the basis of the neuroinflammatory changes in ALS there is a scientific rationale for a clinical trial with ANA, which has anti-inflammatory pharmacological effects. ANA is a recombinant IL-1RN (r-metHuIL-1RN) which directly and selectively inhibits IL-1.

Page 6 in the German text

The primary study target implies the safety and tolerance of ANA in combination with Riluzol in ALS patients. An open tolerability study aims at testing whether a subcutaneous injection of 100 mg ANA is tolerated. This tolerability study will be the basis of a phase IIb/III efficacy study.

Tested medication: Trial medication: ANA (Kineret®) 100 mg of injectable solution in a ready-to-use pre-filled syringe in combination with an oral dosis of 100 mg Riluzol. Open treatment with 100 mg ANA + 100 mg Riluzol.

Comparative medication: none

Study design: One arm study to assesst the safety and tolerance of 100 mg ANA for the duration of 52 weeks in combination with Riluzol.

Schedule: Recruitment of patients: 11-2010 until 04-2011; duration of study: 52 weeks. Termination of clinical trial: 04-2012

Total number of patients: Treatment of 20 patients with 100 mg ANA in combination with 100 mg Riluzol. The biometric determination of cases numbers results into 17 analyzable patients, allowing a statistically proven effect of the primary endpoint. A screening of 30 – 60 patients is required to recruit 20 patients for the study.

Total number of centers: 1

Study population: patients with Amyotrophic Lateral Sclerosis (ALS)

Inclusion criteria

- patients aged ≥ 18 years and ≤ 80 years.

• clinical diagnosis of ALS (revised El Escorial criteria) with dominant affection of the 2nd motor neurons or the clinical ALS variant of a progressive muscle atrophy (PMA).
• clinical signs of a dysfunction of the 2nd motorneuron in at least one anatomic region outside the bulbar region.
• sporadic and familiar ALS.
• onset of symptoms with pareses from six months up to four years before inclusion in the study.
• treatment with Riluzol 100 mg/day at least for 3 months before termination of study.
• written informed consent (according to AMG §40 (1) 3b).
• ability to communicate, either in person or by an authorized person.
• ability to communicate, either verbally, manually or through an electronic communication system.
• internet access, either personally or by an authorized person.
• refrigerator facilities for ready-to-use pre-filled ANA-syringes at home.

Page 7 in the German text

• no participation in any other clinical trial during this study schedule.
• negative pregnancy test or at least 24 months postmenopausal.

Exclusion criteria:
• diagnosis of ALS with dominant affection of the 1st motor neurons without clinical signs for a concomitant dysfunction of the 2nd motor neurons in at least one anatomic region outside the bulbar region (clinical variant of a spastic ALS).
• diagnosis of a clincal ALS-variant of a primary lateral sclerosis (PLS).
• patienten with known hypersensitivity to ANA, Riluzol or one of the adjuvants.
• clinical severe hypoventilation syndrome with a vital capacity < 50 %.
• pregnant or breast-feeding women.
• women of childbearing age who are not using or cannot use any highly effective contraceptives (Pearl-index <1).
• clincal significant modifications of the ECG including symptomatic bradycardia.
• continuous non-invasive ventilation with a ventilation free period < 2 hours.
• Tracheotomy and mechanical ventilation.
• Laboratory parameters outside the standard range and equivalent to a clinical significant. cardiovascular, pulmonological, hematological, hepatological, metabolic and kidney disease.
• malignant tumors.
• severe renal impairment (creatinine-clearance < 30 ml/min).
• previous history of recurrent infection or an underlying disease that predisposes to infections.
• severe neutropenia (absolute number of neutrophils < 1,5 x $10^{9}$/l).
• monoclonal gammopathy of uncertain significance.
• infectious diseases including tuberculosis, HIV, hepatitis B and C.
• patients with severe clinical co-morbidities including psychiatric diseases.
• dementia and lack of capacity for consent.
• clinicial diagnosis of epilepsy or an epileptic seizure.
• treatment with any other study medication < 3 months prior to study inclusion.
• diseases or malfunctions which exclude participation at the study at the treating investigator’s discretion.
• non-cooperation.
• all contraindications concerning the study medication (inclusive adjuvants of the dosage form).
• non-cooperation regarding storage and disclosure of pseudonomized data in the context of the clinical trial.

Page 8 in the German text

• combined use of ANA and Etanercept or other TNF-antagonists.
• internment at a judicial institution (according to AMG §40 (1) 4).

Page 9 in the German text

Targets: The primary target of the study is the examination of safety and tolerability of a subcutaneous injection of ANA together with an oral dosis of Riluzol in ALS patients.
The choice of sample size is not appropriate for securing the effectiveness of ANA in combination with Riluzol in ALS. However, by means of secondary endpoints the influence of the ANA treatment on the ALS progression rate and life quality will be studied.

*Primary endpoints:*
Examination of number and severity of
• adverse events (AE).
• severe adverse events (SAE).
• adverse drug reactions (ADR).
• unexpected adverse drug reactions (uADR).
• serious adverse drug reactions (sADR).
• suspected cases of unexpected serious adverse drug reactions (SUSAR).
• pathological laboratory parameters.

*Secondary endpoints:*
• evaluation of long term tolerance and pharmaceutical drug safety of ANA in combination with Riluzol.
• number of patients in need of a continued, non-invasive ventilation or tracheostoma supported invasive ventilation.
• number of patients in need of a percutaneous endoscopic gastrostomy (PEG.)
• number of patients who have completed a treatment with ANA in.combination with Riluzol.
• evaluation of the clinical efficacy of ANA on the progression rate of pareses and functional limitations in ALS by comparison to the evaluation of the intra-individual progression rate of the revised *ALS Functional Rating Scale* (ALS-FRS-R) over a study period of 52 weeks and the progression rate prior to the participation in the study (Δ ALS-FRSR).
• evaluation of the efficacy of ANA on the respiratory function in ALS, which is determined by a forced vital capacity in the course of 52 weeks.

Page 10 in the German text

• evaluation of the efficacy of ANA on the respiratory function in ALS patients, who are subject to a non invasive mask ventilation (determination of ventilation time per day).
• determination of survival time or timing of a tracheotomy because of respiratory insufficiency (apart from elective tracheotomy or to control secretions).

Safety
Information, consent, and documentation:
• the patient will be given written information on the application of the medication, its risks, the importance of a contraception, and appropriate methods for safe contraception.
• written consent of the patient to participate in the study.
• written confirmation from the patient that the information on the study has been read and understood.
• documentation of information and written consent.

Women in childbearing age who did not have a hysterectomy, and whose menopause was less than 24 months before the initiation of the study will have to have a pregnancy test prior to treatment with the study. Women in childbearing age also have to ise a highly effective

contraception method (defined as Pearl Index < 1) (pill, contraceptive coil, hormone implant, transdermal patch, a combination of two barrier methods (condom and diaphragm)), a sterilization or sexual abstinence at least one month prior to the first application of the study medication and for the whole duration of the treatment.

Measures to be taken prior to the treatment of women in childbearing age
• pregnancy test in the urine
• discontinuation of therapy in case of proof of pregnancy

Page 12 in the German text

<u>Termination criteria</u>
Termination or premature discontinuation of the study will be documented for each patient. The reason for the termination will be examined and documented. The following criteria can be sufficient for the discontinuation of the study:

• laboratory parameters outside the normal range, e. g.
    o neutropenia (ANZ < 1,5 x 109/l).
    o severe renal impairment (CLCr < 30 ml/minute).
    o ALT/AST values are more than 5 fold higher than the upper limit.
• pregnancy.
• significant violation of study plan .
• lack of compliance.
• severe infection.
• clinical signs for an allergic reaction including maculopapular or urticarial rashes or unclear increase in body temperature.
• severe skin reactions, especially bullous skin reactions including Stevens-Johnson-syndrome and toxic-epidermal necrolysis
• pathological examination findings, including electrocardiography.
• personal request of the patient.
• withdrawal of informed consen.t
• loss of contact.
•if the patient moves to another place and can no longer participate in the study.

Page 13 in the German text

• death of the patient

<u>Termination of the study</u>:
A premature termination of the study is possible under the following conditions:
• a patients suffers of a severe infection which is possibly, likely, or certainly caused by the study medication and cannot be explained in the context of the underlying clinical condition.
• new and reproducibly scientific findings on ANA during the course of the clinical study show the inferiority of the treatment with ANA
• evidence of increasing number of serious adverse events

The PI of the clinical trial will decide on the termination of the study.
After termination the following events will be documented and communicated to the sponsor of the study:
• all serious adverse events which have incurred within 30 days after the last treatment with ANA

• Any pregnancy which has been confirmed within 84 days (12 weeks) after the last dosis of ANA .

Statistical evaluation:
The primary objective of this pilot study is tolerability and the secondary endpoints should allow, concurrently, to establish conditions to plan a statistically justifiable phase II efficacy study, is a proof of tolerability and several secondary endpoints.
Primary and several secondary endpoints will first be examined exploratively and evaluated descriptively. The conceptual study will be carried out with n=20 patients.
Similar scientific questions have been described in the literature with a comparable number of patients. Secondary endpoints will be examined by parametric or non-parametric tests depending on scaling and distribution type. The biometric evaluation will be done by SAS®.

Page 33 in the German text

**6. Aims of the clinical trial**
The primary target of the study is the examination of safety and tolerability of a subcutaneous injection of ANA combined with Riluzol in ALS patients. The chosen sample size is not appropriate for proving the effectiveness of ANA in combination with Riluzol in ALS. However, by means of secondary study endpoints the influence of ANA on the ALS progression rate can be investigated.

**6.1. Main target criterion**
*Primary objective of the study:*
Examination of number and severity of adverse events (UE), severe adverse events (SUE), adverse drug reactions (ADR), unexpected adverse drug reactions (uADR), severe adverse drug reactions (SADR), Suspected Unexpected Serious Adverse Reaction (SUSAR), and pathological laboratory parameters.

**6.2. Other criteria**
*Secondary objectives of the study:*
• Evaluation of long term tolerability and drug safety of ANA in combination with Riluzol.
• Number of patients in need of continuous non-invasive ventilation or tracheostoma supported invasive ventiliation.
• number of patients in need of a percutaneous endoscopic gastrostomy (PEG).
• number of patients who have concluded a treatment with ANA in combination with Riluzol.
• evaluation of clinical effectiveness of ANA on the progression rate of pareses .
and functional impairments in ALS by comparative analysis of the intraindividual progression rate of the revised *ALS Functional Rating Scale* (ALS-FRS-R) in a study period of 52 weeks compared to the progression rate before participation in the study (Δ ALS-FRS-R).
• examination of effectiveness of ANA on the respiratory function in ALS which is assessed by a forced vital capacity in the course of 52 weeks.
• examination of effectiveness of ANA on the respiratory function in ALS patients in need of a non-invasive mask ventilation (determination of ventilation time per day).
• determination of survival time or timing for a tracheotomy by respiratory insufficiency (exclusive elective tracheotomy or tracheotomie for control of secretion).

**6.3. Study design**
The study design is to evaluate of safety and tolerability of 100 mg ANA in combination with Riluzol for the duration of 52 weeks in.

*Study characteristics*
The project aims to evaluate tolerability and safety. In rheumatoid arthritis several pilot studies, dose determination studies, and phase II/III studies with ANA have been carried out. So far. there are no data available regarding safety and tolerability of ANA in

neurodegenerative diseases. In our study, first information on tolerability of ANA in ALS will be generated.

*Study organization:*
This study will be carried out in a single study center. It will include 20 patients. The patients will be recruited monocentrically. From the experience from past ALS therapy studies, 30 ALS patients will have to be screened to recruit the planned 20 patients. Also from the experience of past and published ALS studies a drop-out rate of a maximum of 10 percent can be expected; a number which is already included in the calculation of patients required. More than 50 percent of the intended number of patients can be recruited from patients of the ALS ambulance of the sponsor. More patients can be recruited via a patients' information on the website of the ALS ambulance of the Charité (www.als-charite.de). Between 11/2002 – 02/2010 six phase II studies were completed in this study center, comprising a total of 165 study participants. Previous experiences shown that a recruitment of ALS patients is realistic because of established information channels such as websites, publications, self-help organizations, media information.
This allows a screening of 30 ALS patients in a recruitment period of 6 months.
In Germany the number of ALS patients is believed to be 6,000 and the number of new cases is 1,500 per year. In the study center 50 % of the patients are from Berlin and Brandenburg states whereas the other half is from the other states. This patient structure allows an inter-regional recruitment of patients.

Page 34 in the German text

## 7. Patients

### 7.1. Inclusion criteria:

- patients aged ≥ 18 years and ≤ 80 years.
- clinicial diagnosis of ALS (revised El Escorial criteria) with dominant affection of the 2nd motor neuron or clinical ALS variation of a progressive muscle atrophy (PMA).
- clinical signs for a dysfunction of the $2^{nd}$ motor neuron in at least one anatomical region outside bulbar region.
- sporadic and familiar ALS.
- onset of symptoms with pareses six months and up to four years prior to study inclusion.
- treatment with Riluzol 100 mg/day at least 3 months prior to study inclusion.
- written informed consent (according to AMG §40 (1) 3b).
- ability to communicate, either personally or by an authorized person.
- ability to communicate, verbally, manually or by an electronic communication system.
- internet access, either personally or by an authorized person.
- refrigerating capacity for the pre-filled ANA-syringes at home.
- no participation in any other study .
- negative pregnancy test or at least 24 months postmenopausal

### 7.2. Exclusion criteria:

- diagnosis of ALS with dominant affection of $1^{st}$ Motor neurons without clinical signs for a concomitant dysfunction of the $2^{nd}$ motor neurons in at least one anatomic region without the bulbar region (clinical variant of a spastic ALS).
- diagnosis of a clincal ALS-variant of a primary lateral sclerosis (PLS)

Page 35 in the German text

- patients with a known hypersensitivity to ANA, Riluzol or one of the adjuvants.
- clinical severe hypoventilation syndrome with a vital capacity < 50 %.
- pregnant or breast-feeding women.
- women of childbearing age who are not using or cannot use any highly effective contraceptives (Pearl-index <1).

• clincal significant modifications of the ECG including symptomatic bradycardia
• continuous non-invasive ventilation with a ventilation free period < 2 hours
• Tracheotomy und mechanical ventilation
• Laboratory parameters outside the standard range and equivalent to a clinical significant cardiovascular, pulmonological, hematological, hepatological, metabolic and renal disease.
• malignant tumors.
• severe renal impairment (creatinine-clearance < 30 ml/min).
• previous history of recurrent infection or an underlying disease that predisposes to infections.
• severe neutropenia (absolute number of neutrophils < 1,5 x $10_{9/}$l).
• monoclonal gammopathy of uncertain significance.
• infectious diseases including tuberculosis, HIV, hepatitis B and C.
• patients with severe clinical co-morbidities including psychiatric diseases.
• dementia and lack of capacity for consent.
• clinicial diagnosis of epilepsy or an epileptic seizure.
• treatment with any other study medication < 3 months prior to study inclusion.
• diseases or malfunctions which exclude participation at the study at the treating investigator's discretion.
• non-cooperation.
• all contraindications concerning the study medication (inclusive adjuvants of the dosage form)
• non-cooperation regarding storage and disclosure of pseudonomized data in the context of the clinical tria.l
• combined use of ANA and Etanercept or other TNF-antagonists
• accommodation at an institution at judicial or official request (according to AMG §40 (1) 4)

Page 37 in the German text

**8.2. Treatment regimen**
**8.2.1. Instructions for application:**
**Dosage, timing and duration of administration of ANA:**
The trial consists of a continuous treatment with 100 mg Riluzol and a dose of 100 mg ANA/day for the duration of 52 weeks. The medication is consumed daily (1 pre-filled Kineret 100 mg syringe). The patients receive a combination treatment with 100 mg Riluzol. The treatment with Riluzol is carried out as basic medication and pre-treatment during the whole period of treatment, the dose remains unchanged.

ANA is supplied by the Swedish company Orphan Biovitrum AB. The pharmacy at the study center supplies the drug to the patient's address and is in charge of maintaining the cold chain. At home the drug is kept in the refrigerator (2°C - 8°C). The patient will receive an individual injection training in the study center before start of the treatment.

**Measures required for contraception:**
Female patients who indicated that they did not have a hysterectomy or whose menopause is not longer than 24 months ago, have to get medical advice by a physician regarding risk assessment of a pregnancy and – where appropriate - regarding contraceptives. These patients have to use reliable contraceptives at least one month before start of treatment with ANA, during the whole treatment period, and one month after termination of treatment. Reliable contraceptives are the simultaneous use of a highly effective method of contraception such as intrauterine pessary, hormonal contraceptives, tubal ligation, vasectomy in the partner) and at least one additional effective method (diaphragm, pessary, cervical cap, or latex condom in the male partner).

**Prescription procedure for ANA:**
The safety requirements of the announcement of the Drug Commission of the German Medical Association imply the following measures:

• detailed information to the patient.
• therapy risks.
• ANA must not be distributed to other persons.

Page 38 in the German text

• The patient most not donate blood during and at least one month after termination of ANA treatment

**Information, consent and documentation:**
• the patient is given written information on the application, the risks of application, the necessity of safe contraception.
• written consent of the patient to the treatment.
• the patient has to confirm in writing that she/he has been informed and understood all information.
• documentation of information and consent.

**8.4. Storage, distribution, return and documentation of study medication**
The drug is commercially available from the cooperating pharmacy. Storage, cold chain, supply, distribution and return will be taken care of by the cooperating pharmacy or the

Page 39 in the German text

investigator. The receipt of the medication will be acknowledged by the investigator, the pharmacist, and the patient. After delivery, the medication will be stored at the patient's home in a refrigerator. Empty boxes and unused units of the drug will be collected by the investigator. The investigator will return unused units of the drug to the pharmacy where they will be destroyed. Transmission, consumption, loss, non-use, remainders of the drug will be documented with the following specifications:
• prescription, consumption, non-use or loss of drug.
• date of distribution and receipt of drug.
• amount of drug.
• coding number of patient (apart from remainders).

The test drug must only be used for study purposes and must not be used outside the study schedule. The patients are obliged to return unused units upon every visit to the study center. The study drug will be kept at the cooperating pharmacy before distribution.

**8.5. Compliance**
The treatment with the drug will be carried out on an out-patient basis. The following measures for supervision and documentation of compliance will be made:
• the treatment of the patient will be monitored in monthly telephone visits and weekly internet visits.

**8.6. Requirements for study centers and study doctors:**
The required number of 20 patients can be recruited from the patients of the study center and additional recruitment. The majority of patients were diagnosed with ALS in an external neurological hospital, and the diagnosis was verified and confirmed in the study center. The study center has all diagnostic requirements for making an initial and confirmatory diagnosis of ALS (electromyography, electroneurography, cerebrospinal fluid diagnostics, MRT diagnostics). None of these aforementioned diagnostic measures are, however, required for inclusion into the study. Therfore, no invasive (electromyography, cerebrospinal fluid diagnostics) or imaging (MRT) examinations are required.

**8.7. Admission and registration**
**8.7.1. Registration logbook**

A logbook for the recruitment will be set up in which all patients meeting the inclusion criteria are consecutively recorded. All reasons for a non-inclusion will be documented. Patients will be recruited from the patients of the study center and by announcement on the website of the study center. A proposal for an internet announcment of the study is attached in the annexe. The internet address of the ALS ambulance of the study center is www.als-charite.de.

**8.7.2. Registration**

Patients who meet all inclusion criteria and who do not meet any of the exclusion criteria and who have given their consent to the study will be registered on a registration form with the following data:

- name and address of study center.
- name of investigator in charge.
- phone, fax, and e-mail of investigator.
- pseudonym of patient.
- sex.
- age.
- duration of disease (in months).
- disease variation (bulbar variation or limb variation).
- affection of 2nd motor neuron (progressive muscle atrophy (PMA)).
- ALS-FRS.
- progression rate (ALS-FRS/progression [in months]).
